# Trifluoromethyl-Benzimidazole-Hydrazone Derivatives as Promising Selective Anticancer Agents: Synthesis, Cytotoxicity, and Molecular Docking Insights

**DOI:** 10.3390/ph19081270

**Published:** 2026-08-11

**Authors:** Musa Özil, Eyüp Önelge, Mustafa Emirik, Kübra Acikalin Coskun, Yusuf Tutar

**Affiliations:** 1 Department of Chemistry, Recep Tayyip Erdogan University, Rize 53100, Türkiye; 2 Division of Medicinal Biology, Department of Basic Medical Sciences, Istanbul Aydın University, Istanbul 34295, Türkiye; 3 Division of Medical Biochemistry, Department of Basic Medical Sciences, Faculty of Medicine, Recep Tayyip Erdogan University, Rize 53100, Türkiye

**Keywords:** benzimidazole, microwave, synthesis, cytotoxicity, breast cancer, anticancer

## Abstract

**Background/Objectives**: Benzimidazole and hydrazone scaffolds are widely investigated in anticancer drug discovery because of their structural versatility and capacity to interact with multiple cancer-related targets. This study aimed to synthesize a series of trifluoromethyl-substituted benzimidazole-hydrazone derivatives, evaluate their antiproliferative activity and cancer-cell selectivity, and explore their potential interactions with breast cancer-associated molecular targets. **Methods**: A series of benzimidazole-hydrazone derivatives were prepared through a four-step synthetic route using both conventional and microwave-assisted procedures. The structures of the synthesized compounds were characterized using spectroscopic and analytical methods. Antiproliferative activity was evaluated in estrogen receptor-positive MCF-7 human breast cancer cells using the MTT assay after 48 h of exposure. Selected active compounds were additionally tested against non-tumorigenic hTERT cells to determine their selectivity indices. In silico analyses were performed against estrogen receptor alpha in antagonist and selective estrogen receptor degrader conformations, bromodomain-containing protein 4, and the mTOR kinase domain. **Results**: Microwave irradiation substantially reduced reaction times from 12–24 h to 4–6 min and increased isolated yields by 7–24 percentage points compared with conventional conditions. Compounds **4**, **7**, **9**, and **11** reduced MCF-7 cell viability below 50% at 20 μM. Compound **7** showed the highest antiproliferative activity, with an IC_50_ value of 7.5 ± 0.8 μM, and the greatest selectivity toward MCF-7 cells over hTERT cells, with a selectivity index of 12.66. Molecular docking, MD simulation, and MM/GBSA calculations predicted that compound **7** would effectively bind to the investigated targets, particularly estrogen receptor alpha, as well as the BRD4 and mTOR kinase domains. **Conclusions**: Compound **7** represents a selective, mid-micromolar benzimidazole-hydrazone lead against MCF-7 breast cancer cells. Its predicted molecular interactions warrant further target-based, mechanistic, pharmacokinetic, and in vivo evaluation.

## 1. Introduction

Cancer remains a leading health challenge worldwide, and despite the availability of numerous therapeutic agents, treatment success is often curtailed by the emergence of drug resistance [1,2]. This has underscored the need for continued research into new anticancer compounds and strategies, including agents that can concurrently modulate multiple biological targets to overcome resistance [2,3]. In the search for such agents, heterocyclic scaffolds have played a pivotal role in drug discovery. In particular, the benzimidazole ring system has emerged as a privileged pharmacophore in medicinal chemistry, appearing in a multitude of bioactive molecules and approved drugs. This planar bicyclic moiety is renowned for its broad spectrum of biological activities [4], encompassing antimicrobial, antiparasitic, anti-ulcer, and anticancer properties [5,6]. The versatility of the benzimidazole nucleus—capable of accommodating diverse substituents and functional groups—makes it an attractive template for designing new therapeutics [5].

**Figure 1:**
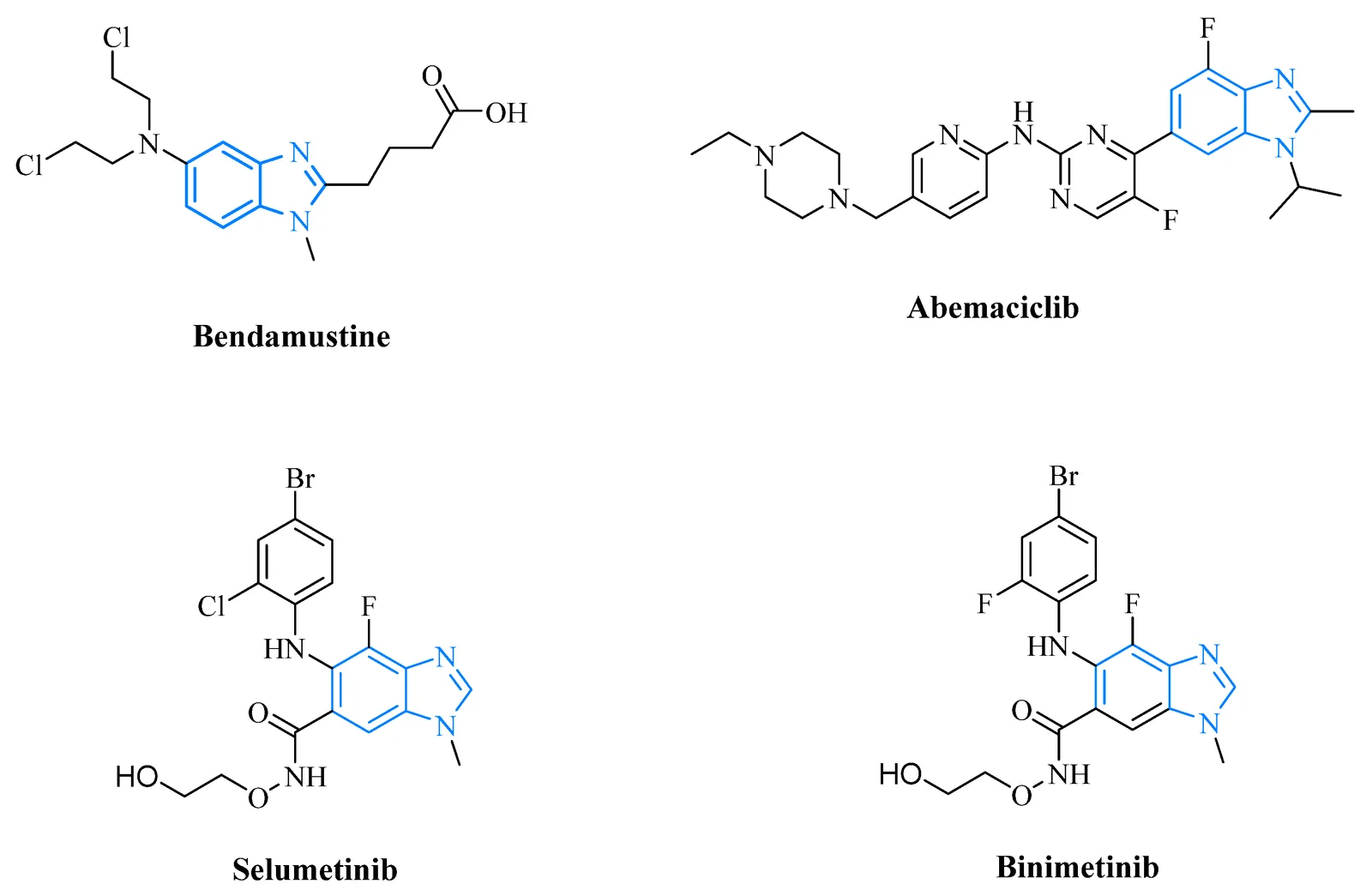
Representative anticancer agents bearing benzimidazole moieties.

A growing body of evidence illustrates the anticancer potential of benzimidazole derivatives, including many examples with activity against breast cancer models. For instance, Messaoudi et al. reported a series of benzoyl–aryl benzimidazoles that exhibited sub-micromolar potency against MCF-7 breast cancer cells (IC_50_ 0.06–0.5 μg/mL) [6]. Another study by Abbade et al. described alkylsulfonyl benzimidazole analogues with notable cytotoxicity in MCF-7 cells (IC_50_ 4.7–10.9 μM), attributed to the inhibition of the anti-apoptotic protein Bcl-2 [7]. MCF-7 cells represent estrogen receptor-positive (ER^+^) breast cancer, one of the most prevalent molecular subtypes, and are widely used as a model system to investigate hormone-responsive tumor biology and therapeutic response. Benzimidazole-based compounds have also been designed to interfere with critical enzymes and structural proteins in cancer cells. For example, a 2-(2-pyridyl)-benzimidazole linked to a 1,2,4-triazole showed potent activity (IC_50_ ~4.56 μM) in lung carcinoma (A549) cells, accompanied by significant inhibition of DNA topoisomerase I. Likewise, benzimidazole-carboxamide hybrids have demonstrated selective cytotoxicity by disrupting tubulin polymerization in melanoma cells. These and other findings highlight the benzimidazole scaffold as a versatile framework for anticancer drug design, capable of yielding agents that act through multiple molecular pathways [7]. Representative examples of anticancer compounds and clinically used drugs featuring a benzimidazole scaffold and/or a trifluoromethyl moiety are presented in Figure 1.

The wide-ranging bioactivities of benzimidazole derivatives stem from their ability to interact with diverse cellular targets. One such target class is the sirtuin family of NAD^+^-dependent deacetylases (class III HDACs). Sirtuins (SIRT1–7) regulate cellular stress responses and are often overexpressed in tumors, where they contribute to oncogenesis by deacetylating and inactivating tumor-suppressor proteins (e.g., FOXO transcription factors, NF-κB, and p53). Recent studies have demonstrated that benzimidazole-based inhibitors can effectively target sirtuins: in fact, a benzimidazole derivative termed BZD9Q1 was identified as a pan-SIRT1–3 inhibitor that exhibited strong antiproliferative activity in cancer cell assays [3]. This underscores the potential of epigenetic mechanisms (such as SIRT-mediated pathways) as a therapeutic avenue accessible to appropriately functionalized benzimidazoles.

Another critical signaling axis in cancer is the phosphatidylinositol 3-kinase (PI3K)/Akt/mTOR pathway, which is frequently dysregulated in solid tumors including breast cancer. Hyperactivation of PI3K/Akt signaling promotes unchecked cell growth, survival, and proliferation while suppressing apoptotic mechanisms [8]. Several benzimidazole-containing derivatives have been reported to inhibit PI3K or related kinases, resulting in reduced Akt phosphorylation and induction of apoptotic cell death [8]. Such observations indicate that benzimidazole-based molecules may serve as modulators of key oncogenic signaling cascades [9].

In addition to enzyme targets and kinases, benzimidazole derivatives have shown promise against epigenetic “reader” proteins such as bromodomain-containing protein 4 (BRD4). BRD4 is a member of the BET bromodomain family that binds to acetylated histone tails and drives the expression of oncogenes (notably c-Myc) crucial for cancer cell proliferation.

Within this context, the incorporation of a hydrazone linkage (–C=N–NH–) into benzimidazole derivatives presents an intriguing design strategy for new anticancer agents. Hydrazone linkages are commonly used in medicinal chemistry to connect two bioactive motifs, and they can endow compounds with unique biochemical features—such as the ability to chelate metal ions or undergo pH-sensitive hydrolysis—that may be advantageous in the tumor microenvironment. Indeed, recent studies on benzimidazole-hydrazone hybrids have shown that this structural motif can enhance anticancer activity. Bostancı et al. described the synthesis of a series of benzimidazole-based hydrazones that were cytotoxic against multiple cancer cell lines; notably, one lead compound was more potent than cisplatin in killing MCF-7 breast cancer cells in vitro [10]. Moreover, these hydrazone-linked derivatives exhibited inhibitory activity against carbonic anhydrase IX (CA IX), an enzyme that helps tumors adapt to hypoxic conditions, suggesting a dual role in directly suppressing cell growth and modulating the tumor microenvironment [10]. Such findings provide a strong rationale for designing new benzimidazole-hydrazone conjugates as potential anticancer agents.

The trifluoromethyl (–CF_3_) group has emerged as a key pharmacophore in medicinal chemistry due to its profound influence on drug metabolism, binding affinity, and pharmacokinetics. Its pronounced electron-withdrawing character, high lipophilicity, and enhanced metabolic stability render it a particularly attractive structural motif for the design of anticancer agents. Numerous FDA-approved anticancer agents, such as sorafenib, alpelisib, and apalutamide, incorporate the –CF_3_ moiety, benefiting from enhanced molecular interactions with cancer-related targets including PI3K/Akt, VEGFR, and EGFR pathways [11,12]. Studies have shown that the inclusion of –CF_3_ groups can improve cytotoxic activity through multipolar interactions and increased cellular permeability. For example, urea derivatives bearing aromatic trifluoromethyl substituents demonstrated potent activity across multiple human cancer cell lines, in some cases surpassing the efficacy of doxorubicin [13]. These attributes underscore the significance of the –CF_3_ group as a versatile and effective tool in the development of next-generation anticancer agents.

In light of the above, the current study focuses on the synthesis and anticancer investigation of novel benzimidazole-based hydrazone derivatives, with particular emphasis on their cytotoxic effects on breast cancer cells (MCF-7 line). By integrating the benzimidazole pharmacophore with a hydrazone linkage, we aimed to create hybrid molecules capable of engaging multiple targets or pathways relevant to breast cancer. In this study, we report the design and chemical synthesis of these compounds, and we report their cytotoxic efficacy in MCF-7 cells along with initial insights into their mechanisms of action. This study seeks to advance benzimidazole-hydrazone hybrids as promising candidates in the ongoing effort to discover more effective treatments for breast cancer, especially in the face of drug-resistant disease. In addition, the selective cytotoxicity of the synthesized compounds toward cancer cells relative to a non-tumorigenic cell line was evaluated.

## 2. Results and Discussion

### 2.1. Chemistry

**Scheme 1:**
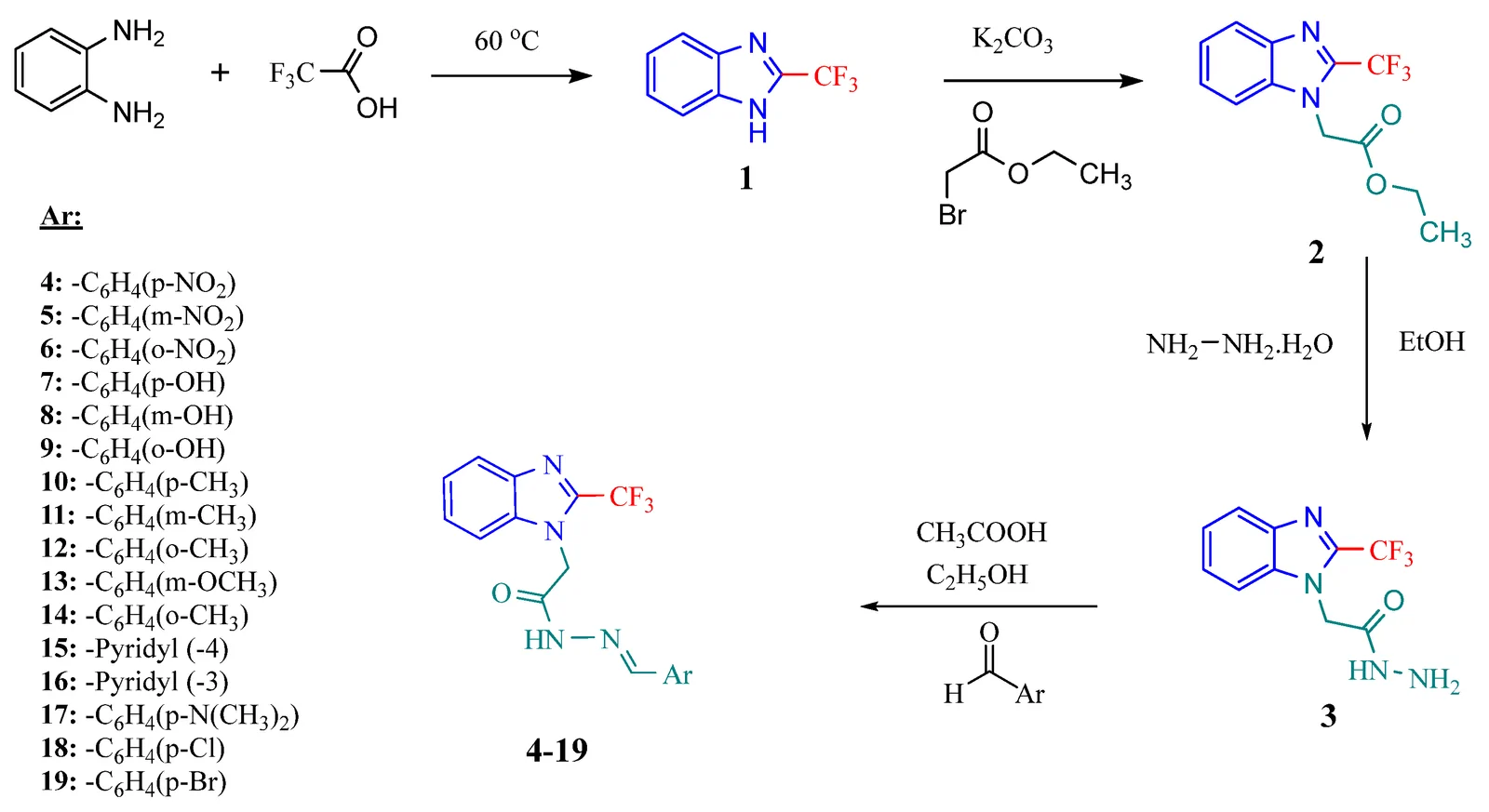
Synthetic pathway for the benzimidazole-hydrazone derivatives.

A series of variously N′-substituted-2-(2-(trifluoromethyl)-1H-benzo[d]imidazol-1-yl)acetohydrazide derivatives were prepared in four-step chemical transformations. The first reaction was the cyclocondensation reaction between o-phenylenediamine and trifluoroacetic acid under reflux to afford 2-(trifluoromethyl)-1H-benzo[d]imidazole (**1**). In the second step of the reaction scheme, compound **1** was reacted with ethyl bromoacetate under basic conditions to produce ethyl 2-(2-(trifluoromethyl)-1H-benzo[d]imidazol-1-yl)acetate (**2**). In the third stage, compound **2** was further treated with an excess of hydrazine hydrate to produce 2-(2-(trifluoromethyl)-1H-benzo[d]imidazol-1-yl)acetohydrazide (**3**) [14]. In both steps, reactions were carried out with microwave irradiation in addition to the conventional method. With the microwave irradiation method, reaction yields increased, and reaction times were considerably shortened. In addition, since the use of solvent is considerably reduced, it emerges as an environmentally friendly method. In the last step of our study, target structures with hydrazone linkage (–C=N–NH–) into benzimidazole derivatives were synthesized by reacting compound **3** with a range of aromatic aldehydes in the presence of a catalytic amount of acetic acid. In this step, all reactions were carried out by the conventional synthesis method in addition to the microwave method (Scheme 1). The chemical structure of the obtained compounds was confirmed by the use of spectroscopic techniques.

Microwave irradiation markedly shortened the reaction times from several hours to only a few minutes (Table 1) while consistently improving the isolated yields, an effect that can be attributed to rapid and homogeneous heating of the polar reaction medium, enhanced energy transfer to the reactants, and reduced exposure of the products to prolonged thermal conditions that may otherwise promote side reactions or decomposition.

The novelty of the present study should be defined at the level of chemotype, selectivity profile, and synthetic implementation, rather than by claiming that benzimidazole-hydrazones themselves are unprecedented. Compared with recent hydrazone reports, the present compounds combine a 2-trifluoromethyl-benzimidazole core with an N1–CH_2_–CO–NH–N=CH–Ar acetohydrazone linker, whereas prior hydrazone studies have more commonly used 2-yl hydrazones lacking N1-acyl tethering or cyano-benzimidazole/benzoic hydrazide architectures [10,15]. The present series is further differentiated by a microwave-enabled route that materially improves efficiency in the key synthetic steps, and by identification of a para-hydroxy analogue (7) that provides the best cancer/normal-cell discrimination in the submitted dataset.

**Scheme 2:**
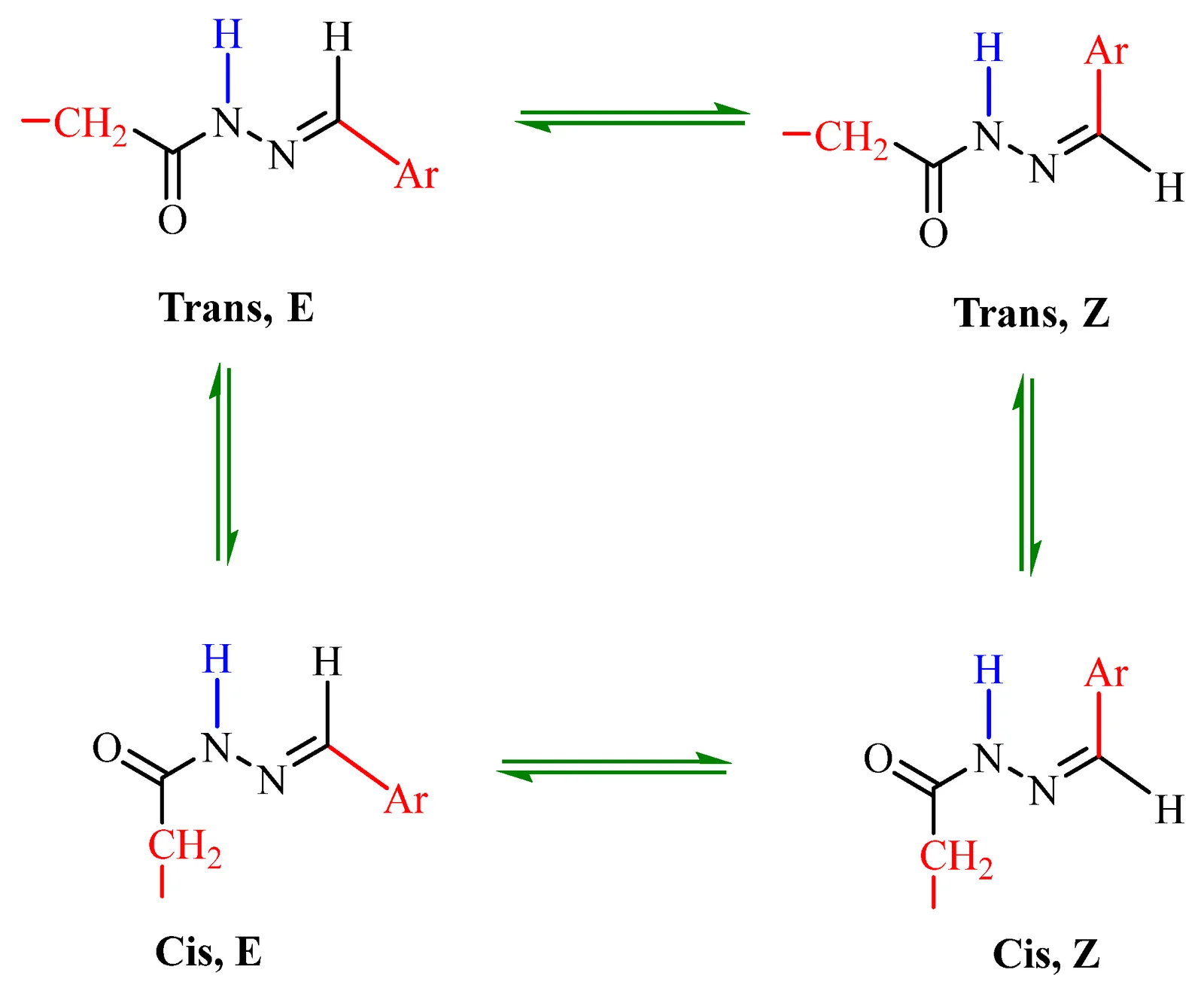
Isomeric types of hydrazone structures.

Upon examination of the ^1^H NMR data for compounds **4**–**19**, it was observed that certain protons in these compounds exhibit two distinct sets of resonances at different chemical shifts. The compounds with an arylidene–hydrazide structure occur as E/Z geometric isomers due to the C=N double bond and as cis/trans amide conformers at the CO–CH_2_ single bond (Scheme 2). Based on previous studies [16,17], compounds containing a C=N double bond preferentially adopt the E geometrical isomer in DMSO-*d*_6_, while Z isomers may be favored in less polar solvents by an intramolecular hydrogen bond.

The CO–CH_2_ resonances appeared as two distinct sets of signals, attributable to the presence of cis/trans conformers. These signals appear in the spectrum as a prominent signal at δ 5.64–5.76 ppm and a weaker signal at δ 5.21–5.31 ppm. On the other hand, two signal sets at approximately 8.50 and 8.25 ppm correspond to the imine hydrogen of the E and Z geometric isomers, respectively.

In addition, E and Z isomers are observed; two signal sets correspond to the imine hydrogen, and their chemical shift values appear as a weak signal in the range of 8.07–8.65 ppm and a strong signal in the range of 7.93–8.46 ppm, depending on the groups attached to the ortho position of the aromatic ring.

The minor additional resonances observed in the ^1^H NMR spectra are tentatively attributed to the presence of E/Z geometrical isomers and/or cis/trans amide conformers. Nevertheless, this assignment should be regarded as a plausible interpretation rather than a definitive structural attribution, since other phenomena, such as hydrazone–azo/enamine tautomerization and solvent-dependent equilibria, may also contribute to the observed signal multiplicity.

### 2.2. Structure–Activity Relationship (SAR)

A structure–activity relationship (SAR) trend can also be observed among the tested derivatives. In particular, the presence and position of substituents on the aromatic ring appear to play a key role in modulating cytotoxic activity. The para-hydroxyl substitution in compound **7** was associated with the strongest antiproliferative effect and the highest selectivity index, suggesting that this functional group may enhance interactions with intracellular targets through hydrogen-bond formation. In contrast, nitro- or methyl-substituted derivatives displayed comparatively weaker activity, indicating that both the electronic properties and steric characteristics of the substituent may influence the biological response of benzimidazole-hydrazone derivatives.

The para-hydroxyl group (compound **7**) likely provides an optimal electronic distribution and spatial orientation that facilitates productive interactions within the catalytic pocket. In contrast, the meta-hydroxyl derivative (compound **8**) exhibited weaker cytotoxic activity. Although this substituent is electron-donating, its meta position may hinder optimal positioning within the active site and reduce productive interactions. On the other hand, compound **9**, which contains an ortho-hydroxy group, showed high cytotoxic activity. One possible reason for this could potentially arise from an intramolecular hydrogen bond in its structure. This observation suggests that electronic effect alone is insufficient and must be complemented by favorable geometric factors for efficient cytotoxic activity.

In contrast, the nitro-substituted compound **4**, while cytotoxic, may exert part of its activity through redox-related or less specific mechanisms, which could explain its lower selectivity compared with compound **7**. Similarly, the methyl substituent present in compound **11** may introduce steric effects that reduce optimal target interactions, correlating with its comparatively weaker antiproliferative activity.

Importantly, the selectivity index analysis highlights a notable pharmacological distinction between the synthesized derivatives and doxorubicin. While doxorubicin remains highly potent, its similar IC_50_ values in both cancerous and non-tumorigenic cells indicate limited selectivity under the tested conditions. In contrast, compound **7** exhibited a higher selectivity index, suggesting a more favorable selectivity profile toward cancer cells.

### 2.3. Antiproliferative Activity in MCF-7 Cells

**Figure 2:**
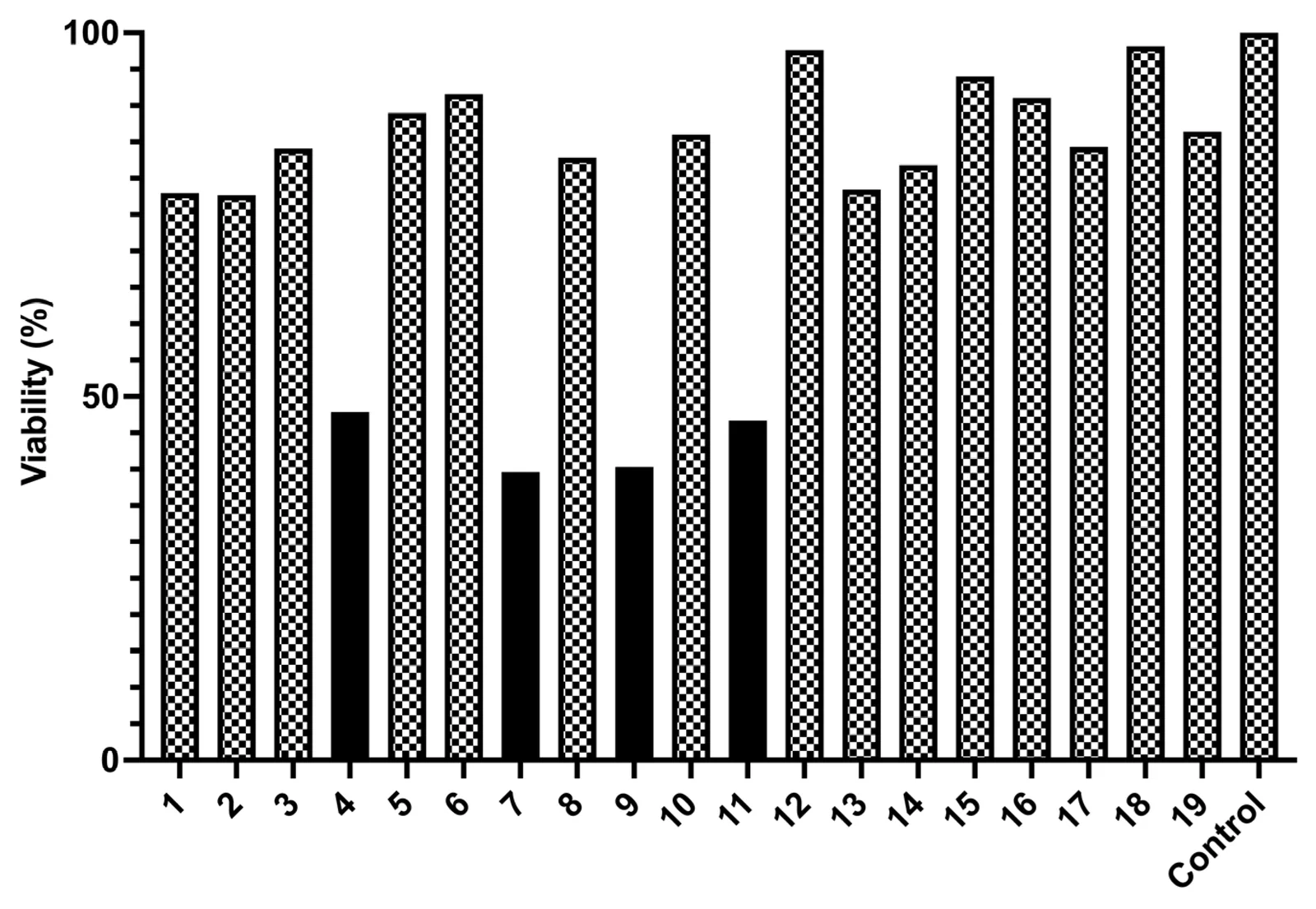
Effects of compounds **1**–**19** on MCF-7 cell viability after 48 h of treatment. Solid black bars indicate the most active compounds; patterned bars indicate the others.

Initial screening at 20 μM identified four compounds (**4**, **7**, **9**, and **11**) with notable cytotoxic effects in MCF-7 cells (Figure 2). These compounds reduced cell viability to below 50% following 48 h of exposure and were therefore selected for further dose–response analysis.

**Figure 3:**
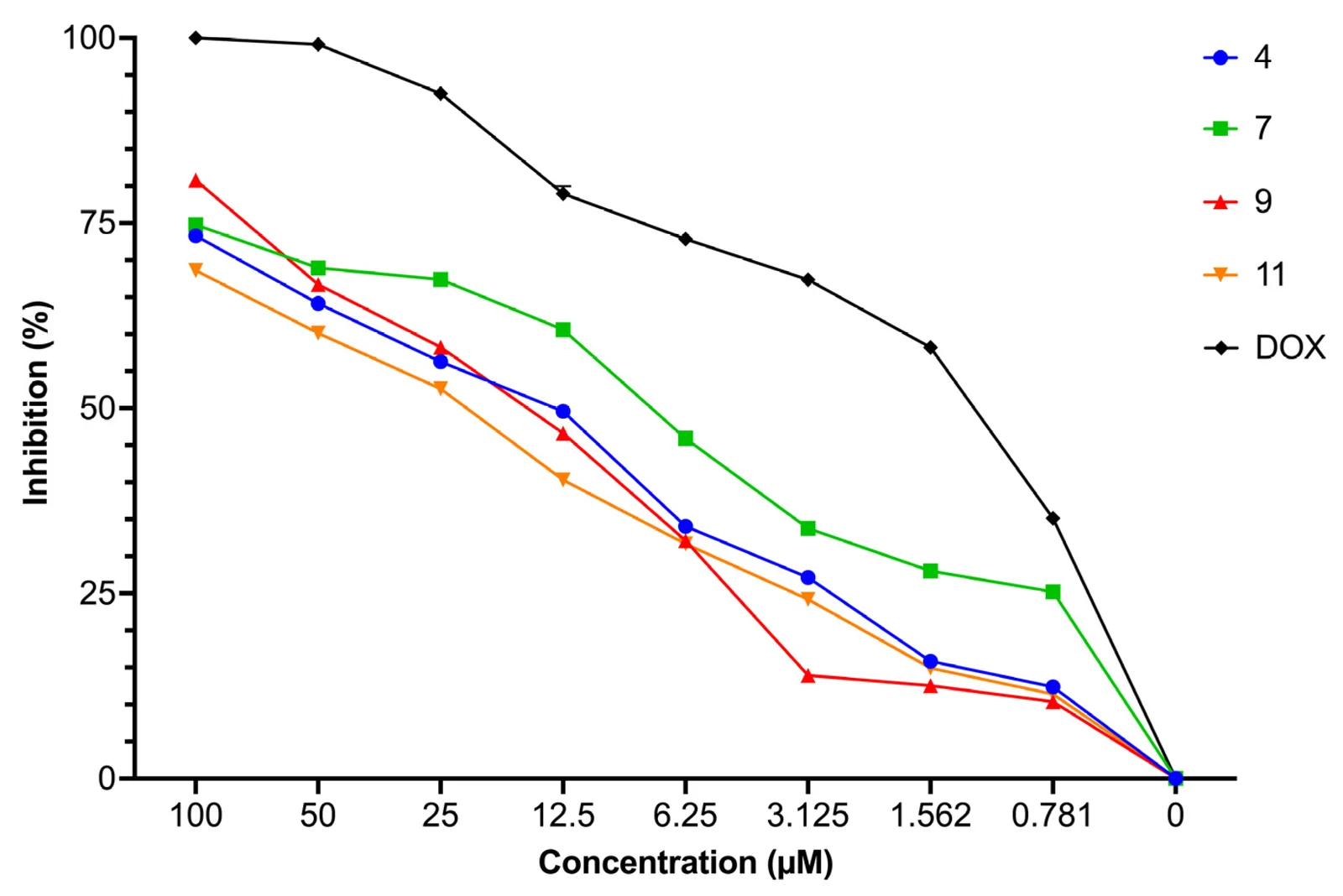
Concentration-dependent inhibition of MCF-7 cell viability by compounds **4**, **7**, **9**, and **11** and doxorubicin after 48 h.

A dose-dependent inhibition of cell viability was observed for all four derivatives in MCF-7 cells (Figure 3). The calculated IC_50_ values are summarized in Table 2.

These findings suggest that, although less potent than doxorubicin in terms of absolute IC_50_ values, compound **7** exhibits markedly improved selectivity toward cancer cells.

Among the tested compounds, compound **7** demonstrated the strongest antiproliferative activity, with an IC_50_ value of 7.5 ± 0.8 μM. This was followed by compound **4** (12.67 ± 1.5 μM), compound **9** (16.1 ± 0.7 μM), and compound **11** (22.4 ± 0.5 μM). As expected, doxorubicin exhibited potent cytotoxic activity with an IC_50_ value of 1.37 ± 0.3 μM.

To contextualize the biological profile of the present series, we compared the lead compound **7** with recent benzimidazole-based anticancer scaffolds evaluated under cell-based conditions. In our series, compound **7** displayed the best balance of potency and selectivity in MCF-7 cells (IC_50_ = 7.5 ± 0.8 μM; hTERT IC_50_ = 95 ± 1.8 μM; SI = 12.66), outperforming the recently reported CA IX-oriented benzimidazole-hydrazone derivative in the same breast-cancer model (MCF-7 IC_50_ = 25.6 ± 16.44 μM) [10] and approaching the activity range of other recent benzimidazole classes such as oxadiazole-linked benzimidazole (MCF-7 IC_50_ = 5.165 ± 0.211 μM) [18] and alkylsulfonyl benzimidazole (MCF-7 IC_50_ = 4.7 μM) [7].

Because cross-study ranking is strongly influenced by exposure time and assay platform, these comparisons should be interpreted cautiously. The present manuscript used a 48 h MTT protocol with explicit cancer-versus-normal benchmarking in hTERT cells, whereas recent comparators employed 24 h MTT, 72 h MTT, or xCELLigence real-time analysis. On that basis, we believe the most accurate positioning of compound **7** is as a selective mid-micromolar benzimidazole lead for ER-positive breast cancer cells, rather than as the most potent benzimidazole derivative reported to date. This framing also aligns with recent hydrazone literature, in which hydroxy/methoxy-substituted benzimidazole hydrazones can reach lower IC_50_ values but often under different exposure conditions and with different normal-cell controls.

**Figure 4:**
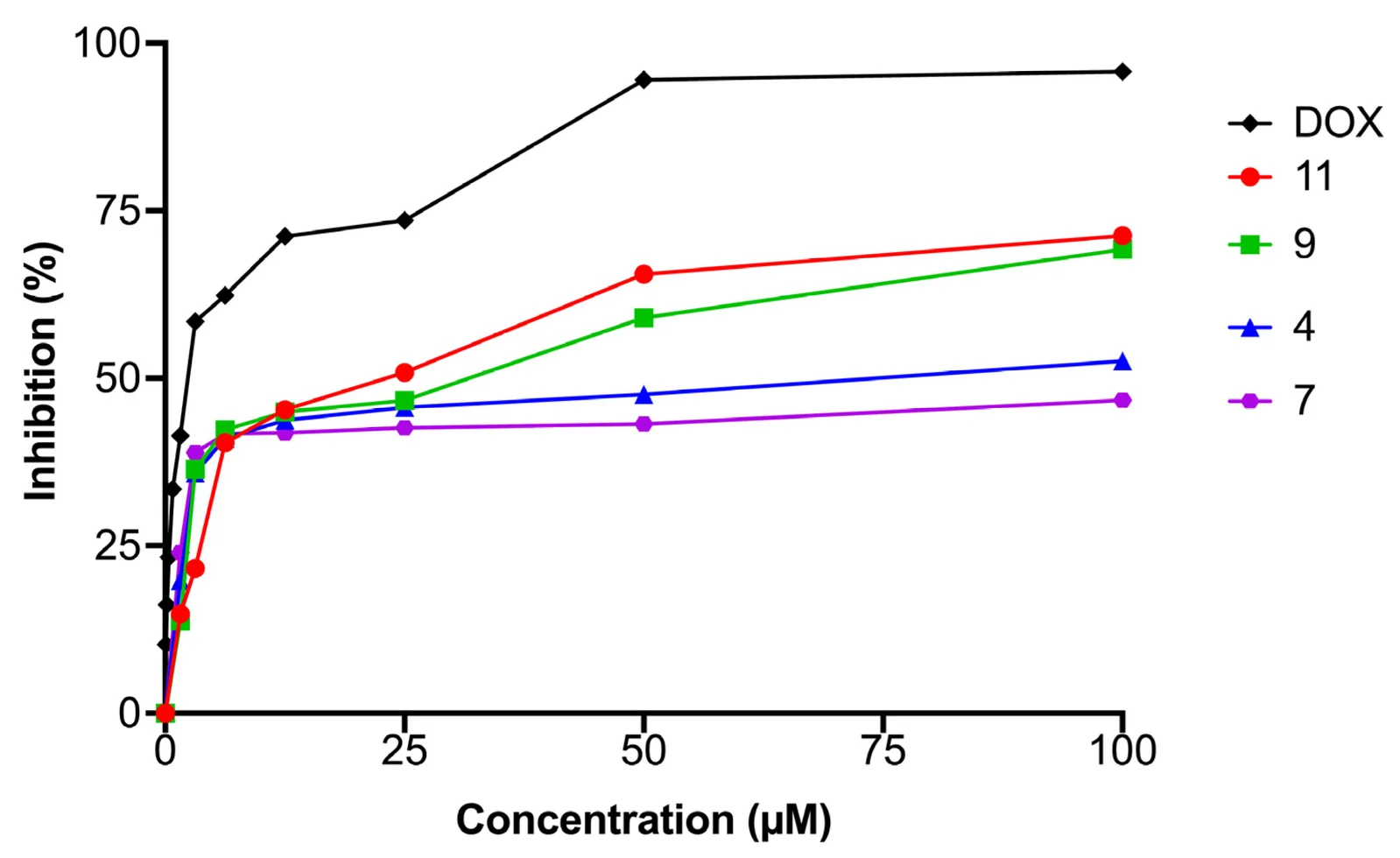
Concentration-dependent inhibition of hTERT cell viability by compounds **4**, **7**, **9**, and **11** and doxorubicin after 48 h.

To evaluate cancer selectivity, the same compounds were also tested in hTERT cells, an hTERT-immortalized, non-tumorigenic epithelial cell line commonly used as a normal-cell control in cytotoxicity studies (Figure 4). Notably, IC_50_ values in hTERT cells were substantially higher for the synthesized derivatives (ranging from approximately 80 to 95 μM) compared with those observed in MCF-7 cells. In contrast, doxorubicin showed comparable cytotoxic effects in both cancerous and non-tumorigenic cells (IC_50_ = 1.5 ± 0.3 μM in hTERT cells).

Selectivity index analysis revealed that compound **7** possessed the highest SI value (12.66), indicating preferential cytotoxicity toward breast cancer cells. Compound **4** also exhibited notable selectivity (SI = 6.49), whereas compounds **9** and **11** showed moderate selectivity. In comparison, doxorubicin demonstrated minimal selectivity (SI ≈ 1.09).

Taken together, these findings identify compound **7** as a promising candidate for further investigation. Future studies may focus on elucidating its mechanism of action, including potential effects on apoptosis induction (e.g., caspase activation), cell cycle regulation, and possible involvement of signaling pathways such as PI3K/Akt or epigenetic regulatory mechanisms, which are known to be influenced by benzimidazole-based compounds.

### 2.4. In Silico Molecular Docking Studies

To elucidate the molecular mechanisms underlying the observed antiproliferative activity against the MCF-7 breast cancer cell line and to rationalize the structure–activity relationship (SAR), comprehensive molecular docking simulations were performed. The selection of targets for the in silico molecular docking studies has been based on the biological profile of the employed MCF-7 cell line and the established pharmacophoric properties of the benzimidazole scaffold. Since MCF-7 is a well-characterized estrogen receptor-positive (ER+) breast cancer cell line, the estrogen receptor alpha (ERα) serves as the primary molecular driver of cellular proliferation. Evaluating the binding affinities against both the classical ERα antagonist state (PDB: 3ERT) and the selective estrogen receptor degrader (SERD) state (PDB: 6ZOR) is important to elucidate the antiproliferative mechanism of action [19]. Furthermore, the PI3K/Akt/mTOR signaling axis is frequently hyperactivated in breast cancer, serving as a critical survival mechanism, and benzimidazole derivatives are widely recognized in medicinal chemistry as potent mTOR kinase inhibitors (PDB: 4JSV) [20]. Finally, the epigenetic reader bromodomain-containing protein 4 (BRD4, PDB: 4J0R) was selected because it plays a pivotal role in driving oncogene expression in breast cancer, and the benzimidazole heterocyclic ring is a privileged acetyl-lysine mimetic utilized in the design of BET bromodomain inhibitors [9,21]. These targets were chosen to evaluate the multi-target inhibitory potential of the synthesized benzimidazole-hydrazone derivatives.

The calculated binding affinities for the synthesized compounds (**1**–**19**) are summarized in Table 3. Overall, the computational results demonstrate a strong correlation with the experimental in vitro cytotoxicity data. The most potent derivative identified in the MTT assays, compound **7**, consistently exhibited the most favorable and highly negative docking scores across all evaluated macromolecular targets. Specifically, compound **7** displayed exceptional binding energies of −11.24 kcal/mol and −11.77 kcal/mol for the ERα targets (3ERT and 6ZOR, respectively), −11.48 kcal/mol for the epigenetic target BRD4, and −9.47 kcal/mol for the mTOR kinase domain.

These remarkably low binding energies computationally support the hypothesis that the para-substituted hydroxyl group on the aromatic ring significantly enhances target affinity, presumably through the formation of critical and stable hydrogen-bonding networks within the active site pockets. Furthermore, other active derivatives such as compounds **4**, **9**, and **11** also yielded robust docking scores, particularly against the ERα and BRD4 targets. For instance, compound **9**, bearing an ortho-hydroxyl substitution, showed strong affinities of −10.78 kcal/mol for 3ERT and −11.28 kcal/mol for 6ZOR. As demonstrated in Table 3, the docking scores of the most potent derivative, compound **7**, are highly comparable to, and in some cases, exceed, the binding energies of the established co-crystallized native inhibitors.

### 2.5. Binding Mode and Intermolecular Interaction Analysis

**Figure 5:**
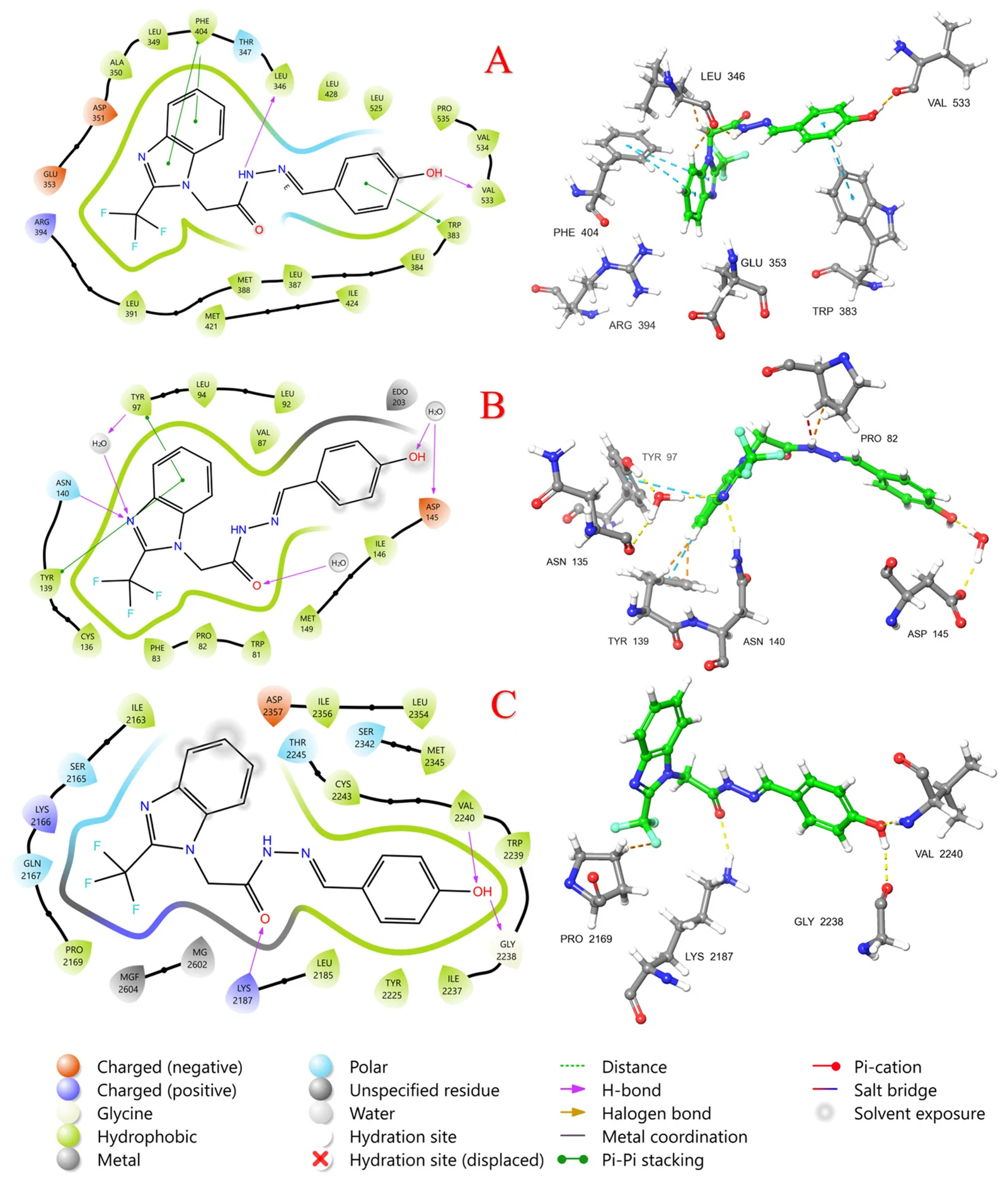
Two-dimensional (**left**) and three-dimensional (**right**) interaction diagrams of compound **7** within the active sites of (**A**) ERα (PDB: 3ERT), (**B**) BRD4 (PDB: 4J0R), and (**C**) mTOR (PDB: 4JSV).

To gain deeper structural insights into the multi-target inhibitory potential of the most potent derivative, compound **7**, detailed two-dimensional (2D) and three-dimensional (3D) intermolecular interaction analyses were conducted for the docked complexes (Figure 5). The binding poses of compound **7** within the active sites of ERα (PDB: 3ERT), BRD4 (PDB: 4J0R), and mTOR (PDB: 4JSV) revealed distinct and favorable binding orientations, driven by the versatile trifluoromethyl-benzimidazole-hydrazone scaffold.

In the ERα active site (Figure 5A), compound **7** adopts a well-accommodated conformation within the hydrophobic ligand-binding domain. The docking pose reveals that the benzimidazole core carrying the trifluoromethyl group is oriented towards the classical Glu353 and Arg394 region, occupying a deep lipophilic pocket. The stability of this complex is significantly reinforced by a robust π–π stacking interaction between the benzimidazole ring system and the aromatic side chain of Phe404. Furthermore, the hydrazone linker plays a crucial role in anchoring the molecule, as its NH group acts as a hydrogen bond donor to the backbone of Leu346. At the other end of the molecule, the para-substituted hydroxyl group (−OH) of the phenyl ring extends towards the opposite end of the cavity, establishing a key hydrogen bond with the backbone carbonyl of Val533 and a π–π stacking interaction with the Trp383 residue.

The docking analysis of compound **7** within the BRD4 epigenetic reader domain (Figure 5B) successfully corroborates its potential as an acetyl-lysine mimetic. The 2D and 3D diagrams clearly illustrate that the unsubstituted ring nitrogen of the benzimidazole ring forms a direct and critical hydrogen bond with the side chain of Asn140. This specific interaction is the universally recognized hallmark of active BRD4 bromodomain inhibitors [22]. Additionally, the benzimidazole scaffold engages in a stabilizing π–π stacking interaction with Tyr97, a key residue of the ZA loop lining the hydrophobic binding pocket [23]. The terminal para-hydroxyl group of the ligand further stabilizes the complex by participating in a hydrogen-bonding network with the side chain of Asp145 and nearby structurally conserved water molecules.

In the mTOR kinase active site (Figure 5C), compound **7** demonstrates a binding mode characteristic of potent kinase inhibitors by directly interacting with the critical hinge region [24]. The docking pose indicates that the para-hydroxyl group on the phenyl ring serves as a key pharmacophore, acting simultaneously as a hydrogen bond donor and acceptor to form stable interactions with the backbone atoms of Gly2238 and Val2240 in the hinge region [25]. Moreover, the carbonyl oxygen of the hydrazone linkage establishes a strong hydrogen bond with the positively charged side chain of Lys2187, effectively anchoring the central portion of the molecule within the ATP-binding pocket.

While the computational simulations indicate broad multi-target potential for the trifluoromethyl-benzimidazole-hydrazone scaffold, the ERα-mediated pathway is predicted to be the most probable primary mechanism of action. Since the in vitro cytotoxicity assays were exclusively performed on the estrogen receptor-positive (ER+) MCF-7 breast cancer cell line, the targeted antagonism of ERα signaling is postulated to be the predominant driver of the observed antiproliferative activity. This biological rationale is strongly corroborated by the in silico data, wherein the highest thermodynamic binding affinities for compound **7** were recorded against the ERα structural models. Consequently, although secondary synergistic apoptotic effects might be facilitated by the predicted interactions with epigenetic (BRD4) and kinase (mTOR) targets, the modulation of ERα is considered the principal mechanism underlying the cellular response in the MCF-7 model.

### 2.6. Molecular Dynamics (MD) Simulation Analysis

**Figure 6:**
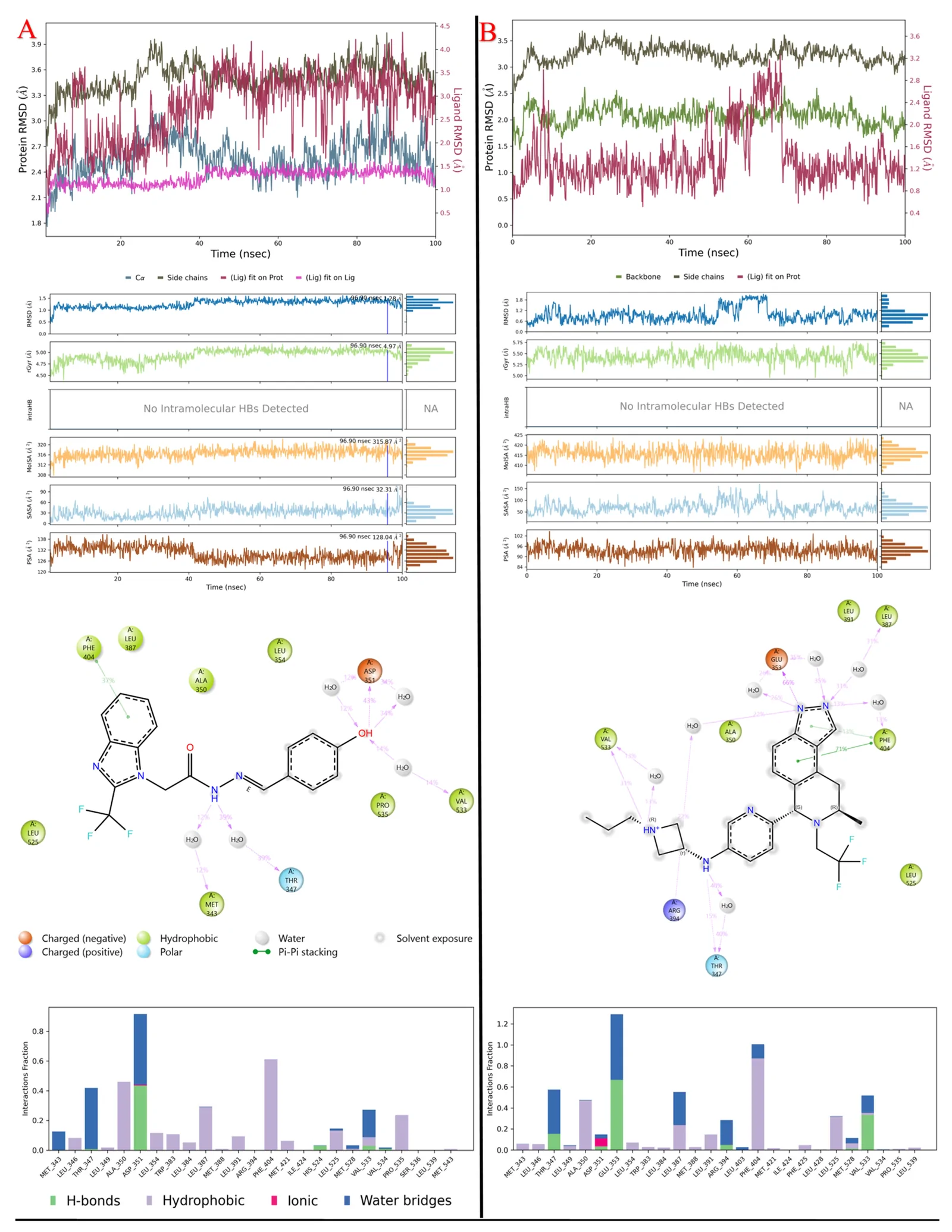
MD simulation analyses of ERα (6ZOR) in complex with compound **7** (**A**) and camizestrant (**B**) over a 100 ns trajectory. The panels sequentially depict the structural stability via RMSD profiles (**top**), ligand conformational properties including rGyr, SASA, and PSA (**middle**), and the time-dependent protein–ligand interaction fractions highlighting key binding contacts (**bottom**).

To validate the dynamic stability and structural integrity of the predicted binding modes, 100 ns MD simulations were performed for compound **7** and the reference ligand camizestrant in complex with the ERα target. The dynamic behaviors of the complexes were evaluated by analyzing the Root Mean Square Deviation (RMSD), ligand properties (rG, SASA, PSA), and the time-dependent protein–ligand interaction profiles (Figure 6).

Analysis of the Root Mean Square Deviation (RMSD) trajectories provided critical insights into the structural equilibration of the simulated systems (Figure 6, top panels). The Cα backbone RMSD for both complexes exhibited rapid convergence within the initial 5 ns, plateauing tightly between 2.1 and 2.7 Å. This fluctuation indicates that the architectural integrity of the ERα receptor is well preserved upon binding of either ligand, precluding any ligand-induced protein unfolding.

The ‘Ligand fit on Protein’ RMSD trajectory for the native reference, camizestrant (Figure 6B), demonstrated profound conformational rigidity, oscillating strictly within 1.2–1.6 Å. In contrast, compound **7** (Figure 6A) underwent an initial conformational relaxation phase, adopting a stable dynamic equilibrium at an RMSD plateau of ~3.2 Å after the 20 ns mark. Crucially, the internal structural stability of compound **7**, as evidenced by the ‘Ligand fit on Ligand’ RMSD (~1.2 Å, pink trajectory), remained intact throughout the simulation. This behavior demonstrates that, despite the ligand shifting as a feature of binding-site adaptation to optimize its position within the active site, the core molecular topology remains extremely rigid and ultimately reaches a stable secondary energetic minimum without separating from the binding pocket.

The structural compactness and solvation profiles of the ligands were further interrogated via the Radius of Gyration (rGyr) and Solvent Accessible Surface Area (SASA) metrics (Figure 6, middle panels). The rGyr values for both compound **7** (~4.9 Å) and camizestrant (~5.1 Å) exhibited negligible deviations from their respective baselines, confirming the absence of uncharacteristic steric elongation during the dynamic process. The SASA and Polar Surface Area (PSA) profiles for compound **7** corroborate that the molecule remains deeply sequestered within the lipophilic core of the ERα cavity, effectively shielded from the bulk solvent, which is a structural hallmark of high-affinity binding.

A detailed investigation of the protein–ligand interaction profile (Figure 6, bottom panels) elucidated the distinct mechanistic binding characteristics of the two molecules. The reference drug, camizestrant, sustained a classical ERα interaction network, characterized by robust, bipartite hydrogen bonding and water-bridged contacts with the pivotal residues Glu353 and Arg394, augmented by pervasive π–π aromatic packing with Phe404 (interaction fraction > 0.8). Conversely, compound **7** established a differentiated but highly durable pharmacophoric network. The primary anchoring mechanism was governed by an extensive solvent-mediated hydrogen bond network with Asp351, which was conserved for >80% of the trajectory. Furthermore, the benzimidazole-hydrazone scaffold successfully recapitulated the critical hydrophobic and π–π stacking interactions with Phe404 (fraction > 0.6) and Ala350, analogous to the reference compound. The sustained engagement with Asp351, coupled with the dense hydrophobic packing at the aromatic core, effectively compensates for the absence of direct Glu353/Arg394 contacts.

### 2.7. MM/GBSA Analysis

To rigorously quantify and compare the thermodynamic binding affinities of the simulated complexes, trajectory-averaged MM/GBSA calculations were performed. As detailed in Table 4, the calculated total binding free energy (∆*G_bind_*) for the lead candidate, compound **7** (−94.77 ± 12.94 kcal/mol), demonstrates an exceptional binding affinity that marginally surpasses that of the clinical reference standard, Camizestrant (−93.59 ± 7.90 kcal/mol). This finding thermodynamically substantiates the robust anchoring of compound **7** within the Erα (6ZOR) active site.

A detailed decomposition of the individual energetic components reveals the primary driving forces dictating the ligand recognition process. For both complexes, the binding is overwhelmingly driven by van der Waals (∆*G_vdW_*) and lipophilic (∆*G_lipo_*) interactions, which is highly consistent with the predominantly hydrophobic architecture of the Erα binding cavity. Notably, compound **7** exhibits a superior lipophilic contribution (−38.18 kcal/mol) compared with camizestrant (−35.75 kcal/mol). This thermodynamic advantage highlights the excellent structural complementarity and effective π-packing of the benzimidazole scaffold within the hydrophobic sub-pocket formed by residues such as Phe404 and Ala350.

Interestingly, while compound **7** encounters a higher coulombic penalty (∆*Gc_oulomb_* = 59.04 kcal/mol) compared with the reference ligand (44.07 kcal/mol), this energetic cost is effectively overcompensated by a highly favorable generalized Born electrostatic solvation term (∆*G_solv_GB_* = −31.73 kcal/mol vs. −19.25 kcal/mol). Furthermore, the moderately higher standard deviations observed in the electrostatic and solvation components of compound **7** perfectly corroborate the RMSD trajectory analysis; they reflect the initial conformational relaxation phase where the ligand dynamically rearranged its solvent-mediated interaction networks (particularly with Asp351) to achieve its deeply buried, thermodynamically stable energetic minimum. The comprehensive MM/GBSA profiling confirms that compound **7** achieves a highly stable, tightly packed, and thermodynamically competitive binding state relative to camizestrant, solidifying its potential as a promising targeted therapeutic agent.

### 2.8. In Silico ADMET Profiling of the Lead Compound

The pharmacokinetic parameters and drug-likeness of the lead derivative, compound **7**, were computationally evaluated to assess its translational potential in Table 5. Compound **7** exhibits an excellent drug-likeness profile with zero violations of Lipinski’s Rule of Five (RoF). It demonstrates optimal lipophilicity (logPo/w = 3.708) and highly favorable apparent Caco-2 cell permeability (PCaco = 650.85 nm/s), which collectively contribute to a perfect predictive score (100%) for human oral absorption (PHOA).

Regarding drug distribution, compound **7** displays optimal human serum albumin binding (logKhsa = 0.267) and restricted blood–brain barrier penetration (logBB = −0.841), suggesting a favorable pharmacokinetic compartmentalization that minimizes potential central nervous system (CNS) toxicity. Although the predicted hERG channel affinity (logHERG = −6.396) indicates a standard liability typical for lipophilic anticancer agents, necessitating future in vitro electrophysiological validation, the overall ADMET profile strongly supports compound **7** as a highly promising, orally bioavailable drug candidate for further development.

## 3. Materials and Methods

### 3.1. Chemistry

All materials were supplied by TCI Chemicals (Tokyo, Japan) and Sigma-Aldrich (St. Louis, MO, USA) and were used in their original state without additional purification. The reactions were monitored via thin-layer chromatography (TLC) with a layer thickness of 0.25 mm (Merck, Darmstadt, Germany), visualizing the spots under UV light at 254 nm. Melting points of the synthesized intermediates and final compounds were determined in capillary tubes using a Büchi oil-heated melting-point apparatus. The infrared spectra were obtained via the ATR (Attenuated Total Reflectance) method on a PerkinElmer Spectrum 100 FTIR Spectrometer (PerkinElmer Inc., Waltham, MA, USA). The ^1^H and ^13^C NMR spectra were acquired utilizing TMS as the internal standard on an Agilent Premium spectrometer (Agilent Technologies Inc., Santa Clara, CA, USA) operating at 400 and 100 MHz, respectively. The corresponding NMR spectra are provided in Supplementary Materials (Figures S1–S30). The elemental analyses were conducted using a Carlo-Erba 1106 CHN analyzer (Carlo Erba Instruments, Milan, Italy). A monomode CEM-Discover-II microwave device (CEM Corporation, Matthews, NC, USA) was utilized with a power setting of 300 W. All tests were conducted in 35 mL microwave process vials, with temperature regulation achieved by an infrared temperature detection sensor. The temperature was regulated and kept constant using discrete modulation of the supplied microwave power.

#### 3.1.1. Synthesis of 2-(Trifluoromethyl)-1H-benzo[d]imidazole (**1**)

o-Phenylenediamine (2.16 g, 20 mmol) was treated with trifluoroacetic acid (5 mL), and the reaction mixture was stirred at 60 °C for 5–6 h. After cooling, the reaction mixture was poured into ice-cold water and neutralized with saturated NaHCO_3_ solution, and the crude solid was filtered and crystallized from ethyl acetate. Yield (4.10 g, 92%), mp 203–205 °C (lit. 200–202 °C), white solid [26].

#### 3.1.2. Synthesis of Ethyl 2-(2-(Trifluoromethyl)-1H-benzo[d]imidazol-1-yl)acetate (**2**)

*Microwave Method:* The mixture of compound **1** (1.30 g, 0.007 mol) and dry K_2_CO_3_ (1.15 g, 0.008 mol) in dry DMF (5 mL) was irradiated with microwaves at 110 °C for 1 min (hold time) at 300 W maximum power. Ethyl bromoacetate (1.30 mL, 0.010 mol) was subsequently added to the reaction mixture, and irradiation was continued for an additional 5 min under the same conditions. Upon completion of the reaction, as monitored by TLC using EtOAc/hexane (7:3, *v*/*v*) as the eluent, the reaction mixture was poured into ice-cold water. The resulting precipitate was collected by filtration and recrystallized from ethyl acetate to afford pure compound **2**. Yield: 1.69 g, 88%, white solid.

*Conventional Method:* Dry K_2_CO_3_ (1.15 g, 0.008 mol) was added to a solution of compound **1** (1.30 g, 0.007 mol) in dry DMF (5 mL), and the mixture was stirred for 30 min under reflux conditions. Ethyl bromoacetate (1.30 mL, 0.010 mol) was subsequently added to the solution, and the reaction mixture was stirred under reflux for 24 h. Work-up and purification procedures were carried out as described above. Yield: 1.27 g, 66%, mp: 87–89 °C (lit. 88–89 °C) [14].

#### 3.1.3. Synthesis of 2-(2-(Trifluoromethyl)-1H-benzo[d]imidazol-1-yl)acetohydrazide (**3**)

*Microwave Method:* Compound **2** (1.36 g, 5.0 mmol) and excess hydrazine hydrate (2.5 mL, 50 mmol) were dissolved in anhydrous ethanol (5 mL), and the resulting mixture was subjected to microwave irradiation at 115 °C for 5 min (hold time) using a maximum power of 300 W. Upon completion of the reaction, as monitored by TLC using EtOAc/hexane (3:1, *v*/*v*) as the eluent, the resulting solid was collected by filtration and subsequently recrystallized from ethanol to afford pure compound **3**. Yield: 1.18 g, 91%, white solid.

*Conventional Method:* Compound **2** (1.36 g, 5.0 mmol) and excess hydrazine hydrate (2.5 mL, 50 mmol) were dissolved in anhydrous ethanol (15 mL), and the resulting mixture was stirred overnight. The reaction mixture was worked up, and the product was purified according to the procedures described above. Yield: 0.93 g, 72%. mp: 226–228 °C (lit. 226–228 °C) [14].

#### 3.1.4. General Procedure for the Synthesis of Hydrazone–Linked Benzimidazoles (**4**–**19**)

*Microwave Method:* The corresponding aromatic aldehyde (0.50 mmol) was dissolved in ethanol (2 mL) containing a few drops of glacial acetic acid, and the resulting solution was transferred to a sealed microwave reaction vessel. The mixture was irradiated at 120 °C for 1 min (hold time) under pressure-controlled conditions using a maximum power of 300 W. Compound **3** (0.13 g, 0.50 mmol) was subsequently added to the reaction mixture, and microwave irradiation was continued for an additional 3 min under the same conditions. Upon completion of the reaction, as monitored by TLC using EtOAc/hexane (3:1, *v*/*v*) as the eluent, the resulting solid was collected by filtration and subsequently recrystallized from ethanol to afford pure compounds **4**–**19**.

*Conventional Method:* The corresponding aromatic aldehyde (0.05 mmol) was dissolved in ethanol (15 mL) containing a few drops of glacial acetic acid, and the resulting mixture was stirred at room temperature for 30 min. Then, compound **3** (0.13 g, 0.05 mol) was added to the solution and stirring with reflux was continued for 12 h. The reaction mixture was worked up, and the resulting product was purified according to the procedures described above.

##### N′-(4-Nitrobenzylidene)-2-(2-(trifluoromethyl)-1H-benzo[d]imidazol-1-yl)acetohydrazide (**4**)

Yield: 0.18 g, (90%) (for mw method), 0.14 g, (71%) (for conv. method), mp 261–262 °C, white solid; IR (ATR): 3189 (NH), 3066 (Ar CH), 2962 (Alip CH), 1681 (C=O), 1616, 1589 (C=N), 1520, 1341 (NO_2_), 1119 (C-F). ^1^H NMR (400 MHz, DMSO-*d*_6_) δ: 12.19 (s, 1H, NH), 8.27 (d, *J* = 16 Hz, 2H, ArH), 8.35 and 8.18 (s, 1H, N=CH, E/Z geometrical isomers, E/Z ratio 20/80), 8.05–7.95 (m, 2H, ArH), 7.85–7.76 (m, 2H, ArH), 7.47–7.37 (m, 2H, ArH), 5.76 and 5.31 (s, 2H, CH_2_, *cis* and *trans* conformers, *cis*/*trans* ratio 82/18). ^13^C NMR (100 MHz, DMSO-*d*_6_) δ: 168.25, 163.39 (C=O, *cis*/*trans*), 148.47, 148.39, 145.93, 142.95 (=CH_imine_, E/Z), 140.77, 140.73, 140.69, 140.62, 140.52, 140.35, 136.83, 128.63, 128.55, 125.93, 125.85, 124.48, 124.45, 124.14, 124.04, 121.23, 121.16, 120.81, 118.12, 112.32, 112.26 (=CH Ar), 46.46, 46.24 (CH_2,_ *cis*/*trans*). Calcd. for C_17_H_12_F_3_N_5_O_3_%: C 52.18, H 3.09, N 17.90. Found %: C 52.15, H 3.06, N 17.88; mass spectrum, *m*/*z* (Irel, %): 392.2 [M+H]^+^ (100).

##### N′-(3-Nitrobenzylidene)-2-(2-(trifluoromethyl)-1H-benzo[d]imidazol-1-yl)acetohydrazide (**5**)

Yield: 0.17 g, (86%) (for mw method), 0.14 g, (70%) (for conv. method), mp 237–238 °C, white solid; IR (ATR): 3087 (NH), 3057 (Ar CH), 2945 (Alip CH), 1698 (C=O), 1616, 1600 (C=N), 1527, 1350 (NO_2_), 1118 (C-F). ^1^H NMR (400 MHz, DMSO-*d*_6_) δ: 12.13 (s, 1H, NH), 8.55 (d, *J* = 20.7 Hz, 1H, ArH), 8.37–8.20 (m, 3H, N=CH, ArH), 7.85–7.72 (m, 3H, ArH), 7.47–7.36 (m, 2H ArH), 5.76, 5.31 (s, 2H, CH_2_, *cis* and *trans* conformers, *cis*/*trans* ratio 79/21). ^13^C NMR (100 MHz, DMSO-*d*_6_) δ: 168.13, 163.29 (C=O, *cis*/*trans*), 148.73, 148.63 (=CH_imine_), 145.97, 143.09 (=CH_imine_), 140.78, 140.69, 136.83, 136.22, 136.13, 133.63, 130.88, 130.83, 125.92, 125.80, 124.86, 124.13, 124.02, 121.87, 121.84, 121.22, 121.14, 120.82, 112.34, 112.26 (=CH Ar), 46.26 (CH_2_). Calcd. for C_17_H_12_F_3_N_5_O_3_%: C 52.18, H 3.09, N 17.90. Found %: C 52.16, H 3.10, N 17.85; mass spectrum, *m*/*z* (Irel, %): 392.6 [M+H]^+^ (100).

##### N′-(2-Nitrobenzylidene)-2-(2-(trifluoromethyl)-1H-benzo[d]imidazol-1-yl)acetohydrazide (**6**)

Yield: 0.18 g, (82%) (for mw method), 0.12 g, (58%) (for conv. method), mp 222–223 °C, white solid; IR (ATR): 3216 (NH), 3080 (Ar CH), 2952 (Alip CH), 1684 (C=O), 1571 (C=N), 1520, 1341 (NO_2_), 1137 (C-F). ^1^H NMR (400 MHz, DMSO-*d*_6_) δ: 12.17 (s, 1H NH), 8.46 (s, 1H, ArH), 8.24–7.96 (m, 2H, N=CH, ArH), 7.76 (dt, *J* = 58,1, 8,0 Hz, 4H, ArH), 7.42 (dt, *J* = 26,7, 8,0 Hz, 2H, ArH), 5.70, 5.30 (s, 2H, CH_2_, *cis* and *trans* conformers, *cis*/*trans* ratio 80/20). ^13^C NMR (100 MHz, DMSO-*d*_6_) δ: 168.11, 163.26 (C=O, *cis*/*trans*), 148.56 (=CH_imine_), 144.11, 140.89, 140.77, 136.82, 134.33, 134.06, 131.34, 131.25, 129.09, 128.63, 128.51, 125.94, 125.84, 125.21, 125.04, 124.14, 124.05, 121.16, 120.81, 118.11, 112.3 (=CH Ar), 46.17 (CH_2_). Calcd. for C_17_H_12_F_3_N_5_O_3_%: C 52.18, H 3.09, N 17.90. Found %: C 52.17, H 3.11, N 17.91; mass spectrum, *m*/*z* (Irel, %): 392.2 [M+H]^+^ (100).

##### N′-(4-Hydroxybenzylidene)-2-(2-(trifluoromethyl)-1H-benzo[d]imidazol-1-yl)acetohydrazide (**7**)

Yield: 0.16 g, (88%) (for mw method), 0.13 g, (74%) (for conv. method), mp 230–231 °C, light yellow solid; IR (ATR): 3525 (OH), 3121 (NH), 3015 (Ar CH), 2964 (Alip CH), 1680 (C=O), 1602 (C=N), 1277 (C-F). ^1^H NMR (400 MHz, DMSO-*d*_6_) δ: 11.70, 11.66 (s, 1H, NH), 9.92 (s, 1H, OH), 8.12 and 7.97 (s, 1H, N=CH, E/Z geometrical isomers, E/Z ratio 20/80), 7.84–7.74 (m, 2H, ArH), 7.60–7.36 (m, 2H, ArH), 6.84–6.79 (m, 2H, ArH), 5.65 and 5.22 (s, 2H, CH_2_, *cis* and *trans* conformers, *cis*/*trans* ratio 80/20). ^13^C NMR (100 MHz, DMSO-*d*_6_) δ: 167.34 (C=O), 162.39, 160.06, 159.93 (C-O_phonel_), 148.52, 145.48 (=CH_imine_, E/Z), 140.76, 140.72, 140.69, 140.34, 136.90, 129.45, 129.31, 125.87, 125.78, 125.28, 124.09, 123.97, 121.19, 121.11, 120.84, 118.14, 116.15, 112.37 (=CH Ar), 46.11 (CH_2_). Calcd. for C_17_H_13_F_3_N_4_O_2_%: C 56.36, H 3.62, N 15.46. Found %: C 56.40, H 3.60, N 15.45; mass spectrum, *m*/*z* (Irel, %): 363.4 [M+H]^+^ (100).

##### N′-(3-Hydroxybenzylidene)-2-(2-(trifluoromethyl)-1H-benzo[d]imidazol-1-yl)acetohydrazide (**8**)

Yield: 0.17 g, (91%) (for mw method), 0.14 g, (77%) (for conv. method), mp 227–228 °C, light yellow solid; IR (ATR): 3492 (OH), 3184 (NH), 3067 (Ar CH), 2970 (Alip CH), 1678 (C=O), 1610, 1581 (C=N), 1142 (C-F). ^1^H NMR (400 MHz, DMSO-*d*_6_) δ: 11.88, 11.83 (s, 1H, NH), 9.63 (s, 1H, OH), 8.14 and 7.98 (s, 1H, N=CH, E/Z geometrical isomers, E/Z ratio 20/80), 7.88–7.73 (m, 2H, ArH), 7.49–7.35 (m, 2H ArH), 7.29–7.05 (m, 3H, ArH), 6.89–6.77 (m, 1H, ArH), 5.67, 5.25 (s, 2H, CH_2_, *cis* and *trans* conformers, *cis*/*trans* ratio 80/20). ^13^C NMR (100 MHz, DMSO-*d*_6_) δ: 167.65 (C=O), 162.79, 158.11 (C-O_phonel_), 145.47 (=CH_imine_), 140.76, 140.69, 136.91, 135.56, 135.49, 130.35, 125.90, 125.82, 124.12, 124.00, 121.13, 120.83, 119.02, 117.96, 113.39, 112.39, 112.28 (=CH Ar), 46.05 (CH_2_). Calcd. for C_17_H_13_F_3_N_4_O_2_%: C 56.36, H 3.62, N 15.46. Found %: C 56.38, H 3.58, N 15.43; mass spectrum, *m*/*z* (Irel, %): 363.2 [M+H]^+^ (100).

##### N′-(2-Hydroxybenzylidene)-2-(2-(trifluoromethyl)-1H-benzo[d]imidazol-1-yl)acetohydrazide (**9**)

Yield: 0.17 g, (93%) (for mw method), 0.13 g, (75%) (for conv. method), mp 246–247 °C white solid; IR (ATR): 3217 (OH), 3081 (NH), 2991 (Ar CH), 2955 (Alip CH), 1698 (C=O), 1608, 1576 (C=N), 1139 (C-F). ^1^H NMR (400 MHz, DMSO-*d*_6_) δ: 12.12, 11.80 (s, 1H, NH), 10.81, 10.07 (s, 1H, OH), 8.47 and 8.39 (s, 1H, N=CH, E/Z geometrical isomers, E/Z ratio 31/69), 7.85–7.24 (m, 6H, ArH), 6.89 (dt, *J* = 13.3, 7,5 Hz, 2H ArH), 5.68 and 5.28 (s, 2H, CH_2_, *cis* and *trans* conformers, *cis*/*trans* ratio 68/32). ^13^C NMR (100 MHz, DMSO-*d*_6_) δ: 167.45, 162.72 (C=O), 157.69, 156.95 (C-O_phonel_), 148.09 (=CH_imine_), 142.36, 140.76, 140.70, 136.88, 132.14, 131.92, 129.33, 126.72, 125.94, 125.78, 124.14, 123.98, 121.22, 121.11, 120.48, 119.86, 119.07, 116.79, 116.62, 112.37, 112.27 (=CH Ar), 46.13 (CH_2_). Calcd. for C_17_H_13_F_3_N_4_O_2_%: C 56.36, H 3.62, N 15.46. Found %: C 56.37, H 3.61, N 15.45; mass spectrum, *m*/*z* (Irel, %): 363.3 [M+H]^+^ (100).

##### N′-(4-Methylbenzylidene)-2-(2-(trifluoromethyl)-1H-benzo[d]imidazol-1-yl)acetohydrazide (**10**) [12]

Yield: 0.16 g, (89%) (for mw method), 0.14 g, (75%) (for conv. method), mp 245–247 °C, white solid; IR (ATR): 3192 (NH), 3027 (Ar CH), 2963 (Alip CH), 1680 (C=O), 1620, 1522 (C=N), 1119 (C-F). ^1^H NMR (400 MHz, CDCl_3_) δ: 9.73 (s, 1H, NH), 7.93 (d, *J* = 6.9 Hz, 1H, N=CH), 7.69 (s, 1H, ArH), 7.54 (d, *J* = 7.7 Hz, 2H ArH), 7.40 (d, *J* = 7.0 Hz, 3H, ArH), 7.24 (d, *J* = 8.1 Hz, 2H, ArH), 5.55 (s, 2H, CH_2_), 2.41 (s, 3H, CH_3_).

##### N′-(3-Methylbenzylidene)-2-(2-(trifluoromethyl)-1H-benzo[d]imidazol-1-yl)acetohydrazide (**11**)

Yield: 0.15 g, (81%) (for mw method), 0.13 g, (70%) (for conv. method), mp 201–202 °C, white solid; IR (ATR): 3195 (NH), 3117 (Ar CH), 2964 (Alip CH), 1682 (C=O), 1602, 1525 (C=N), 1112 (C-F). ^1^H NMR (400 MHz, DMSO-*d*_6_) δ: 11,91 and 11,86 (s, 1H, NH), 8.19 and 8.04 (s, 1H, N=CH, E/Z geometrical isomers, E/Z ratio 19/81), 7.85–7.75 (m, 2H, ArH), 7.58–7.24 (m, 6H ArH), 5.70, 5.26 (s, 2H, CH_2_, *cis* and *trans* conformers, *cis*/*trans* ratio 80/20), 2.33 (d, *J* = 10.2 Hz, 3H, CH_3_). ^13^C NMR (100 MHz, DMSO-*d*_6_) δ: 167.73 (C=O), 162.80 (C-O_phonel_), 148.31 (=CH_imine_), 145.38, 140.77, 140.36, 138.55, 136.88, 134.26, 134.19, 131.47, 131.37, 129.18, 127.96, 127.91, 125.89, 125.80, 125.03, 124.89, 124.11, 123.99, 121.21, 121.12, 120.83, 118.14, 112.36, 112.28, (=CH Ar), 46.40, 46.20 (CH_2_), 21.35 (CH_3_). Calcd. for C_18_H_15_F_3_N_4_O %: C 60.00, H 4.20, N 15.55. Found %: C 60.03, H 4.19, N 15.57; mass spectrum, *m*/*z* (Irel, %): 361.1 [M+H]^+^ (100).

##### N′-(2-Methylbenzylidene)-2-(2-(trifluoromethyl)-1H-benzo[d]imidazol-1-yl)acetohydrazide (**12**)

Yield: 0.16 g, (85%) (for mw method), 0.13 g, (69%) (for conv. method), mp 220–221 °C, white solid; IR (ATR): 3214 (NH), 3077 (Ar CH), 2940 (Alip CH), 1672 (C=O), 1600, 1566 (C=N), 1121 (C-F). ^1^H NMR (400 MHz, DMSO-*d*_6_) δ: 11,95 and 11,93 (s, 1H, NH), 8.48 and 8.34 (s, 1H, N=CH, E/Z geometrical isomers, E/Z ratio 18/82), 7.85–7.74 (m, 3H, ArH), 7.47–7.23 (m, 5H ArH), 5.68 and 5.26 (s, 2H, CH_2_, *cis* and *trans* conformers, *cis*/*trans* ratio 83/17), 2.45 (s, 3H, CH_3_). ^13^C NMR (100 MHz, DMSO-*d*_6_) δ: 167.65 (C=O), 146.86 (=CH_imine_), 144.40, 140.77, 140.71, 140.33, 137.44, 137.28, 136.89, 132.23, 131.44, 131.36, 130.44, 130.29, 127.11, 126.67, 126.53, 125.90, 125.80, 124.11, 124.00, 121.21, 121.13, 120.84, 118.14, 112.37 (=CH Ar), 46.41, 46.16 (CH_2_), 19.89, 19.49 (CH_3_). Calcd. for C_18_H_15_F_3_N_4_O %: C 60.00, H 4.20, N 15.55. Found %: C 60.04, H 4.21, N 15.53; mass spectrum, *m*/*z* (Irel, %): 361.3 [M+H]^+^ (100).

##### N′-(3-Methoxybenzylidene)-2-(2-(trifluoromethyl)-1H-benzo[d]imidazol-1-yl)acetohydrazide (**13**)

Yield: 0.17 g, (88%) (for mw method), 0.14g, (75%) (for conv. method), mp 177–178 °C, light yellow solid; IR (ATR): 3191 (NH), 3102 (Ar CH), 2966 (Alip CH), 1685 (C=O), 1607, 1580 (C=N), 1279 (C-O), 1134 (C-F). ^1^H NMR (400 MHz, DMSO-*d*_6_) δ: 11.91 (s, 1H, NH), 8.21 and 8.04 (s, 1H, N=CH, E/Z geometrical isomers, E/Z ratio 20/80), 7.88–7.75 (m, 2H, ArH), 7.45–7.34 (m, 5H ArH), 7.01 (d, *J* = 7.5 Hz, 1H, ArH), 5.70, 5.27 (s, 2H, CH_2_, *cis* and *trans* conformers, *cis*/*trans* ratio 80/20), 3.79, 3.76 (s, 3H, OCH_3_). ^13^C NMR (100 MHz, DMSO-*d*_6_) δ: 167.83, 162.89 (C=O), 161.91, 160.00 (C-O), 148.18 (=CH_imine_), 140.76, 140.36, 136.86, 135.67, 130.49, 130.41, 125.90, 125.81, 124.12, 124.01, 121.70, 121.13, 120.83, 120.49, 120.20, 118.13, 118.00, 116.88, 116.48, 112.91, 112.36, 112.31, 111.92 (=CH Ar), 55.66 (OCH_3_), 46.16 (CH_2_). Calcd. for C_18_H_15_F_3_N_4_O_2_%: C 57.45, H 4.02, N 14.89. Found %: C 57.41, H 4.00, N 14.88; mass spectrum, *m*/*z* (Irel, %): 377.1 [M+H]^+^ (100).

##### N′-(2-Methoxybenzylidene)-2-(2-(trifluoromethyl)-1H-benzo[d]imidazol-1-yl)acetohydrazide (**14**)

Yield: 0.17 g, (91%) (for mw method), 0.14 g, (76%) (for conv. method), mp 198–199 °C, light yellow solid; IR (ATR): 3193 (NH), 3017 (Ar CH), 2912 (Alip CH), 1673 (C=O), 1602, 1578 (C=N), 1239 (C-O), 1115 (C-F). ^1^H NMR (400 MHz, DMSO-*d*_6_) δ: 11.92, 11.84 (s, 1H, NH), 8.58 and 8.41 (s, 1H, N=CH, E/Z geometrical isomers, E/Z ratio 19/81), 7.94–7.75 (m, 3H, ArH), 7.45–7.00 (m, 5H ArH), 5.68, 5.23 (s, 2H, CH_2_, *cis* and *trans* conformers, *cis*/*trans* ratio 80/20), 3.85 (d, *J* =1.5 Hz, 3H, OCH_3_). ^13^C NMR (100 MHz, DMSO-*d*_6_) δ: 167.64, 162.59 (C=O), 158.26, 158.20 (C-O), 143.76 (=CH_imine_), 140.86, 140.77, 140.73, 140.71, 140.36, 136.88, 132.33, 132.18, 126.17, 125.97, 125.91, 125.79, 124.11, 123.98, 122.26, 122.17, 121.23, 121.21, 121.16, 121.12, 120.83, 118.14, 112.37, 112.30, 112.27 (=CH Ar), 56.18 (OCH_3_), 46.18 (CH_2_). Calcd. for C_18_H_15_F_3_N_4_O_2_%: C 57.45, H 4.02, N 14.89. Found %: C 57.46, H 4.02, N 14.87; mass spectrum, *m*/*z* (Irel, %): 377.2 [M+H]^+^ (100).

##### N′-(Pyridin-4-ylmethylene)-2-(2-(trifluoromethyl)-1H-benzo[d]imidazol-1-yl)acetohydrazide (**15**)

Yield: 0.15 g, (84%) (for mw method), 0.13 g, (76%) (for conv. method), mp 250–251 °C, brown solid; IR (ATR): 3089 (NH), 2935 (Alip CH), 1709 (C=O), 1602, 1528 (C=N), 1269 (C-F). ^1^H NMR (400 MHz, DMSO-*d*_6_) δ: 12.20, 12.17 (s, 1H, NH), 8.66 (d, *J* = 5.3 Hz, 2H, Py N=CH), 8.24 and 8.06 (s, 1H, N=CH, E/Z geometrical isomers, E/Z ratio 16/84), 7.85–7.37 (m, 4H, ArH), 7.42 (dt, *J* = 24.6, 7.4 Hz, 2H, ArH), 5.74, 5.30 (s, 2H, CH_2_, *cis* and *trans* conformers, *cis*/*trans* ratio 82/18). ^13^C NMR (100 MHz, DMSO-*d*_6_) δ: 168.28, 163.37 (C=O, *cis*/*trans*), 150.71 (=CH_imine_, E/Z), 145.99 (C=N_Ar_), 142.83 (=CH_imine_, E/Z), 141.41, 140.77, 140.73, 140.69, 140.35, 136.84, 125.85, 124.05, 121.48, 121.16, 120.81, 118.12, 112.34 (=CH Ar), 46.22 (CH_2_). Calcd. for C_16_H_12_F_3_N_5_O %: C 55.33, H 3.48, N 20.17. Found %: C 55.30, H 3.46, N 20.17; mass spectrum, *m*/*z* (Irel, %): 348.2 [M+H]^+^ (100).

##### N′-(Pyridin-3-ylmethylene)-2-(2-(trifluoromethyl)-1H-benzo[d]imidazol-1-yl)acetohydrazide (**16**)

Yield: 0.15 g, (89%) (for mw method), 0.14 g, (80%) (for conv. method), mp 222–223 °C, light brown solid; IR (ATR): 3067 (NH), 2952 (Alip CH), 1699 (C=O), 1613, 1525 (C=N), 1117 (C-F). ^1^H NMR (400 MHz, DMSO-*d*_6_) δ: 12.05 (s, 1H, NH), 9.05–8.84 (m, 1H, N=CH), 8.61–8.58 (t, *J* = 5.9 Hz, 1H, Py C=N), 8.29–8.08 (m, 2H, ArH), 7.85–7.76 (m, 2H, ArH), 7.49–7.36 (m, 2H, ArH), 5.73 and 5.29 (s, 2H, CH_2_, *cis* and *trans* conformers, *cis*/*trans* ratio 81/19). ^13^C NMR (100 MHz, DMSO-*d*_6_) δ: 168.03, 163.07 (C=O, *cis*/*trans*), 151.19, 149.35 (=CH_imine_, E/Z), 149.13, 145.68 (C=N_Ar_), 142.47, 140.77, 140.69, 140.39, 136.84, 134.20, 134.01, 130.27, 130.23, 125.82, 124.35, 124.03, 121.21, 121.14, 120.82, 118.12, 112.32 (=CH Ar), 46.23 (CH_2_). Calcd. for C_16_H_12_F_3_N_5_O %: C 55.33, H 3.48, N 20.17. Found %: C 55.35, H 3.47, N 20.14; mass spectrum, *m*/*z* (Irel, %): 348.0 [M+H]^+^ (100).

##### N′-(4-(Dimethylamino)benzylidene)-2-(2-(trifluoromethyl)-1H-benzo[d]imidazol-1-yl)acetohydrazide (**17**)

Yield: 0.18 g, (93%) (for mw method), 0.15 g, (75%) (for conv. method), mp 243–245 °C, white solid; IR (ATR): 3182 (NH), 3092 (Ar CH), 2956 (Alip CH), 1675 (C=O), 1603, 1555 (C=N), 1141 (C-F). ^1^H NMR (400 MHz, DMSO-*d*_6_) δ: 11.59 (d, *J* = 9.7 Hz, 1H, NH), 8.07 and 7.93 (s, 1H, N=CH, E/Z geometrical isomers, E/Z ratio 22/78), 7.81 (dd, *J* = 15.0, 8.1 Hz, 2H, ArH), 7.56–7.34 (m, 4H, ArH), 6.74 (d, *J* = 8.9 Hz, 2H), 5.64 and 5.21 (s, 2H, CH_2_, *cis* and *trans* conformers, *cis*/*trans* ratio 79/21), 2.95 (s, 6H, CH_3_). ^13^C NMR (100 MHz, DMSO-*d*_6_) δ: 167.06, 162.09 (C=O, *cis*/*trans*), 152.09, 152.00, 148.98, 145.99 (=CH_imine_, E/Z), 140.76, 140.71, 136.93, 129.00, 128.84, 125.85, 125.76, 124.07, 123.95, 121.54, 121.48, 121.19, 121.10, 120.85, 118.15, 112.41, 112.29, 112.19, (=CH Ar), 46.08 (CH_2_), 40.58 (CH_3_). Calcd. for C_19_H_18_F_3_N_5_O %: C 58.61, H 4.66, N 17.99. Found %: C 58.60, H 4.64, N 17.97; mass spectrum, *m*/*z* (Irel, %): 390.2 [M+H]^+^ (100).

##### N′-(4-Chlorobenzylidene)-2-(2-(trifluoromethyl)-1H-benzo[d]imidazol-1-yl)acetohydrazide (**18**)

Yield: 0.16 g, (84%) (for mw method), 0.15 g, (77%) (for conv. method), mp 225–227 °C, white solid; IR (ATR): 3196 (NH), 3099 (Ar CH), 2965 (Alip CH), 1694 (C=O), 1616, 1594 (C=N), 1121 (C-F), 746 (C-Cl). ^1^H NMR (400 MHz, DMSO-*d*_6_) δ: 11.95 (s, 1H, NH), 8.23 and 8.07 (s, 1H, N=CH, E/Z geometrical isomers, E/Z ratio 19/81), 7.85–7.71 (m, 4H, ArH), 7.53–7.36 (m, 4H, ArH), 5.70 and 5.27 (s, 2H, CH_2_, *cis* and *trans* conformers, *cis*/*trans* ratio 81/19). ^13^C NMR (100 MHz, DMSO-*d*_6_) δ: 167.87, 162.96 (C=O, *cis*/*trans*), 147.00, 143.96 (=CH_imine_, E/Z), 140.77, 140.35, 136.87, 135.22, 135.09, 133.28, 133.23, 129.37, 129.30, 129.23, 125.91, 125.81, 124.12, 124.01, 121.21, 121.14, 120.82, 118.12, 112.35 (=CH Ar), 46.41, 46.18 (CH_2_). Calcd. for C_17_H_12_ClF_3_N_4_O %: C 53.63, H 3.18, N 14.71. Found %: C 53.59, H 3.17, N 14.70; mass spectrum, *m*/*z* (Irel, %): 381.3 [M+H]^+^ (100).

##### N′-(4-Bromobenzylidene)-2-(2-(trifluoromethyl)-1H-benzo[d]imidazol-1-yl)acetohydrazide (**19**)

Yield: 0.19 g, (88%) (for mw method), 0.15 g, (70%) (for conv. method), mp 201–202 °C, light brown solid; IR (ATR): 3200 (NH), 3072 (Ar CH), 2957 (Alip CH), 1691 (C=O), 1616, 1590 (C=N), 1104 (C-F). ^1^H NMR (400 MHz, DMSO-*d*_6_) δ: 11.96 (s, 1H, NH), 8.21 and 8.05 (s, 1H, N=CH, E/Z geometrical isomers, E/Z ratio 19/81), 7.85–7.72 (m, 4H, ArH), 7.66–7.64 (d, *J* = 8.0 Hz, 2H, ArH), 7.46–7.36 (m, 2H, ArH), 5.70 and 5.27 (s, 2H, CH_2_, *cis* and *trans* conformers, *cis*/*trans* ratio 81/19). ^13^C NMR (100 MHz, DMSO-*d*_6_) δ: 167.87, 162.96 (C=O, *cis*/*trans*), 147.08, 144.07 (=CH_imine_, E/Z), 140.77, 140.35, 136.86, 136.75, 133.62, 133.56, 132.30, 132.28, 129.52, 129.45, 125.90, 125.81, 124.01, 123.90, 121.21, 121.13, 120.82, 118.12, 112.35, 112.27 (=CH Ar), 46.19 (CH_2_). Calcd. for C_17_H_12_BrF_3_N_4_O %: C 48.02, H 2.84, N 13.18. Found %: C 48.00, H 2.82, N 13.16.; mass spectrum, *m*/*z* (Irel, %): 425.1 [M+H]^+^ (100), 426.2 [M+2H]^+^ (95).

### 3.2. Cell Proliferation Assay

The antiproliferative activities of the synthesized benzimidazole-hydrazone derivatives were evaluated using the human breast adenocarcinoma cell line MCF-7 (ER^+^ subtype). To assess cancer selectivity, immortalized human telomerase reverse transcriptase-transfected epithelial cells (hTERT) were used as a non-tumorigenic control model.

MCF-7 and hTERT cells were cultured in Dulbecco’s Modified Eagle Medium (DMEM) supplemented with 10% fetal bovine serum (FBS) and 1% penicillin–streptomycin and maintained at 37 °C in a humidified incubator containing 5% CO_2_.

All benzimidazole derivatives (compounds **1**–**19**) were dissolved in DMSO to prepare 100 mM stock solutions. Working solutions were freshly prepared by diluting the stock solutions in culture medium immediately prior to treatment. The final concentration of DMSO in all experimental conditions did not exceed 0.1% (*v*/*v*), and vehicle controls were included accordingly.

For the preliminary screening, MCF-7 cells were seeded into 96-well plates at a density of 5 × 10^3^ cells per well and allowed to attach for 24 h. The cells were then treated with each compound at a fixed concentration of 20 μM for 48 h. Cell viability was determined using the MTT assay. Following incubation, MTT solution (5 mg/mL in PBS) was added to each well and incubated for 3–4 h. The resulting formazan crystals were subsequently dissolved in DMSO, and absorbance was measured at 570 nm using a microplate reader.

Based on the initial screening results (Figure 1), compounds **4**, **7**, **9**, and **11**, which reduced cell viability below 50% at 20 μM, were selected for detailed dose–response analysis.

For IC_50_ determination, MCF-7 cells were treated with increasing concentrations of the selected compounds (0.315–100 μM) for 48 h. Doxorubicin was used as a positive control under identical conditions. Parallel experiments were also conducted in hTERT cells using the same concentration range.

Cell viability percentages were normalized to vehicle controls. Nonlinear regression analysis was performed using GraphPad Prism (Version 11.0) software to generate dose–response curves and calculate IC_50_ values (log[inhibitor] vs. normalized response–variable slope model).

The Selectivity Index (SI) was calculated using the following formula:

SI = IC_50_ (hTERT)/IC_50_ (MCF-7)

All experiments were performed in triplicate and repeated in at least three independent experiments. Results are presented as mean ± standard deviation (SD). Statistical comparisons between compounds and doxorubicin were performed using one-way ANOVA followed by Tukey’s post-hoc test (GraphPad Prism, Version 11), with *p* < 0.05 considered statistically significant.

### 3.3. Molecular Docking Studies

To investigate the potential binding modes and multi-target interaction profiles of the synthesized derivatives, in silico molecular docking simulations were performed utilizing the Schrödinger Maestro molecular modeling suite [27]. The computational protocol targeted the following crystal structures retrieved from the RCSB Protein Data Bank (PDB): human estrogen receptor alpha (ERα) ligand-binding domain in complex with 4-hydroxytamoxifen (PDB ID: 3ERT) and Oestrogen receptor ligand binding domain (PDB ID: 6ZOR), the mammalian target of rapamycin (mTOR) kinase domain (PDB ID: 4JSV), and the bromodomain-containing protein 4 (BRD4) epigenetic reader (PDB ID: 4J0R) [25,28,29,30].

#### 3.3.1. Protein and Ligand Preparation

Prior to docking, the selected crystallographic protein structures were rigorously prepared using the Protein Preparation Wizard module [31]. The preparation process involved the removal of co-crystallized water molecules situated beyond 5.0 Å of the native ligands, assignment of bond orders, and the addition of missing hydrogen atoms. The protonation and tautomeric states of the amino acid residues were predicted at a physiological pH of 7.4 ± 0.2 using Epik. Finally, the hydrogen bond network was optimized, and a restrained energy minimization was conducted using the OPLS3e force field until the root-mean-square deviation (RMSD) of the heavy atoms converged to 0.30 Å [32].

The 2D chemical structures of the synthesized compounds were drawn and subsequently converted into 3D geometries using the LigPrep module. For each ligand, the lowest energy conformers were generated, and the possible ionization states at pH 7.4 ± 2.0 were calculated using Epik. The ligand geometries were then energetically minimized utilizing the OPLS3e force field.

#### 3.3.2. Induced Fit Docking (IFD) Protocol

To accurately model the binding poses by accounting for the conformational plasticity of the target binding sites, the Induced Fit Docking (IFD) protocol was employed [33,34]. This method integrates the docking capabilities of Glide with the protein structure refinement features of Prime to simulate the mutual conformational adaptations between the ligand and the receptor. For each target protein, the binding site was defined by the centroid of the respective co-crystallized native ligand. The prepared ligands were initially docked into the rigid receptor using Glide with a softened potential. The van der Waals (vdW) radii scaling factor was set to 0.50 for both the receptor and the ligand non-polar atoms to temporarily accommodate steric clashes, generating an initial ensemble of poses (up to 20 poses per ligand). For each generated docking pose, the side chains of the receptor residues located within a 5.0 Å radius of the ligand were subjected to conformational refinement and energy minimization using the Prime module. This step induces the necessary active site rearrangements to fit the specific ligand topology. Finally, the ligands were re-docked into their respective Prime-refined receptor conformations using the Glide Extra Precision (XP) mode. The final poses were evaluated and ranked based on Docking Score and interaction profiles of the ligand-receptor complexes.

For each target protein, the active binding site was defined by generating a receptor grid box centered directly on the centroid of the respective co-crystallized native ligand. An outer box dimension of 15 Å was utilized for all target structures. This enclosing volume was selected to allow comprehensive conformational sampling of both the ligands and the flexible active site residues during the IFD process.

#### 3.3.3. Molecular Dynamics Simulations and MM/GBSA Calculations

To evaluate the dynamic stability, conformational behavior, and structural integrity of the predicted binding modes, molecular dynamics (MD) simulations were performed for compound **7** and the reference ligand (camizestrant) in complex with the ERα SERD state (PDB: 6ZOR). All MD simulations were carried out using the Desmond module of the Schrödinger software suite (version 2018-4) [35,36].

Prior to the production runs, the protein–ligand complexes were prepared using the System Builder module. Each complex was solvated in an orthorhombic simulation box utilizing the TIP3P explicit water model, with a minimum buffer distance of 10 Å between the protein surface and the box boundaries. To mimic physiological conditions, the systems were neutralized, and a salt concentration of 0.15 M NaCl was added. The OPLS4 force field was employed to define the topological and energetic parameters of both the protein and the ligands. Following system construction, the default Desmond relaxation protocol was applied to equilibrate the systems. The production MD simulations were executed for 100 ns under the NPT ensemble. The temperature was strictly maintained at 310 K using the Nosé–Hoover chain thermostat to simulate human physiological temperature, while the pressure was kept constant at 1.01325 bar utilizing the Martyna–Tobias–Klein barostat. The simulation trajectories were recorded at regular intervals for subsequent evaluations. Finally, the generated trajectories were analyzed using the Simulation Interaction Diagram (SID) tool to extract critical stability and interaction metrics, including the Root Mean Square Deviation (RMSD), Root Mean Square Fluctuation (RMSF), Radius of Gyration (Rg), Solvent Accessible Surface Area (SASA), and time-dependent protein–ligand contact profiles.

Furthermore, to provide a more rigorous thermodynamic evaluation of the binding affinities that accounts for dynamic conformational fluctuations, molecular mechanics generalized Born surface area (MM/GBSA) calculations were performed directly on the MD trajectories [37]. For this purpose, representative conformational snapshots were extracted from the 100 ns production trajectory at regular 1 ns intervals, yielding a total of 100 frames for each protein–ligand complex. The binding free energies ($\Delta G_{bind}$) of these extracted frames were computed using the Prime MM-GBSA module of the Schrödinger suite, employing the VSGB solvation model. The final binding affinity for each complex was reported as the arithmetic mean of the free energies calculated across these 100 frames, providing a highly reliable and dynamically averaged energetic profile.

#### 3.3.4. In Silico ADMET and Drug-Likeness Prediction

The pharmacokinetic, physicochemical, and preliminary toxicity (ADMET) profiles of the synthesized derivatives were computationally evaluated utilizing the QikProp module of the Schrödinger software suite (version 2018-4) [38].

## 4. Conclusions

In this study, a novel series of benzimidazole-hydrazone derivatives incorporating a trifluoromethyl moiety were successfully synthesized using both microwave-assisted and conventional methods, with the microwave approach demonstrating superior efficiency in terms of reaction yield and time. The present study demonstrates that structural modification of the benzimidazole scaffold through hydrazone linkage and aromatic substitution significantly influences antiproliferative activity and cancer selectivity.

Compound **7**, bearing a para-hydroxyl substituent, exhibited the most favorable biological profile, combining relatively low IC_50_ values in MCF-7 cells with higher IC_50_ values in hTERT cells. The enhanced selectivity of compound **7** may be associated with the presence of the para-hydroxyl group, which is capable of forming hydrogen-bond interactions with intracellular protein targets involved in tumor cell survival. Such interactions may stabilize ligand binding within enzymatic or signaling domains, thereby enhancing inhibitory activity. In this study, MCF-7 was chosen as an ER-positive model to relate the cytotoxicity results to the docking studies performed on the estrogen receptor (3ERT, 6ZOR). A limitation of the current work is that the evaluation was restricted to this single breast cancer cell line; extending it to triple-negative (MDA-MB-231, MDA-MB-468) and other ER-positive (T47D) lines is planned as a follow-up study to clarify whether the selectivity observed here extends across breast cancer subtypes.

Despite the promising antiproliferative activity and selectivity observed for compound **7**, several limitations of the present study should be acknowledged. The biological evaluation was restricted to in vitro cytotoxicity assays using a single ER-positive breast cancer cell line and one non-tumorigenic cell model, which limits the generalizability of the findings across different breast cancer subtypes. Moreover, although the molecular docking results suggest possible interactions with hormonal, epigenetic, and kinase-related targets, these predictions were not supported by experimental mechanistic validation and should therefore be interpreted as hypothesis-generating rather than confirmatory. The absence of in vivo efficacy, pharmacokinetic, and toxicity studies also prevents definitive conclusions regarding the therapeutic potential of these compounds. Future studies should include additional ER-positive and triple-negative breast cancer cell lines, target-based and pathway-specific mechanistic assays, and appropriate in vivo models to validate the proposed multi-target activity and establish the efficacy and safety profile of compound **7**.

The ability of compound **7** to adopt favorable predicted binding poses in structurally diverse protein pockets suggests that the trifluoromethyl-benzimidazole-hydrazone scaffold may provide a versatile framework for further optimization. In silico results suggest a hypothesized synergistic, multi-target inhibitory mechanism encompassing hormonal, epigenetic, and kinase-mediated signaling pathways as a potential explanation for the in vitro antiproliferative effects in MCF-7 cells. The ability of Compound **7** to form specific, target-tailored hydrogen bonds and hydrophobic contacts across entirely different protein architectures (hormonal, epigenetic, and kinase) underscores the exceptional pharmacological versatility of the para-hydroxy-phenyl substituted benzimidazole-hydrazone framework. The interaction profiles visually and structurally provide an acceptable rationale for the in vitro IC_50_ results and the high docking scores obtained in the in silico screening. The MD simulation data substantiate that Compound **7** occupies the ERα active site with a high degree of structural stability and conformational complementarity. The transition to a stable binding state, driven by specific solvent-mediated interactions and conserved hydrophobic contacts, supports the dynamic stability of the proposed docking pose and underscores the potential of this structural framework for further investigation. However, further biochemical target engagement assays are warranted to definitively confirm this multi-target mechanism.

## Figures and Tables

**Table 1:** Comparison of reaction times and isolated yields obtained under microwave-assisted and conventional conditions.

Compound	Reaction Time (MW)	Yield (%) (MW)	Reaction Time (Conventional)	Yield (%) (Conventional)	Yield Improvement (% Points)
**2**	6 min	88	24 h	66	22
**3**	5 min	91	14 h	72	19
**4**	4 min	90	12 h	71	19
**5**	4 min	86	12 h	70	16
**6**	4 min	82	12 h	58	24
**7**	4 min	88	12 h	74	14
**8**	4 min	91	12 h	77	14
**9**	4 min	93	12 h	75	18
**10**	4 min	89	12 h	75	14
**11**	4 min	81	12 h	70	11
**12**	4 min	85	12 h	69	16
**13**	4 min	88	12 h	75	13
**14**	4 min	91	12 h	76	15
**15**	4 min	84	12 h	76	8
**16**	4 min	89	12 h	80	9
**17**	4 min	93	12 h	75	18
**18**	4 min	84	12 h	77	7
**19**	4 min	88	12 h	70	18

**Table 2:** IC_50_ values of compounds against MCF-7 breast cancer cells and hTERT cells, together with their selectivity indices.

Compounds/ Cell Line	MCF-7 IC_50_ (μM)	hTERT IC_50_ (μM)	Selectivity Index (SI)
**4**	12.67 ± 1.5	82.3 ± 1.4	6.495
**7**	7.5 ± 0.8	95 ± 1.8	12.660
**9**	16.1 ± 0.7	80.34 ± 1	4.990
**11**	22.4 ± 0.5	80.1 ± 1.2	3.575
Dox *	1.37 ± 0.3	1.5 ± 0.3	1.094

* Doxorubicin was included as a reference compound.

**Table 3:** Docking scores of synthesized compounds against selected targets in kcal/mol. Reference ligands (Ref. Lig) are the respective co-crystallized native ligands extracted from the PDB structures.

Comp/Pdb Id	4JSV	4J0R	6ZOR	3ERT
**1**	−6.97	−7.61	−9.63	−8.64
**2**	−5.94	−8.58	−9.28	−8.65
**3**	−6.07	−7.98	−8.35	−8.40
**4**	−6.51	−9.38	−9.51	−9.22
**5**	−6.82	−10.49	−9.89	−9.57
**6**	−7.53	−8.39	−10.81	−10.28
**7**	−9.47	−11.48	−11.77	−11.24
**8**	−7.22	−10.63	−11.10	−10.29
**9**	−7.91	−9.74	−11.28	−10.78
**10**	−7.81	−9.46	−10.34	−9.95
**11**	−7.81	−8.98	−11.01	−10.98
**12**	−7.66	−9.28	−11.24	−9.78
**13**	−7.70	−9.95	−10.21	−10.14
**14**	−7.37	−9.48	−10.72	−10.64
**15**	−9.63	−9.56	−9.27	−9.76
**16**	−8.31	−10.17	−10.71	−9.72
**17**	−7.02	−8.99	−10.73	−11.32
**18**	−7.88	−9.16	−9.98	−10.86
**19**	−7.57	−8.95	−10.60	−9.72
Ref. Lig	−10.25	−11.41	−11.69	−11.07

**Table 4:** Trajectory-averaged MM/GBSA binding free energies and corresponding energy components (kcal/mol) of compound **7** and Camizestrant over 100 ns MD simulations.

	Compound 7			Camizestrant (Referans Ligand)		
Energy Component	Mean ± SD	Min	Max	Mean ± SD	Min	Max
∆*G_bind_*	−94.77 ± 12.94	−125.44	−56.81	−93.59 ± 7.90	−116.58	−75.61
∆*G_vdW_*	−86.96 ± 10.17	−105.95	−53.69	−86.44 ± 6.37	−102.74	−72.82
∆*Gc_oulomb_*	59.04 ± 45.52	−62.09	140.19	44.07 ± 38.98	−57.15	117.61
∆*G_solv_GB_*	−31.73 ± 43.16	−113.92	74.57	−19.25 ± 37.72	−89.98	82.30
∆*G_Hbond_*	−1.46 ± 0.92	−4.95	0.45	−1.55 ± 0.76	−3.43	0.33
∆*G_lipo_*	−38.18 ± 4.12	−46.94	−24.93	−35.75 ± 2.57	−42.42	−30.73
∆*G_pack_*	−1.39 ± 0.80	−3.32	0.12	−1.09 ± 0.60	−2.53	0.00

∆*G_bind_*: Total binding free energy; ∆*G_vdW_*: Van der Waals energy; ∆*Gc_oulomb_*: Coulombic energy; ∆*G_solv_GB_*: Generalized Born electrostatic solvation energy; ∆*G_Hbond_*: Hydrogen-bonding correction; ∆*G_lipo_*: Lipophilic energy; ∆*G_pack_*: π–π packing correction. Values represent the mean ± standard deviation (SD), along with minimum (Min) and maximum (Max) values, derived from 100 frames extracted at 1 ns intervals.

**Table 5:** In silico ADMET properties and drug-likeness parameters of the synthesized compounds.

Compounds	logPo/w	logS	logHERG	PCaco	logBB	logKhsa	PHOA	RoF
**1**	2.46	−2.71	−3.96	4392.03	0.57	−0.21	100	0
**2**	3.00	−3.43	−4.30	2607.29	0.10	−0.11	100	0
**3**	1.34	−2.68	−4.12	533.59	−0.49	−0.41	83.59	0
**4**	3.75	−5.75	−6.43	262.31	−1.30	0.39	92.20	0
**5**	3.73	−5.76	−6.44	255.30	−1.32	0.39	91.88	0
**6**	3.82	−5.80	−6.49	292.16	−1.24	0.39	93.43	0
**7**	3.71	−5.33	−6.40	650.85	−0.84	0.27	100	0
**8**	3.69	−5.33	−6.40	635.04	−0.86	0.26	100	0
**9**	3.87	−5.27	−6.39	1066.76	−0.58	0.25	100	0
**10**	4.77	−6.19	−6.43	2156.99	−0.22	0.60	100	0
**11**	4.75	−6.19	−6.44	2099.33	−0.24	0.60	100	0
**12**	4.57	−5.66	−6.04	1999.44	−0.22	0.54	100	0
**13**	4.59	−5.75	−6.39	2483.57	−0.21	0.45	100	0
**14**	4.49	−5.76	−6.37	2104.59	−0.29	0.43	100	0
**15**	3.47	−4.99	−6.32	1159.95	−0.49	0.12	100	0
**16**	3.46	−4.99	−6.33	1131.67	−0.51	0.12	100	0
**17**	4.99	−6.66	−6.62	2104.59	−0.33	0.64	100	0
**18**	4.96	−6.37	−6.44	2158.13	−0.05	0.56	100	0
**19**	5.04	−6.48	−6.46	2160.32	−0.03	0.59	100	1
Reference Range *	−2.0 to 6.5	−6.5 to 0.5	>−5.0	>500	−3.0 to 1.2	−1.5 to 1.5	>80%	≤4

* Reference ranges represent the 95% limits of known drugs as defined by the QikProp module.

## Data Availability

The original contributions presented in this study are included in the article/Supplementary Material. Further inquiries can be directed to the corresponding author.

## Supplementary Materials

The following supporting information can be downloaded at: https://www.mdpi.com/article/10.3390/ph19081270/s1, Figure S1: ^1^H-NMR spectrum of compound **4** (DMSO-*d*_6_); Figure S2: ^13^C-NMR (APT) spectrum of compound **4** (DMSO-*d*_6_); Figure S3: ^1^H-NMR spectrum of compound **5** (DMSO-*d*_6_); Figure S4: ^13^C-NMR (APT) spectrum of compound **5** (DMSO-*d*_6_); Figure S5: ^1^H-NMR spectrum of compound **6** (DMSO-*d*_6_); Figure S6: ^13^C-NMR (APT) spectrum of compound **6** (DMSO-*d*_6_); Figure S7: ^1^H-NMR spectrum of compound **7** (DMSO-*d*_6_); Figure S8: ^13^C-NMR (APT) spectrum of compound **7** (DMSO-*d*_6_); Figure S9: ^1^H-NMR spectrum of compound **8** (DMSO-*d*_6_); Figure S10: ^13^C-NMR (APT) spectrum of compound **8** (DMSO-*d*_6_); Figure S11: ^1^H-NMR spectrum of compound **9** (DMSO-*d*_6_); Figure S12: ^13^C-NMR (APT) spectrum of compound **9** (DMSO-*d*_6_); Figure S13: ^1^H-NMR spectrum of compound **11** (DMSO-*d*_6_); Figure S14: ^13^C-NMR (APT) spectrum of compound **11** (DMSO-*d*_6_); Figure S15: ^1^H-NMR spectrum of compound **12** (DMSO-*d*_6_); Figure S16: ^13^C-NMR (APT) spectrum of compound **12** (DMSO-*d*_6_); Figure S17: ^1^H-NMR spectrum of compound **13** (DMSO-*d*_6_); Figure S18: ^13^C-NMR (APT) spectrum of compound **13** (DMSO-*d*_6_); Figure S19: ^1^H-NMR spectrum of compound **14** (DMSO-*d*_6_); Figure S20: ^13^C-NMR (APT) spectrum of compound **14** (DMSO-*d*_6_); Figure S21: ^1^H-NMR spectrum of compound **15** (DMSO-*d*_6_); Figure S22: ^13^C-NMR (APT) spectrum of compound **15** (DMSO-*d*_6_); Figure S23: ^1^H-NMR spectrum of compound **16** (DMSO-*d*_6_); Figure S24: ^13^C-NMR (APT) spectrum of compound **16** (DMSO-*d*_6_); Figure S25: ^1^H-NMR spectrum of compound **17** (DMSO-*d*_6_); Figure S26: ^13^C-NMR (APT) spectrum of compound **17** (DMSO-*d*_6_); Figure S27: ^1^H-NMR spectrum of compound **18** (DMSO-*d*_6_); Figure S28: ^13^C-NMR (APT) spectrum of compound **18** (DMSO-*d*_6_); Figure S29: ^1^H-NMR spectrum of compound **19** (DMSO-*d*_6_); Figure S30: ^13^C-NMR (APT) spectrum of compound **19** (DMSO-*d*_6_).
