# Supplementary material for: Trifluoromethyl-Benzimidazole-Hydrazone Derivatives as Promising Selective Anticancer Agents: Synthesis, Cytotoxicity, and Molecular Docking Insights
Source: Pharmaceuticals (Basel). 2026 Aug 11;19(8):1270. doi: 10.3390/ph19081270 (PMC13516195; doi:10.3390/ph19081270)
Supplement: Supplementary file 1 [file pharmaceuticals-19-01270-s001.zip › pharmaceuticals-4431778-supplementary.pdf]

Supporting Information

Trifluoromethyl Benzimidazole–Hydrazone Derivatives as Promising Selective Anticancer Agents: Synthesis, Cytotoxicity, and Molecular Docking Insights

Musa Özil*, Eyüp Önelge, Mustafa Emirik, Kübra Acikalin Cosku[2], Yusuf Tutar

Contents of Supporting Information:

Figure S1. ^{1}H-NMR spectrum of compound 4 (DMSO-d_6) 2
Figure S2. ^{13}C-NMR (APT) spectrum of compound 4 (DMSO-d_6) 2
Figure S3. ^{1}H-NMR spectrum of compound 5 (DMSO-d_6) 3
Figure S4. ^{13}C-NMR (APT) spectrum of compound 5 (DMSO-d_6) 3
Figure S5. ^{1}H-NMR spectrum of compound 6 (DMSO-d_6) 4
Figure S6. ^{13}C-NMR (APT) spectrum of compound 6 (DMSO-d_6) 4
Figure S7. ^{1}H-NMR spectrum of compound 7 (DMSO-d_6) 5
Figure S8. ^{13}C-NMR (APT) spectrum of compound 7 (DMSO-d_6) 5
Figure S9. ^{1}H-NMR spectrum of compound 8 (DMSO-d_6) 6
Figure S10. ^{13}C-NMR (APT) spectrum of compound 8 (DMSO-d_6) 6
Figure S11. ^{1}H-NMR spectrum of compound 9 (DMSO-d_6) 7
Figure S12. ^{13}C-NMR (APT) spectrum of compound 9 (DMSO-d_6) 7
Figure S13. ^{1}H-NMR spectrum of compound 11 (DMSO-d_6) 8
Figure S14. ^{13}C-NMR (APT) spectrum of compound 11 (DMSO-d_6) 8
Figure S15. ^{1}H-NMR spectrum of compound 12 (DMSO-d_6) 9
Figure S16. ^{13}C-NMR (APT) spectrum of compound 12 (DMSO-d_6) 9
Figure S17. ^{1}H-NMR spectrum of compound 13 (DMSO-d_6) 10
Figure S18. ^{13}C-NMR (APT) spectrum of compound 13 (DMSO-d_6) 10
Figure S19. ^{1}H-NMR spectrum of compound 14 (DMSO-d_6) 11
Figure S20. ^{13}C-NMR (APT) spectrum of compound 14 (DMSO-d_6) 11
Figure S21. ^{1}H-NMR spectrum of compound 15 (DMSO-d_6) 12
Figure S22. ^{13}C-NMR (APT) spectrum of compound 15 (DMSO-d_6) 12
Figure S23. ^{1}H-NMR spectrum of compound 16 (DMSO-d_6) 13
Figure S24. ^{13}C-NMR (APT) spectrum of compound 16 (DMSO-d_6) 13
Figure S25. ^{1}H-NMR spectrum of compound 17 (DMSO-d_6) 14
Figure S26. ^{13}C-NMR (APT) spectrum of compound 17 (DMSO-d_6) 14
Figure S27. ^{1}H-NMR spectrum of compound 18 (DMSO-d_6) 15
Figure S28. ^{13}C-NMR (APT) spectrum of compound 18 (DMSO-d_6) 15
Figure S29. ^{1}H-NMR spectrum of compound 19 (DMSO-d_6) 16
Figure S30. ^{13}C-NMR (APT) spectrum of compound 19 (DMSO-d_6) 16

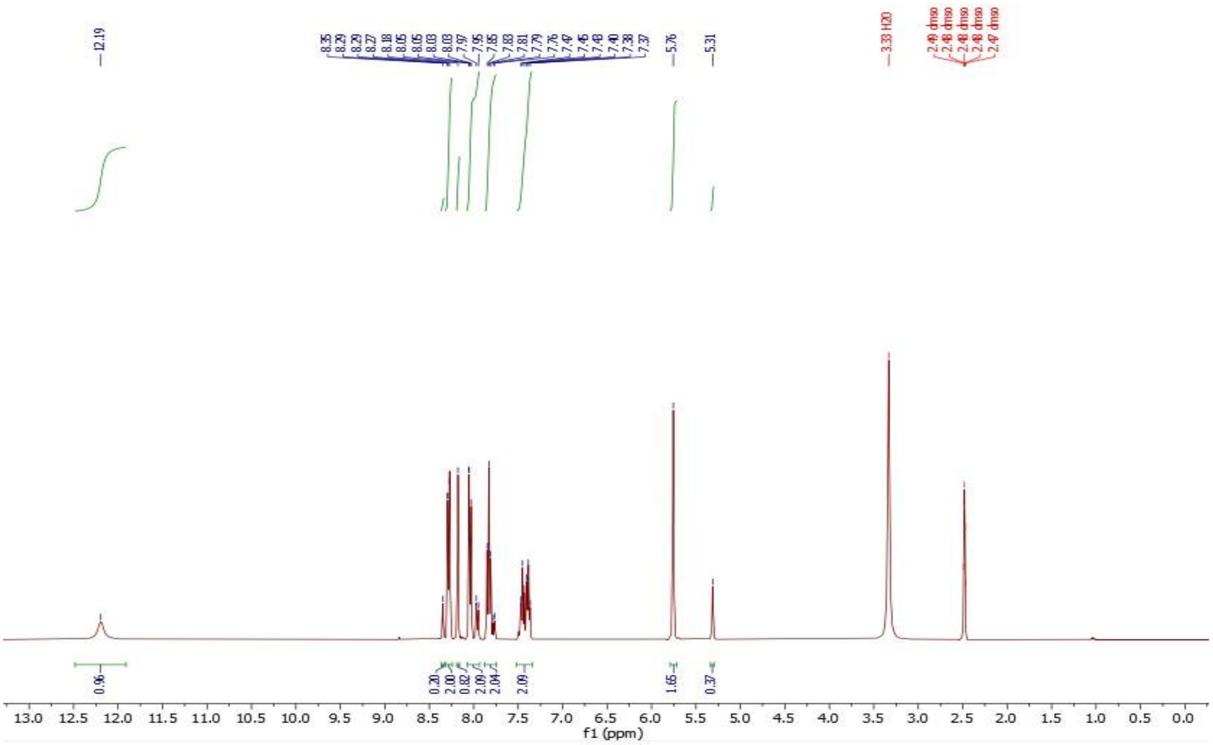

Figure S1. ^{1}H-NMR spectrum of compound **4** (DMSO-d_6)

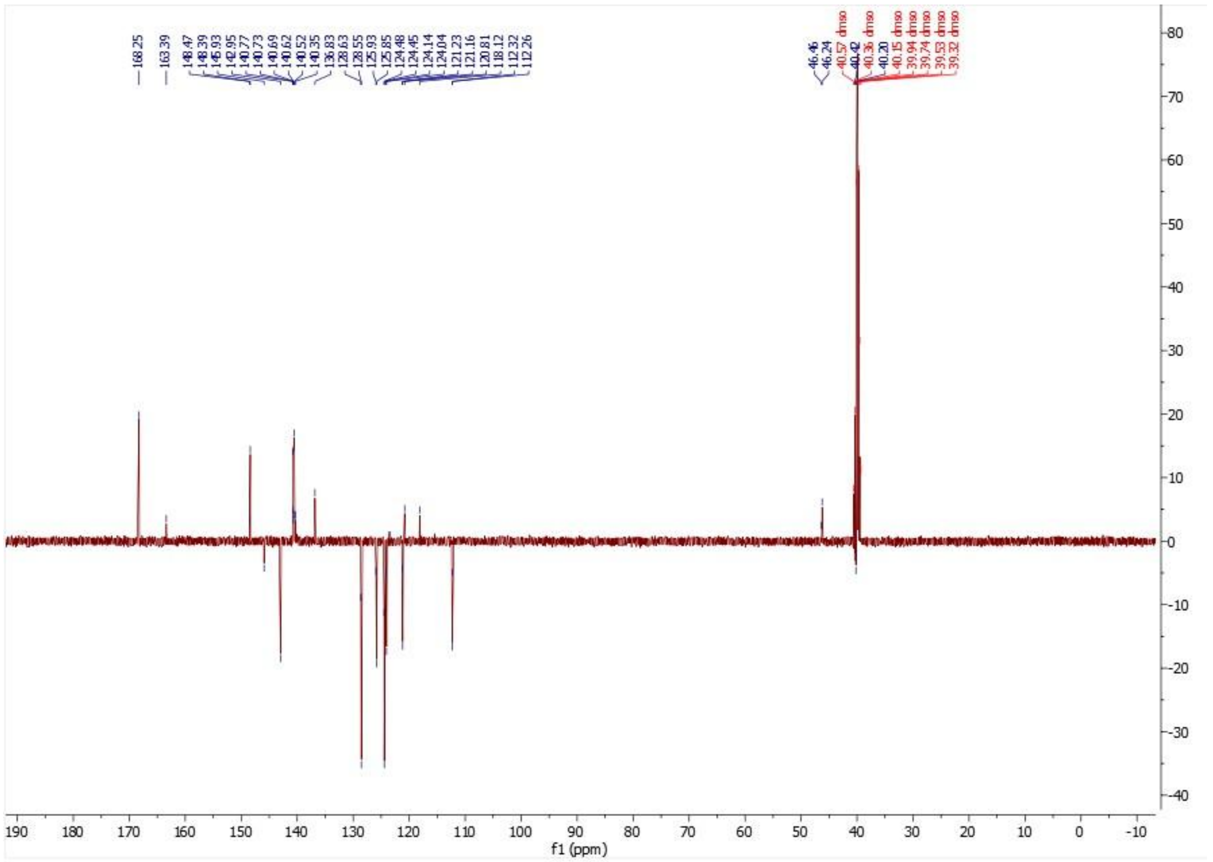

Figure S2. ^{13}C-NMR (APT) spectrum of compound **4** (DMSO-d_6)

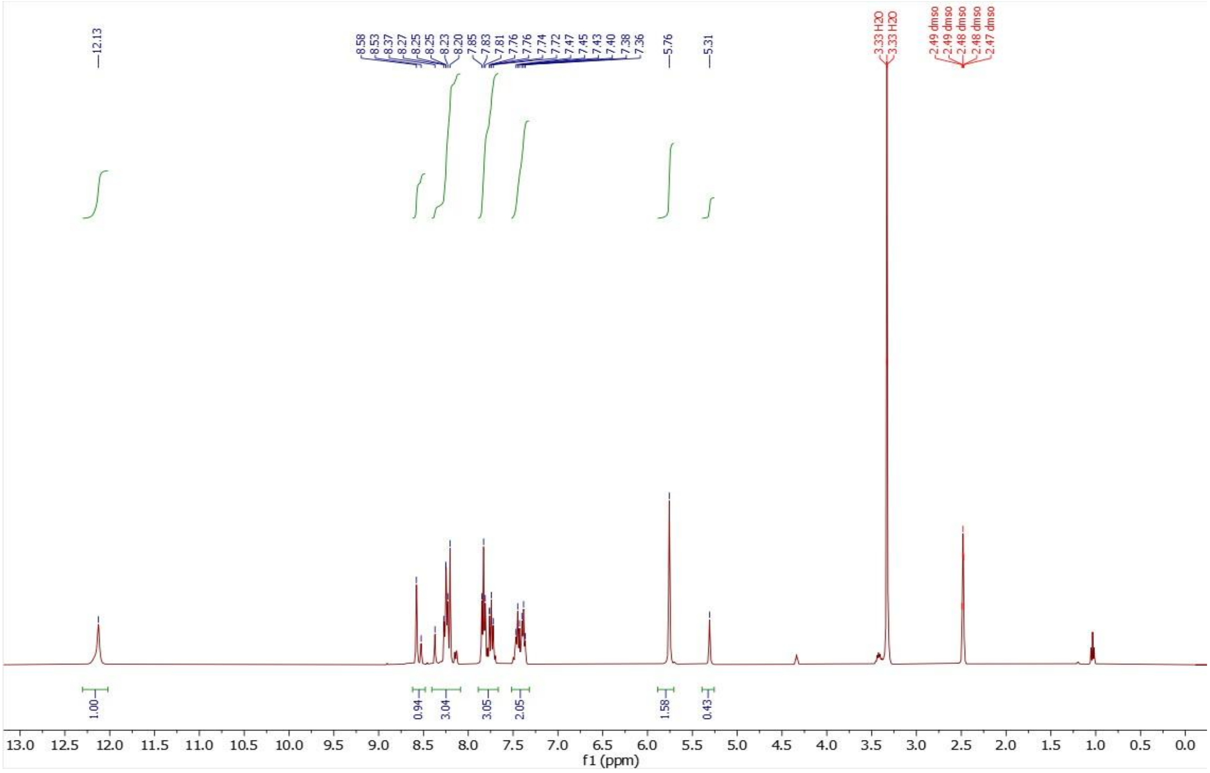

Figure S3. ^{1}H-NMR spectrum of compound **5** (DMSO-d_6)

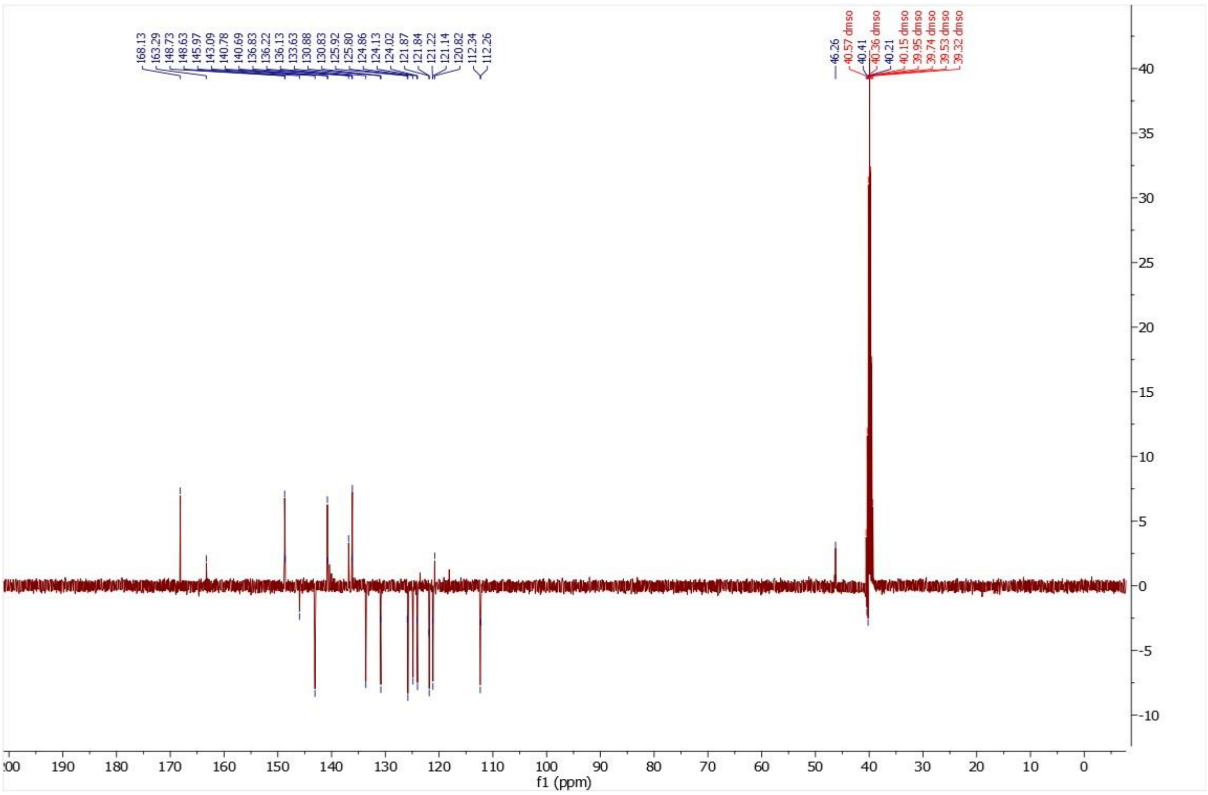

Figure S4. ^{13}C-NMR (APT) spectrum of compound **5** (DMSO-d_6)

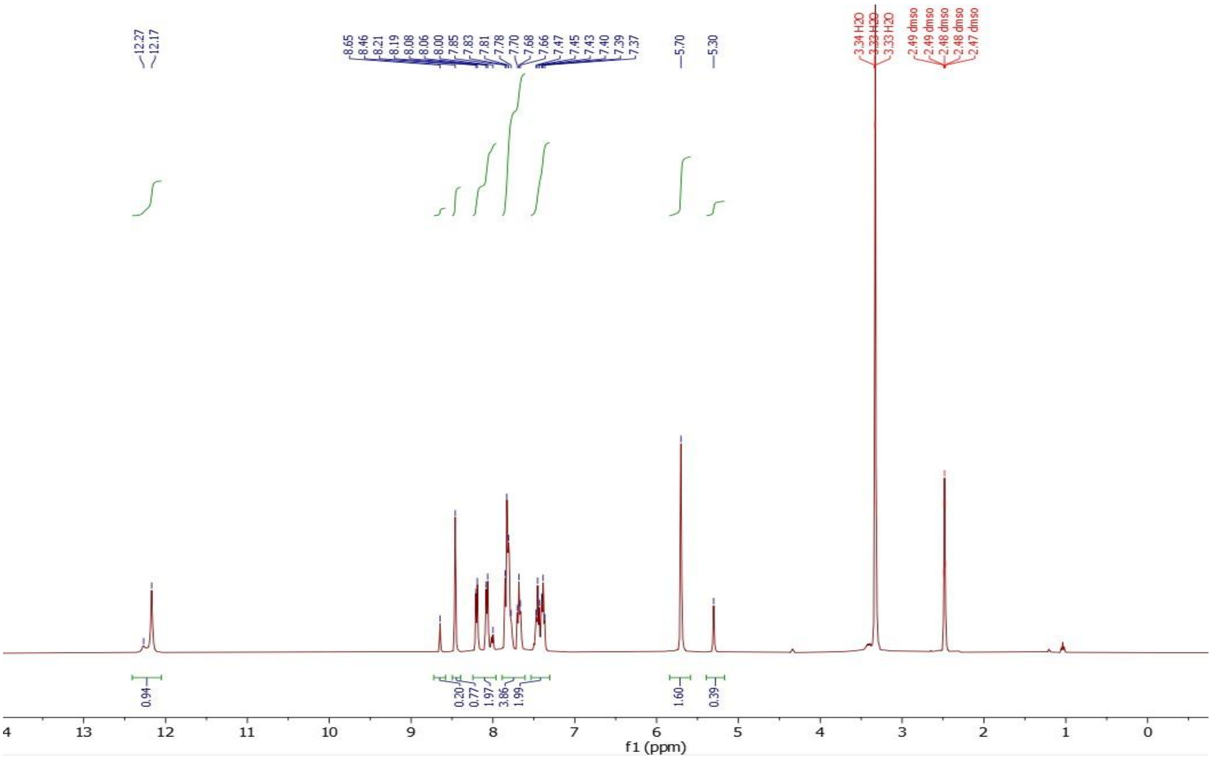

Figure S5. ^{1}H-NMR spectrum of compound **6** (DMSO-d_6)

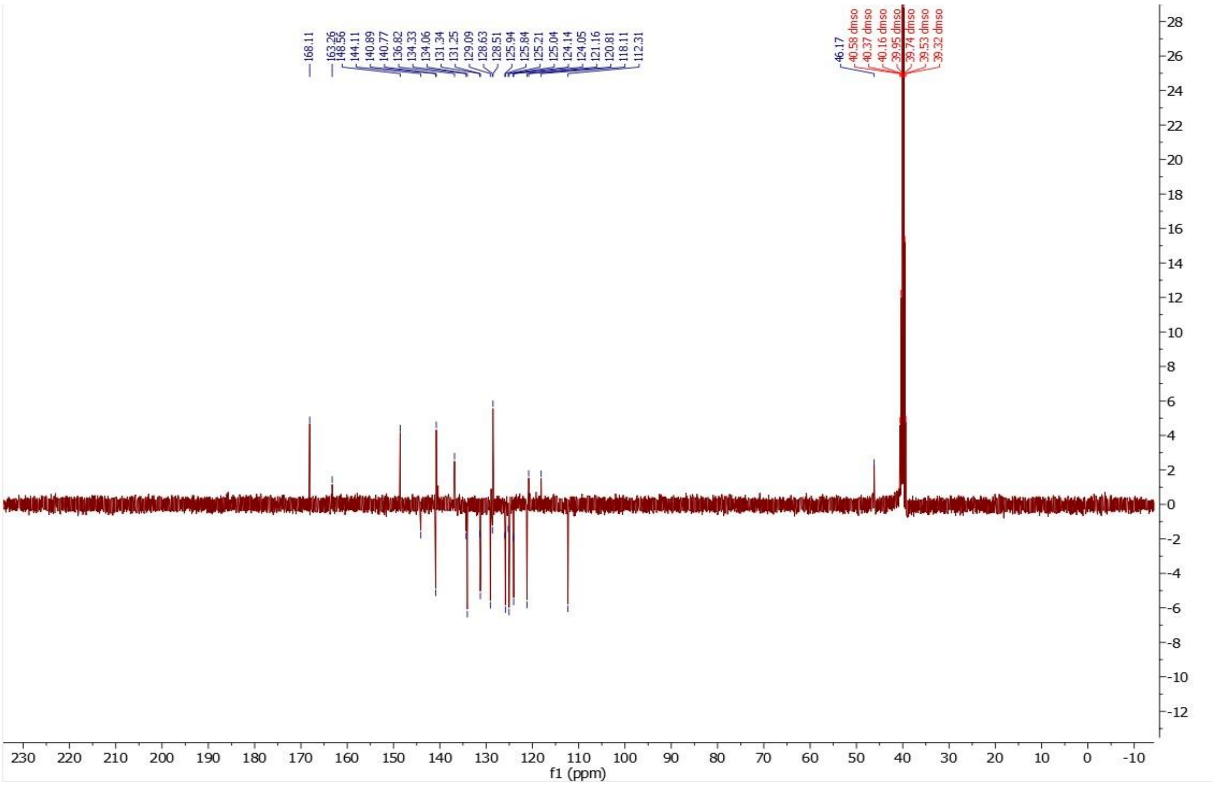

Figure S6. ^{13}C-NMR (APT) spectrum of compound **6** (DMSO-d_6)

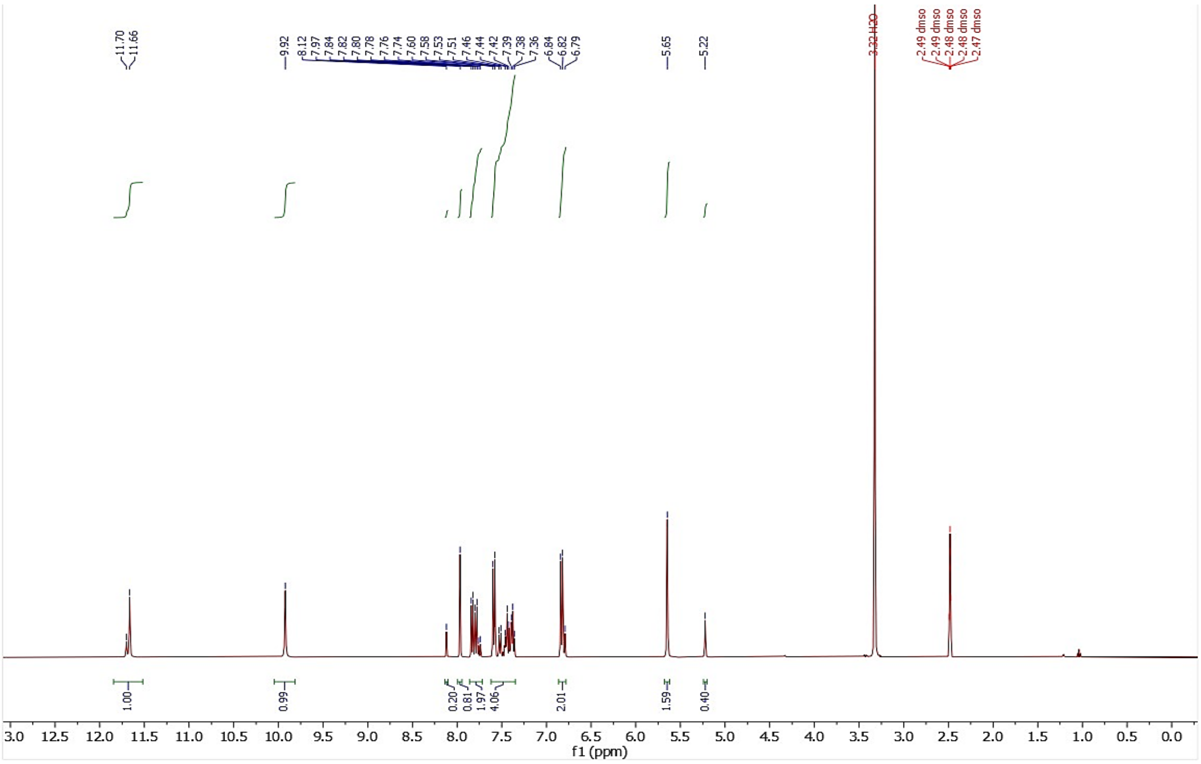

Figure S7. ^{1}H-NMR spectrum of compound **7** (DMSO-d_6)

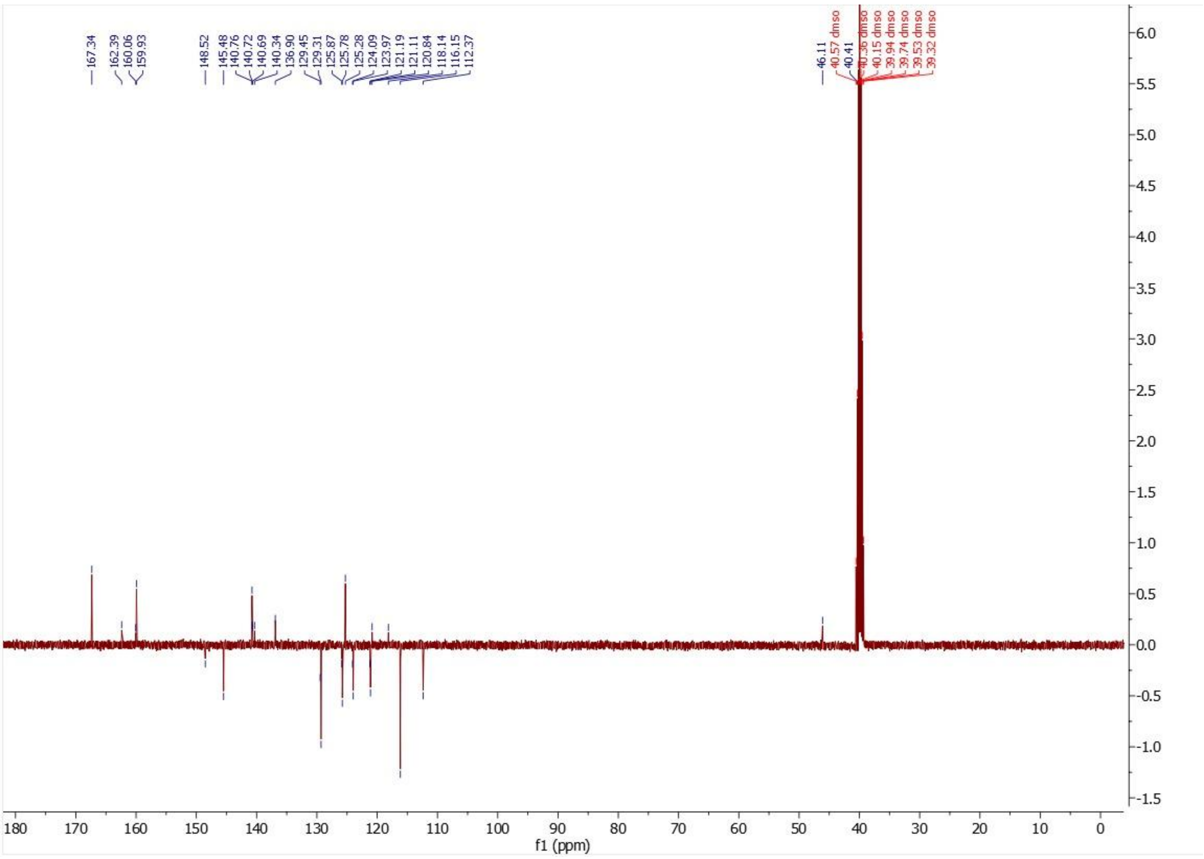

Figure S8. ^{13}C-NMR (APT) spectrum of compound **7** (DMSO-d_6)

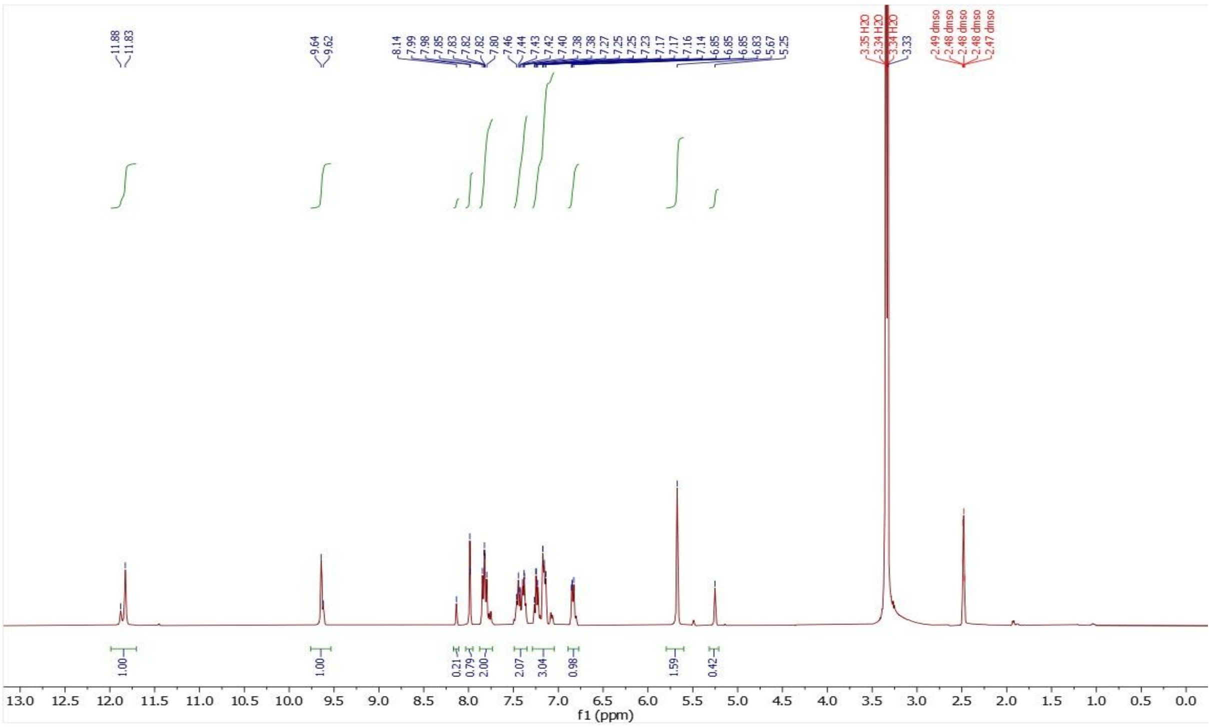

Figure S9. ^{1}H-NMR spectrum of compound **8** (DMSO-d$_6$)

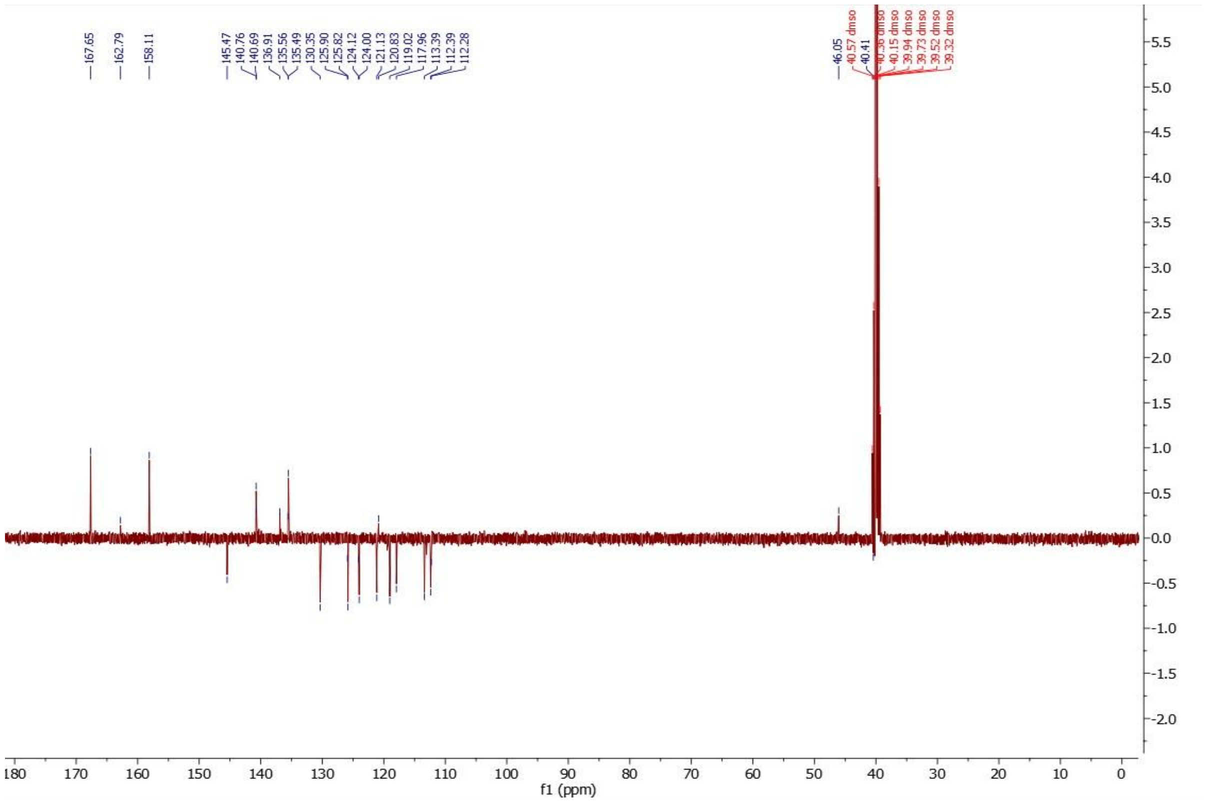

Figure S10. ^{13}C-NMR (APT) spectrum of compound **8** (DMSO-d$_6$)

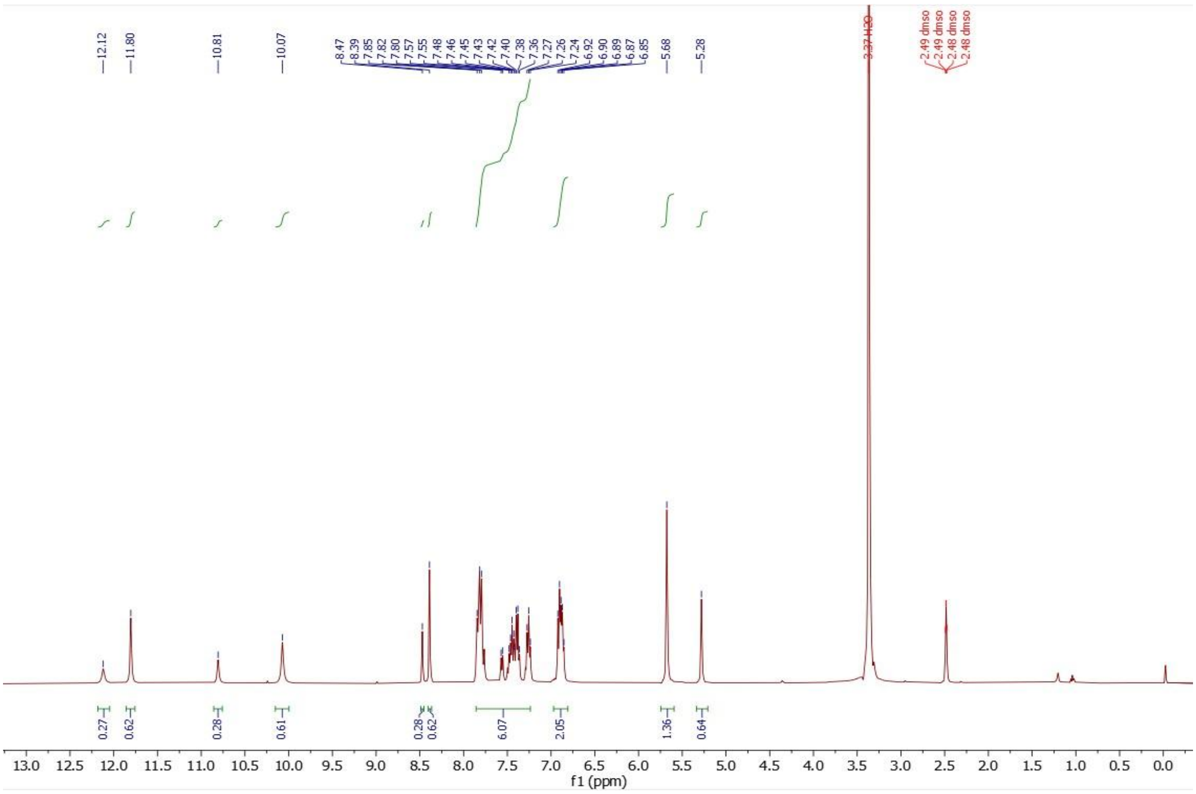

Figure S11. ^{1}H-NMR spectrum of compound **9** (DMSO-d_6)

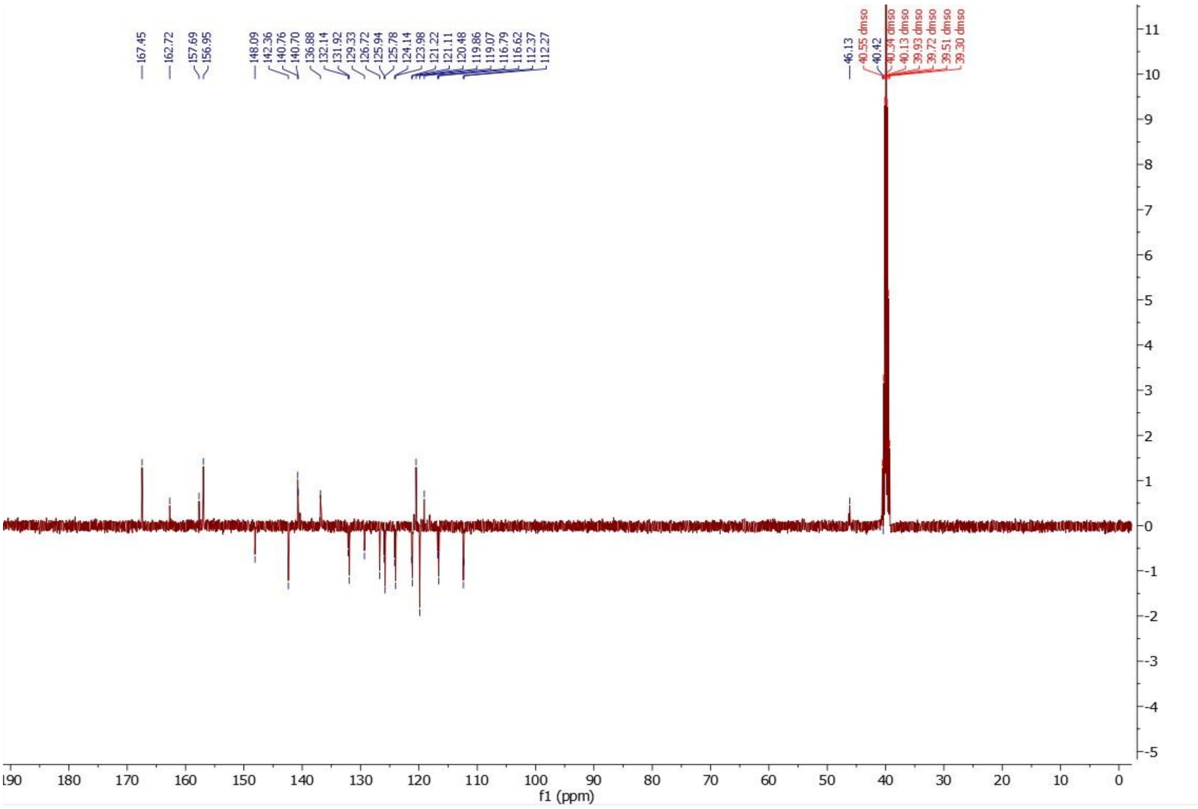

Figure S12. ^{13}C-NMR (APT) spectrum of compound **9** (DMSO-d_6)

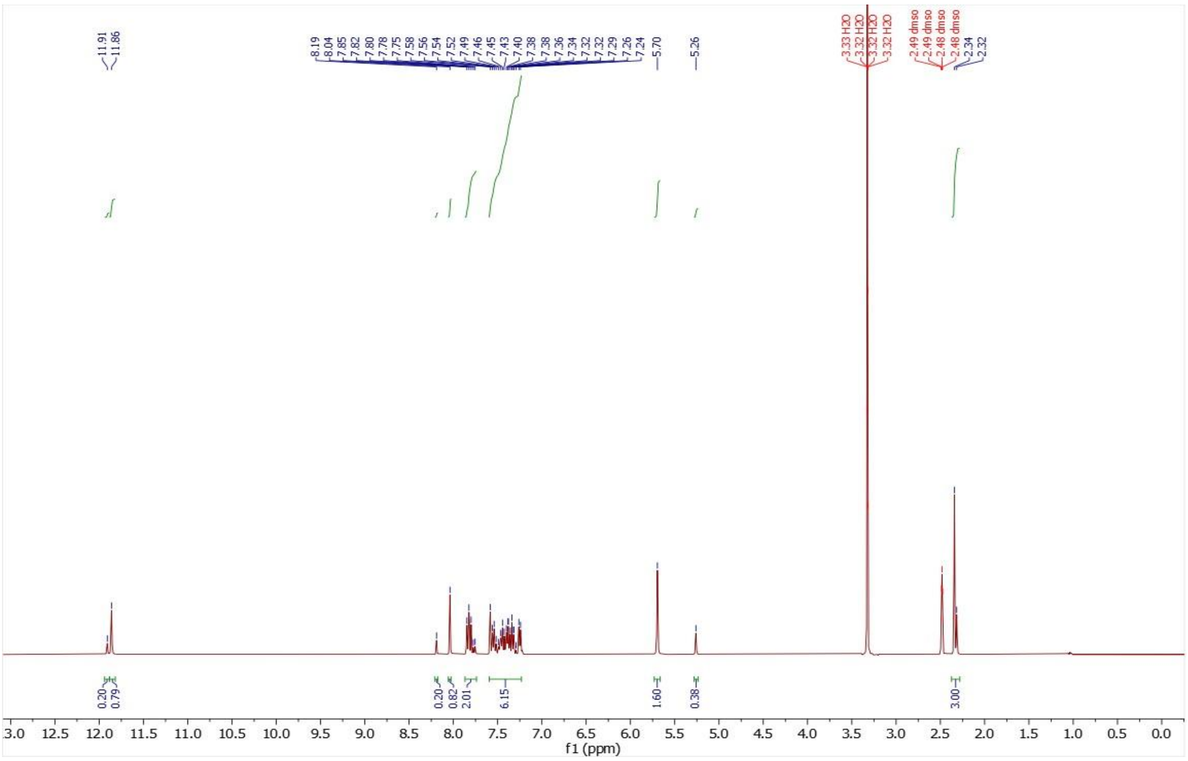

Figure S13. ^{1}H-NMR spectrum of compound **11** (DMSO-d_6)

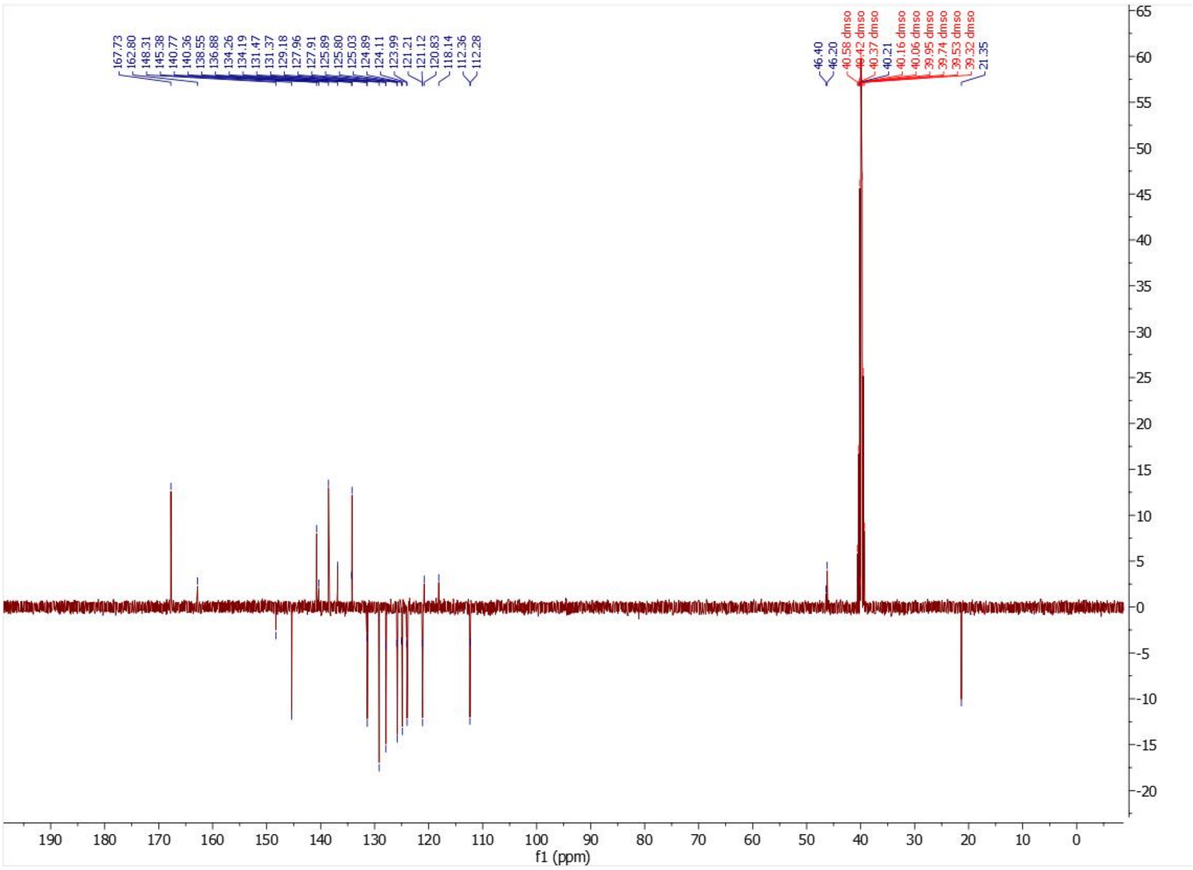

Figure S14. ^{13}C-NMR (APT) spectrum of compound **11** (DMSO-d_6)

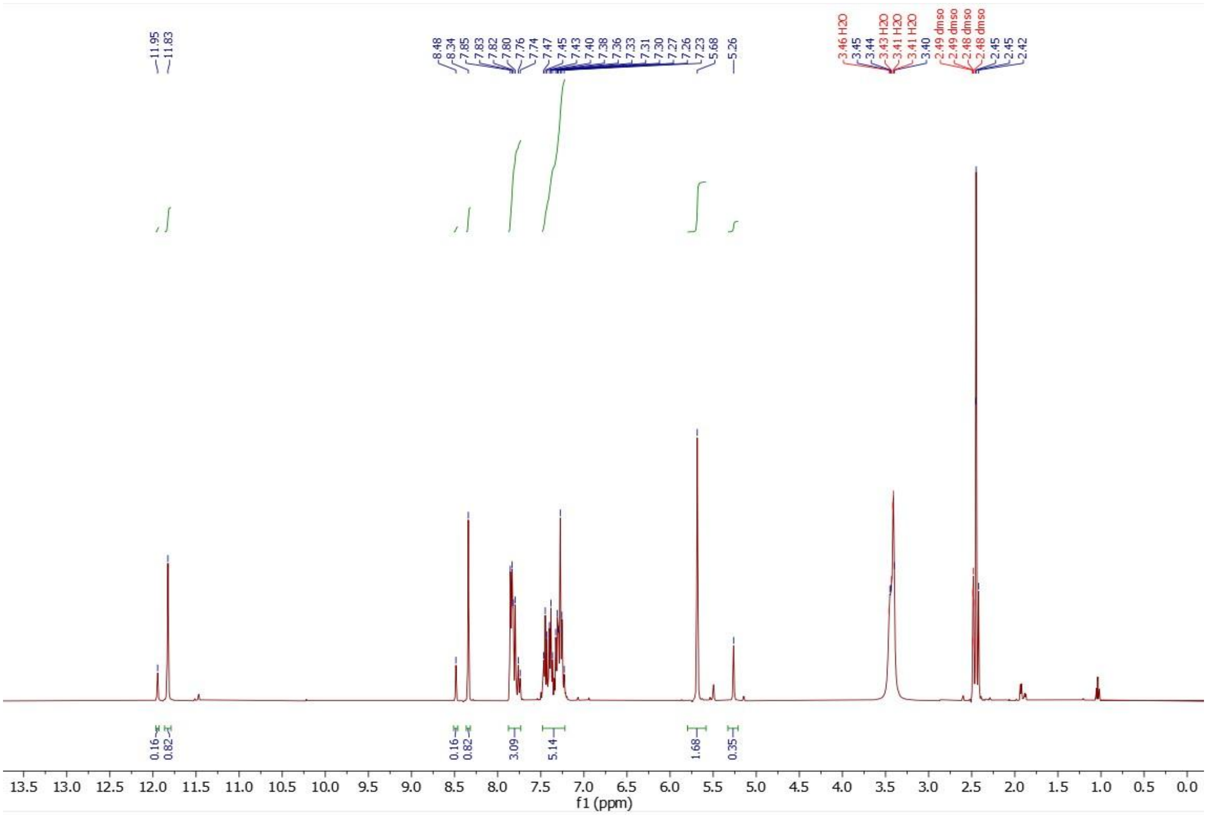

Figure S15. ^{1}H-NMR spectrum of compound **12** (DMSO-d$_6$)

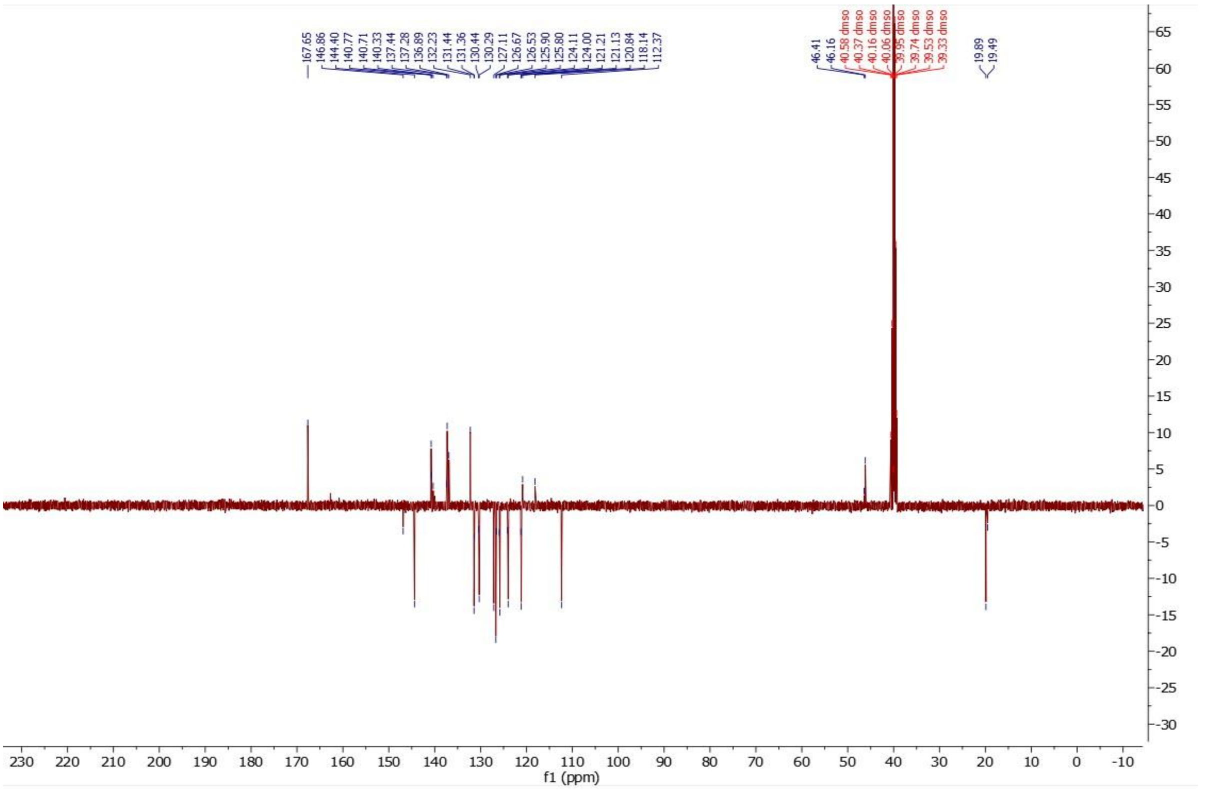

Figure S16. ^{13}C-NMR (APT) spectrum of compound **12** (DMSO-d$_6$)

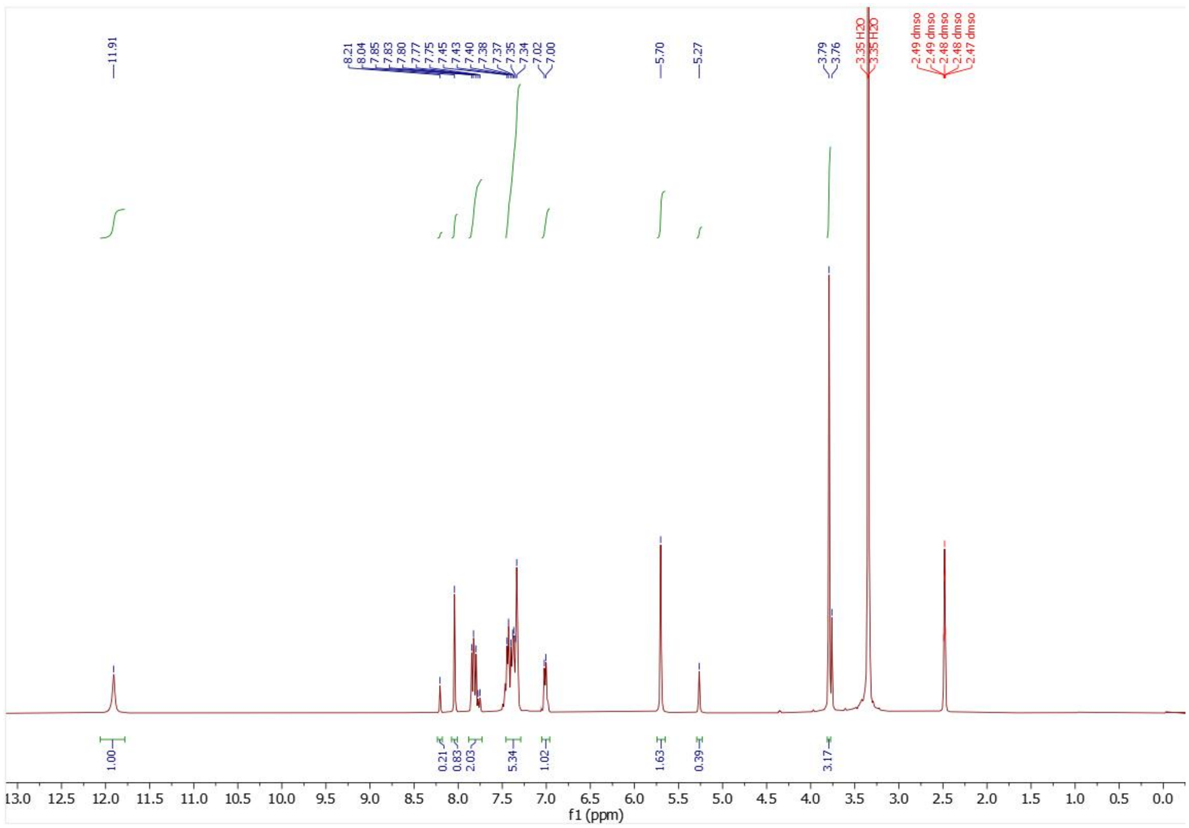

Figure S17. ^{1}H-NMR spectrum of compound **13** (DMSO-d_6)

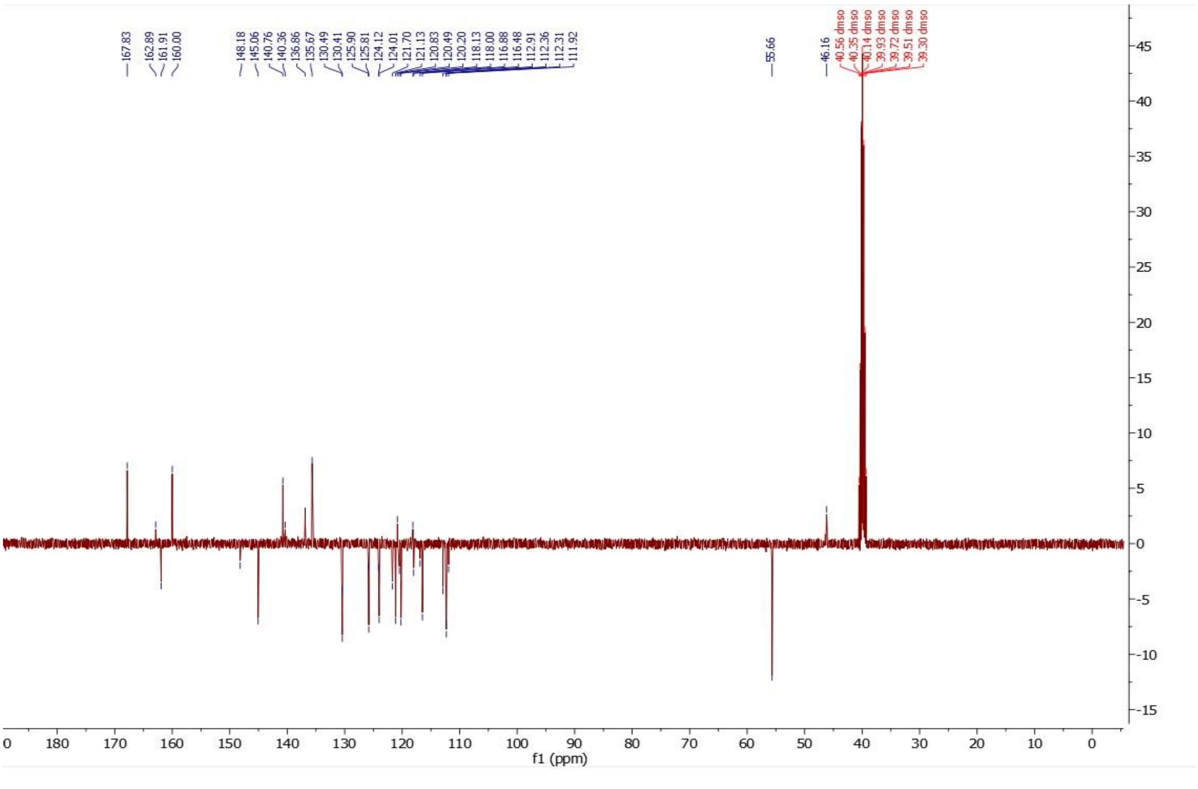

Figure S18. ^{13}C-NMR (APT) spectrum of compound **13** (DMSO-d_6)

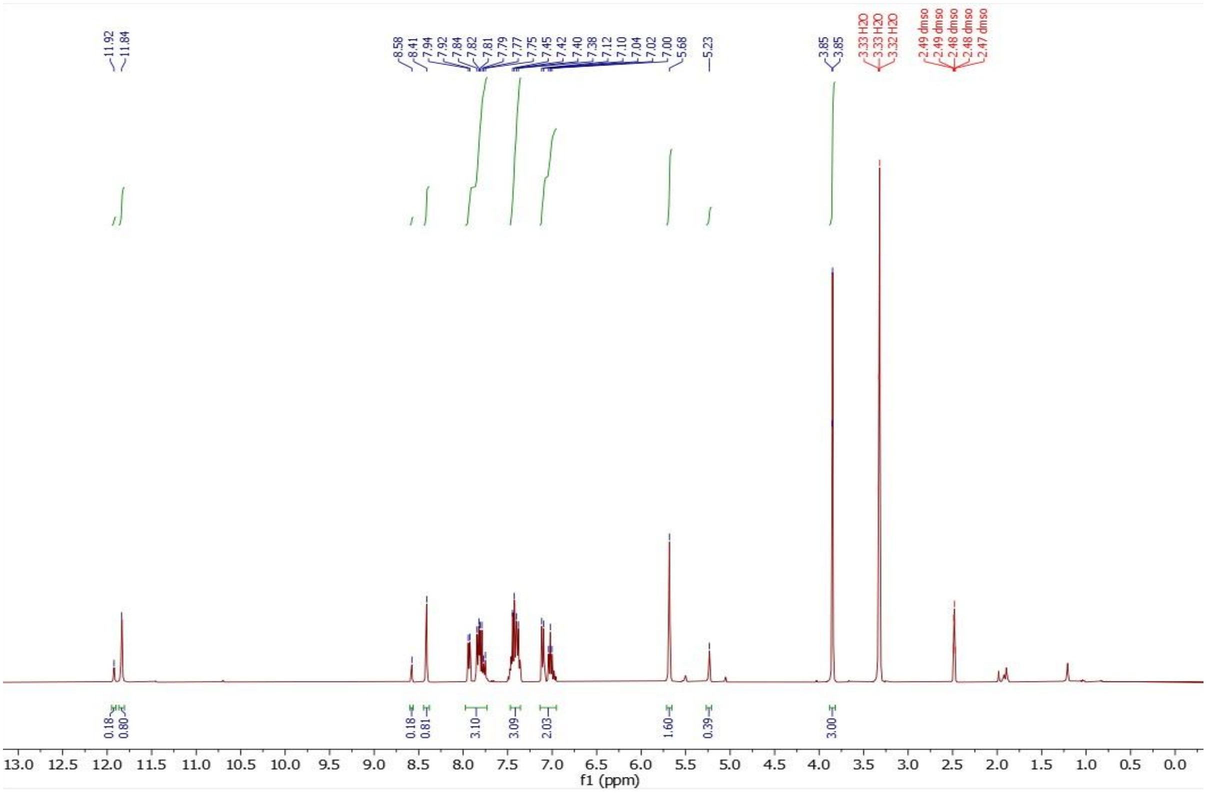

Figure S19. ^{1}H-NMR spectrum of compound **14** (DMSO-d_6)

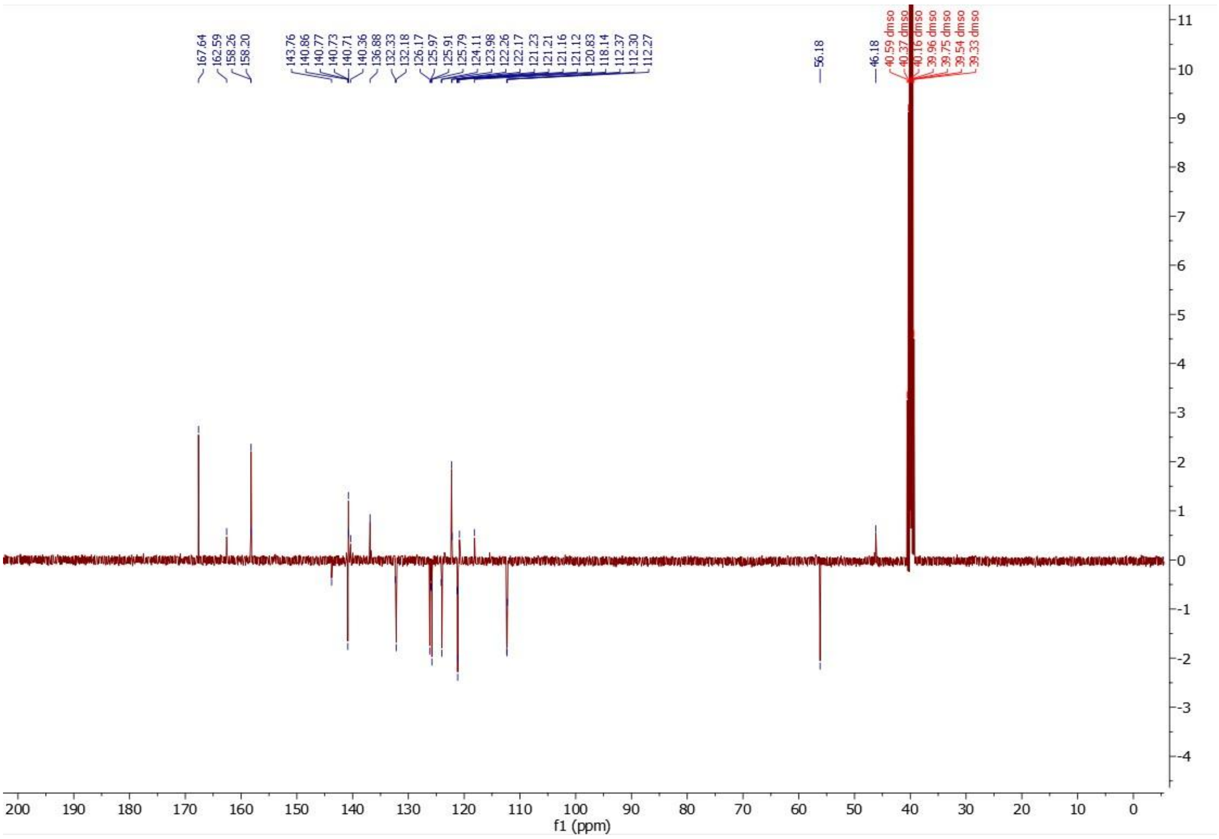

Figure S20. ^{13}C-NMR (APT) spectrum of compound **14** (DMSO-d_6)

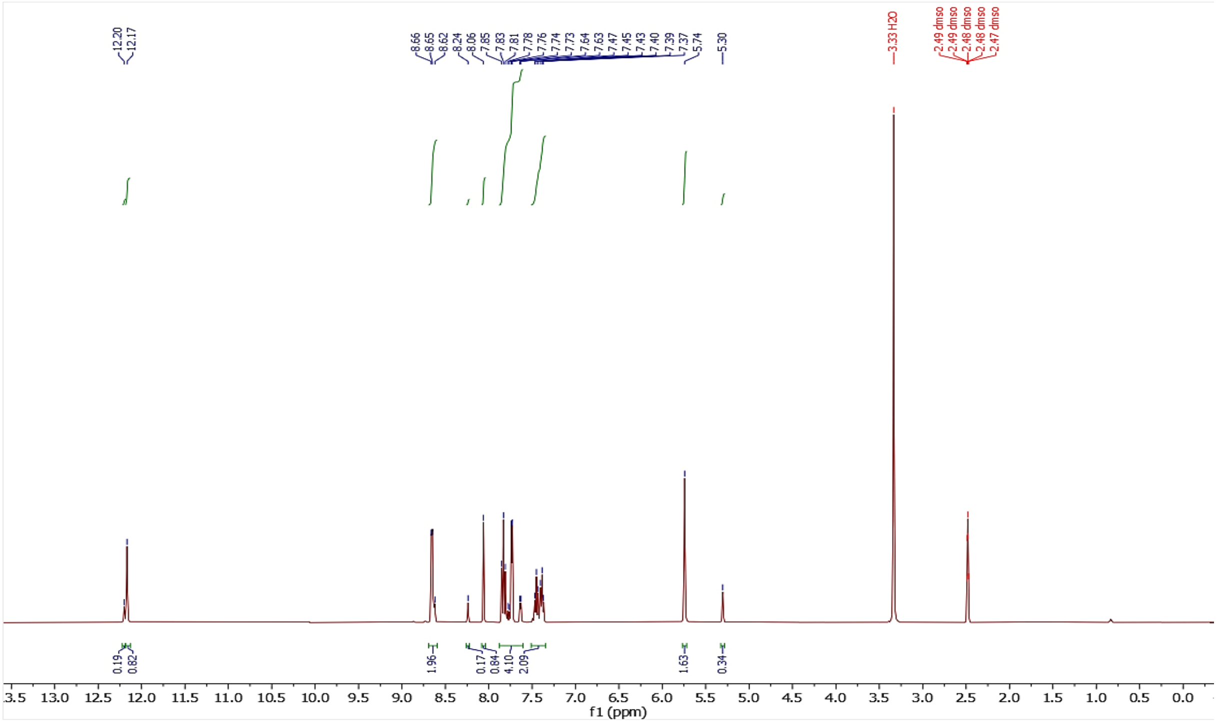

Figure S21. ^{1}H-NMR spectrum of compound **15** (DMSO-d_6)

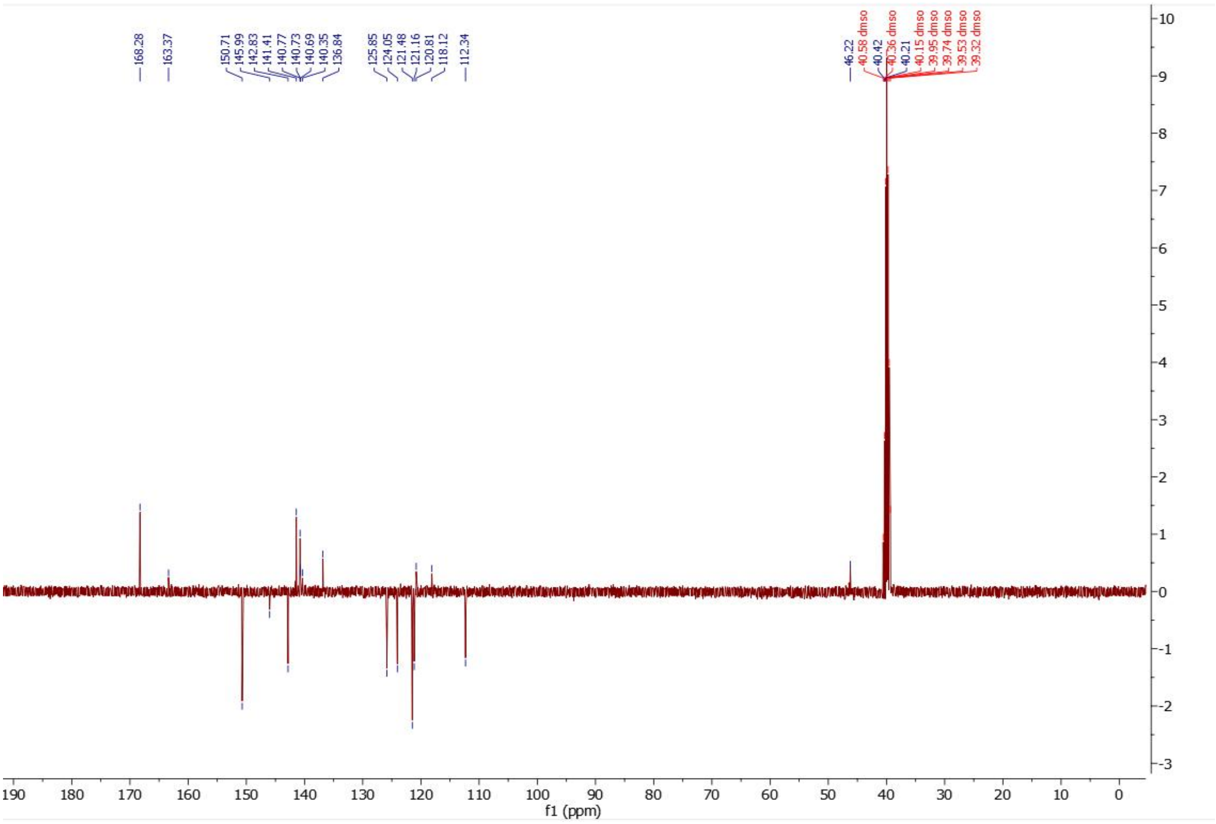

Figure S22. ^{13}C-NMR (APT) spectrum of compound **15** (DMSO-d_6)

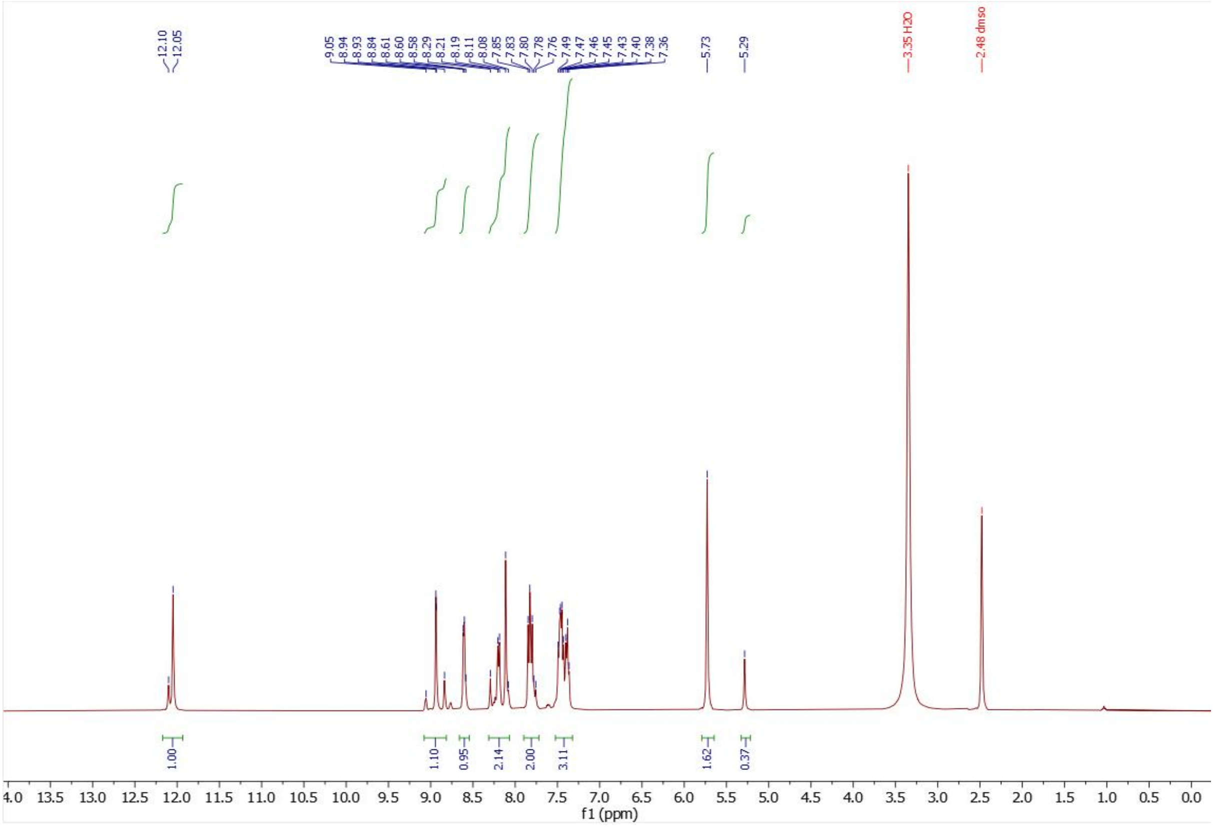

Figure S23. ^{1}H-NMR spectrum of compound **16** (DMSO-d_6)

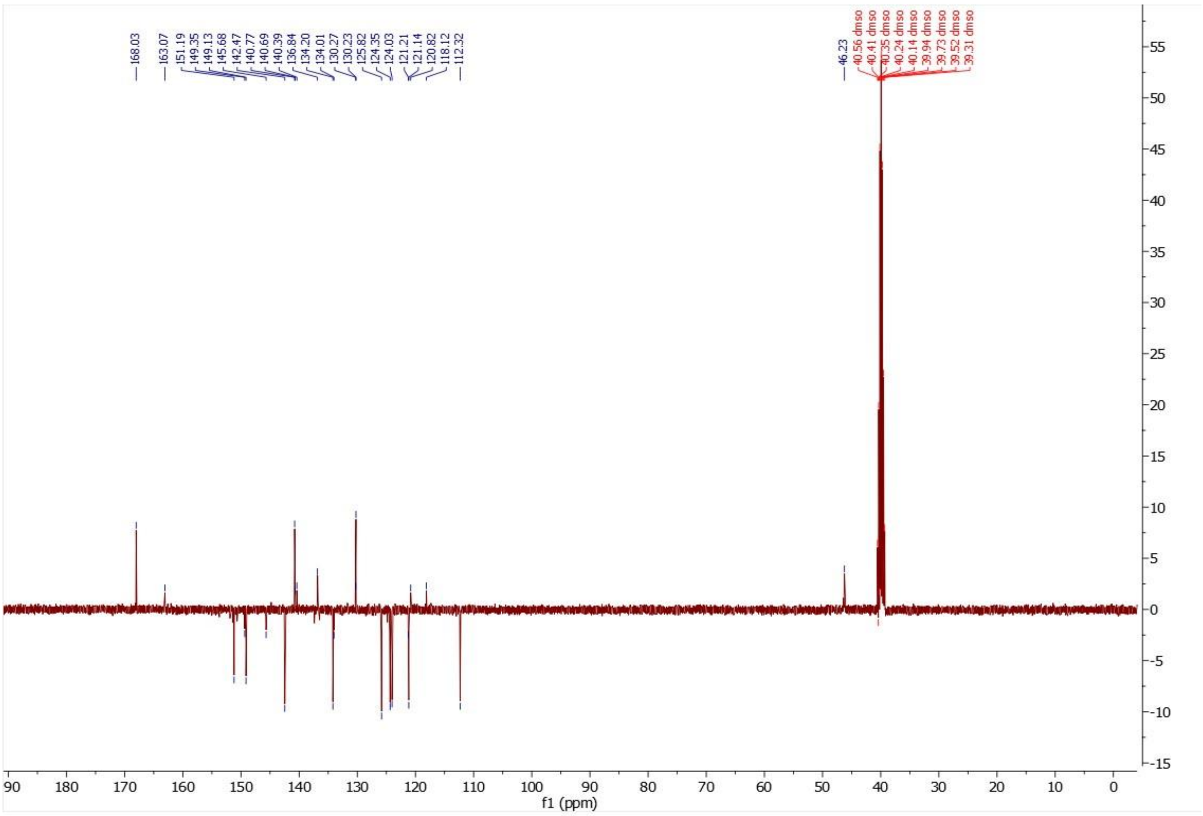

Figure S24. ^{13}C-NMR (APT) spectrum of compound **16** (DMSO-d_6)

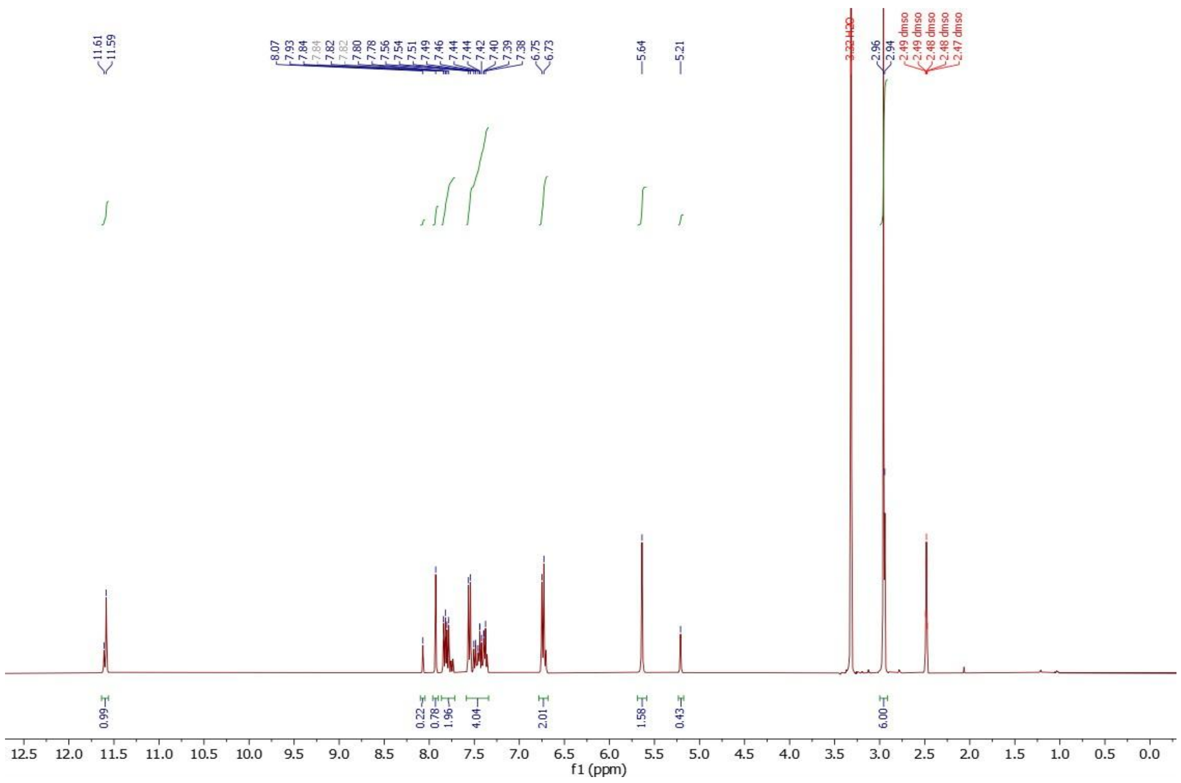

Figure S25. ^{1}H-NMR spectrum of compound **17** (DMSO-d$_6$)

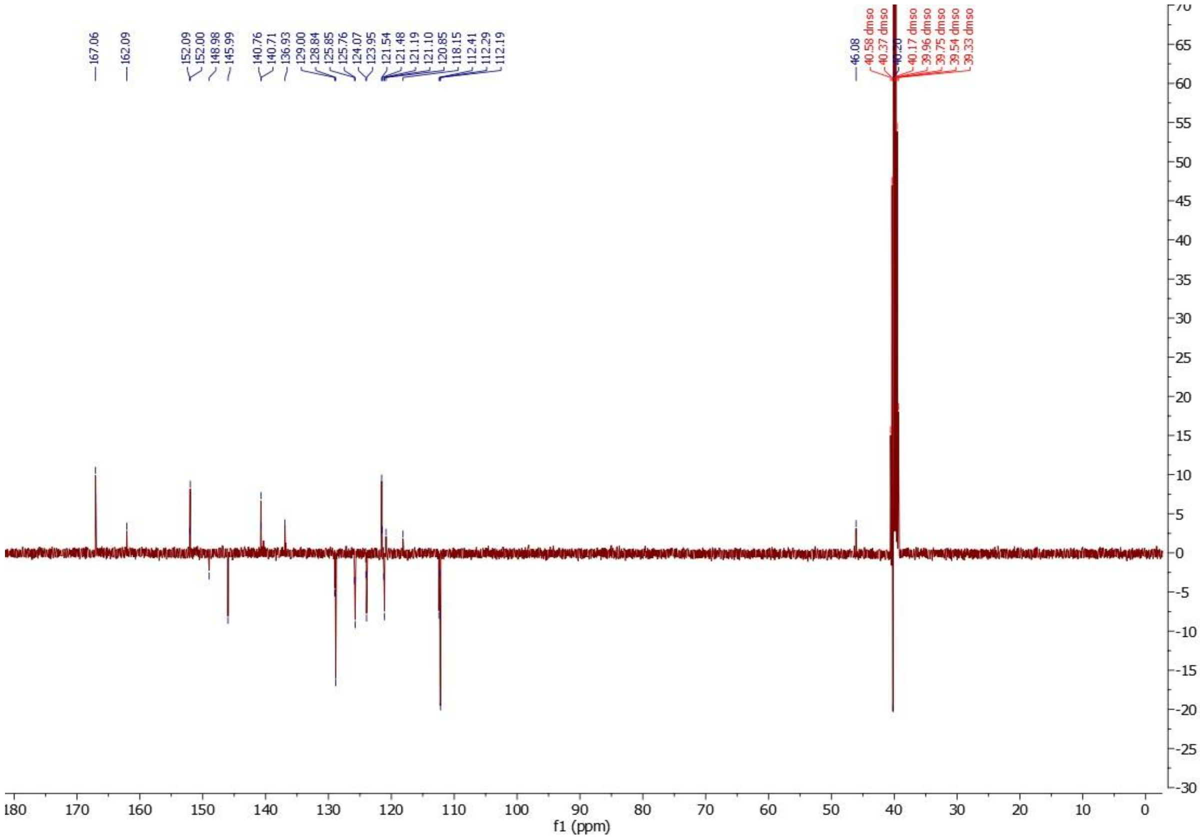

Figure S26. ^{13}C-NMR (APT) spectrum of compound **17** (DMSO-d$_6$)

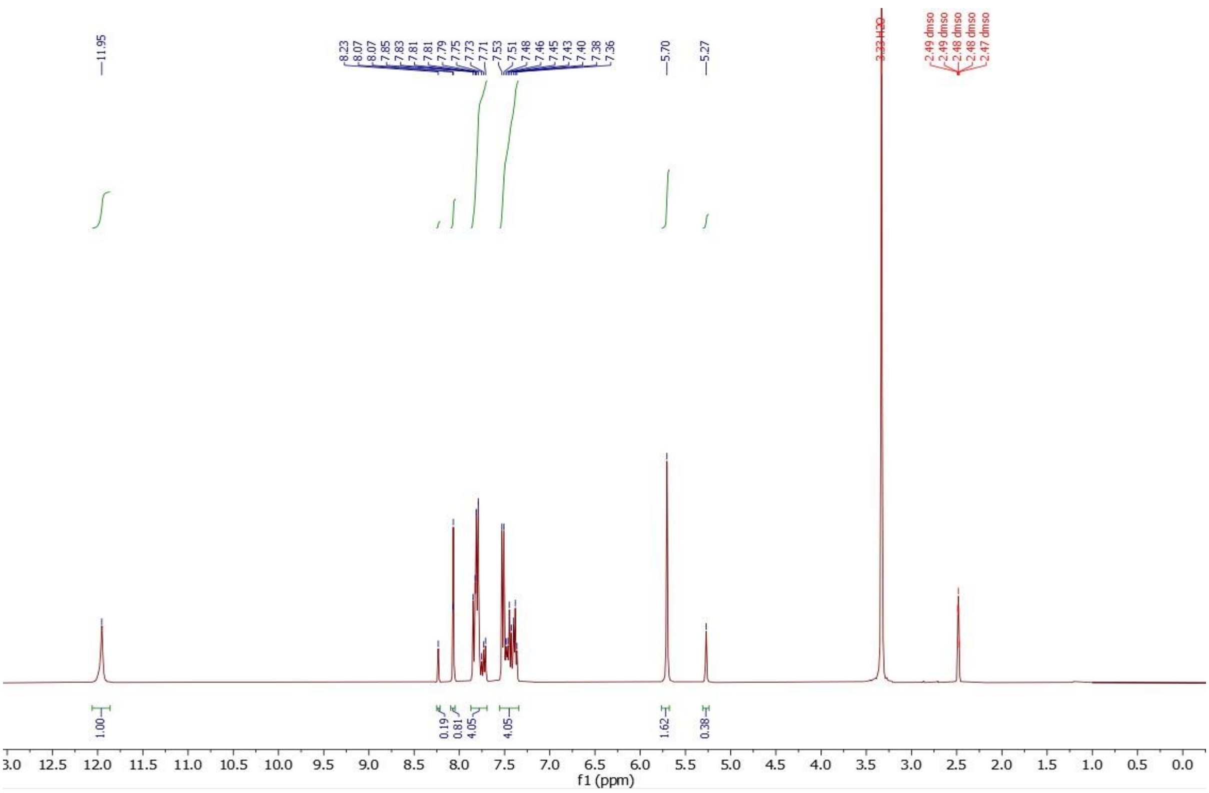

Figure S27. ^{1}H-NMR spectrum of compound **18** (DMSO-d_6)

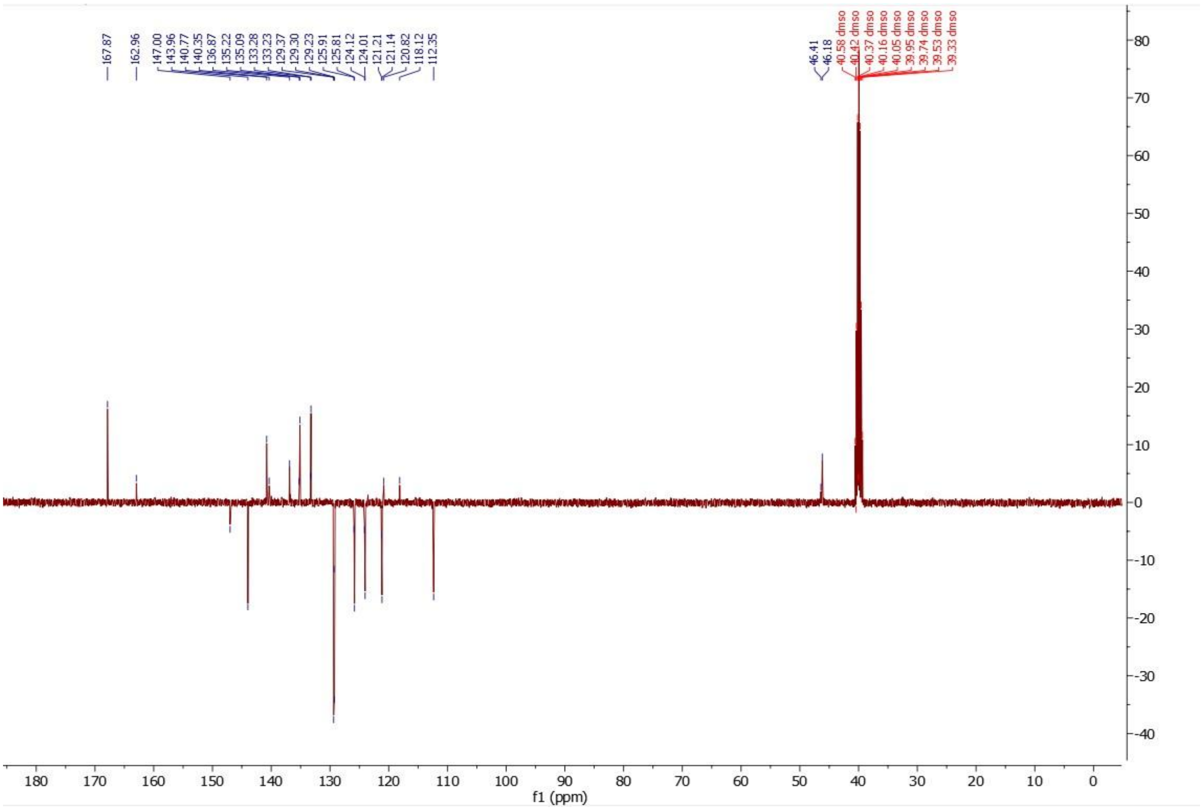

Figure S28. ^{13}C-NMR (APT) spectrum of compound **18** (DMSO-d_6)

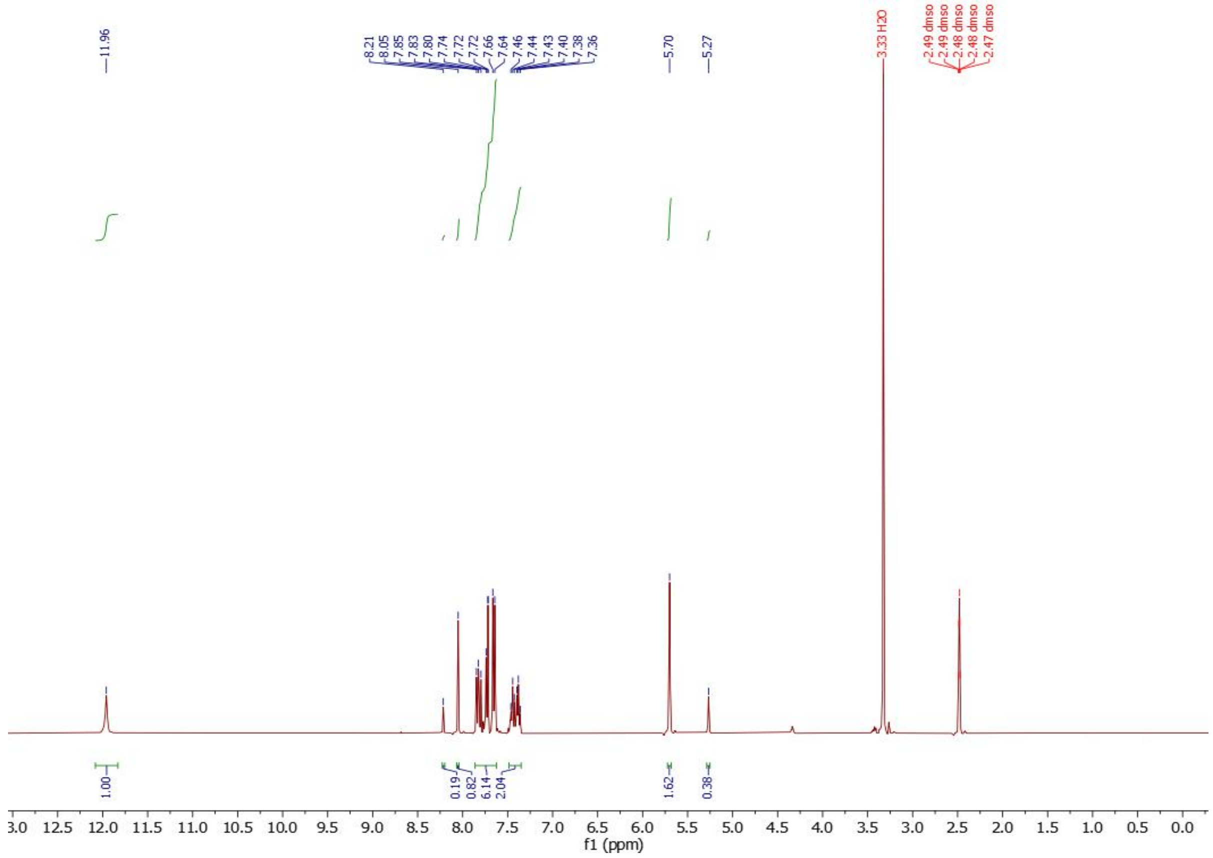

Figure S29. ^{1}H-NMR spectrum of compound **19** (DMSO-d_6)

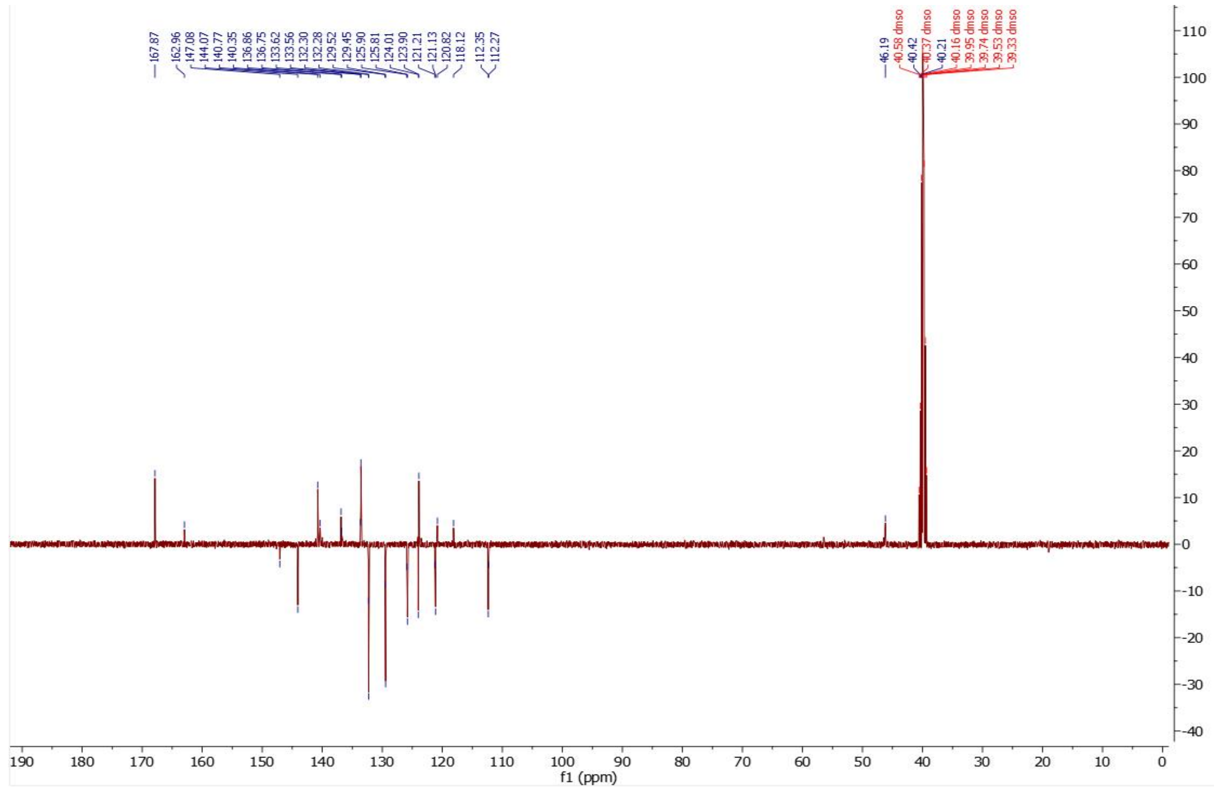

Figure S30. ^{13}C-NMR (APT) spectrum of compound **19** (DMSO-d_6)
